# Endogenous mutant Huntingtin alters the corticogenesis via lowering Golgi recruiting ARF1 in cortical organoid

**DOI:** 10.1038/s41380-024-02562-0

**Published:** 2024-04-23

**Authors:** Yang Liu, Xinyu Chen, Yunlong Ma, Chenyun Song, Jixin Ma, Cheng Chen, Jianzhong Su, Lixiang Ma, Hexige Saiyin

**Affiliations:** 1 Department of Anatomy and Histology & Embryology, School of Basic Medical Sciences, Fudan University, Shanghai, 200032 China; 2 Oujiang Laboratory (Zhejiang Lab for Regenerative Medicine, Vision and Brain Health), Eye Hospital, Wenzhou Medical University, Wenzhou, 325027 Zhejiang China; 3 State Key Laboratory of Genetic Engineering, School of Life Sciences, Fudan University, Shanghai, 200433 China

**Keywords:** Neuroscience, Stem cells

## Abstract

Pathogenic mutant huntingtin (mHTT) infiltrates the adult Huntington’s disease (HD) brain and impairs fetal corticogenesis. However, most HD animal models rarely recapitulate neuroanatomical alterations in adult HD and developing brains. Thus, the human cortical organoid (hCO) is an alternative approach to decode mHTT pathogenesis precisely during human corticogenesis. Here, we replicated the altered corticogenesis in the HD fetal brain using HD patient-derived hCOs. Our HD-hCOs had pathological phenotypes, including deficient junctional complexes in the neural tubes, delayed postmitotic neuronal maturation, dysregulated fate specification of cortical neuron subtypes, and abnormalities in early HD subcortical projections during corticogenesis, revealing a causal link between impaired progenitor cells and chaotic cortical neuronal layering in the HD brain. We identified novel long, oriented, and enriched polyQ assemblies of HTTs that hold large flat Golgi stacks and scaffold clathrin+ vesicles in the neural tubes of hCOs. Flat Golgi stacks conjugated polyQ assemblies by ADP-ribosylation factor 1 (ARF1). Inhibiting ARF1 activation with Brefeldin A (BFA) disassociated polyQ assemblies from Golgi. PolyQ assembles with mHTT scaffolded fewer ARF1 and formed shorter polyQ assembles with fewer and shorter Golgi and clathrin vesicles in neural tubes of HD-hCOs compared with those in hCOs. Inhibiting the activation of ARF1 by BFA in healthy hCOs replicated impaired junctional complexes in the neural tubes. Together, endogenous polyQ assemblies with mHTT reduced the Golgi recruiting ARF1 in the neuroepithelium, impaired the Golgi structure and activities, and altered the corticogenesis in HD-hCO.

## Introduction

Huntington’s disease (HD) is a devastating neurodegenerative disease with mid-adulthood onset [1] that is caused by a dominantly inherited CAG expansion in the *huntingtin (HTT)* gene [2]. Emerging in vitro and in vivo evidence has revealed neurodevelopmental impairments in fetuses and children with HD, and neurodevelopmental abnormalities extend into multiple temporal [3–6] and spatial dimensions [7–12]. Histologically, neurodevelopmental abnormalities occur during the period from neural progenitor cells to mature neurons [5, 6]. Anatomically, they impair multiple brain regions, such as through the thinning of the cortex and atrophy of the striatum [11, 12]. Most research has focused predominantly on cross-sectional studies on the genetic context of HD patients [6]. A spatiotemporal model for characterizing neurodevelopmental impairments in HD will provide deeper insight into mHTT pathogenesis in the fetus.

HD is found only in humans and uniquely occurs in human brains that carry mHTT. The human cortex is significantly larger and more complex than the murine cortex. Thus, HD animal models rarely replicate higher anatomical and functional complexities observed in the human brain, especially cortical phenotypes [13–17]. Human brain organoids derived from human embryonic stem cell/induced pluripotent stem cells (hES/iPSCs) have demonstrated the potential to reveal human-specific neurodevelopmental abnormalities, such as autism [18], and previous studies have reported that unguided organoids derived from HD-iPSCs show impaired cortical fate differentiation [5, 19]. Thus, human brain organoids could serve as an alternative tool for characterizing HD-associated developmental defects within a human genetic context at cellular and anatomical levels.

In this study, we used HD-iPSCs and healthy sibling-iPSCs from a large HD family to create guided-cortical organoids, establishing a platform for characterizing HD disease phenotypes on both spatial and temporal scales and molecular levels. Throughout the developmental timeline, we identified pathological phenotypes from neural progenitor cells to mature neurons and their interactions with other brain regions. We also identified novel long, oriented, and enriched polyQ assemblies of HTTs conjugated with the Golgi stacks using ARF1. Incorporating mHTT into polyQ assemblies reduced Golgi recruiting ARF1 and created a shorter Golgi stack in the neuroepithelium. Our study revealed a Golgi-related pathogenic mechanism of mHTT in human corticogenesis.

## Results

### Deficient progenitor proliferation in HD-hCOs

**Fig. 1:**
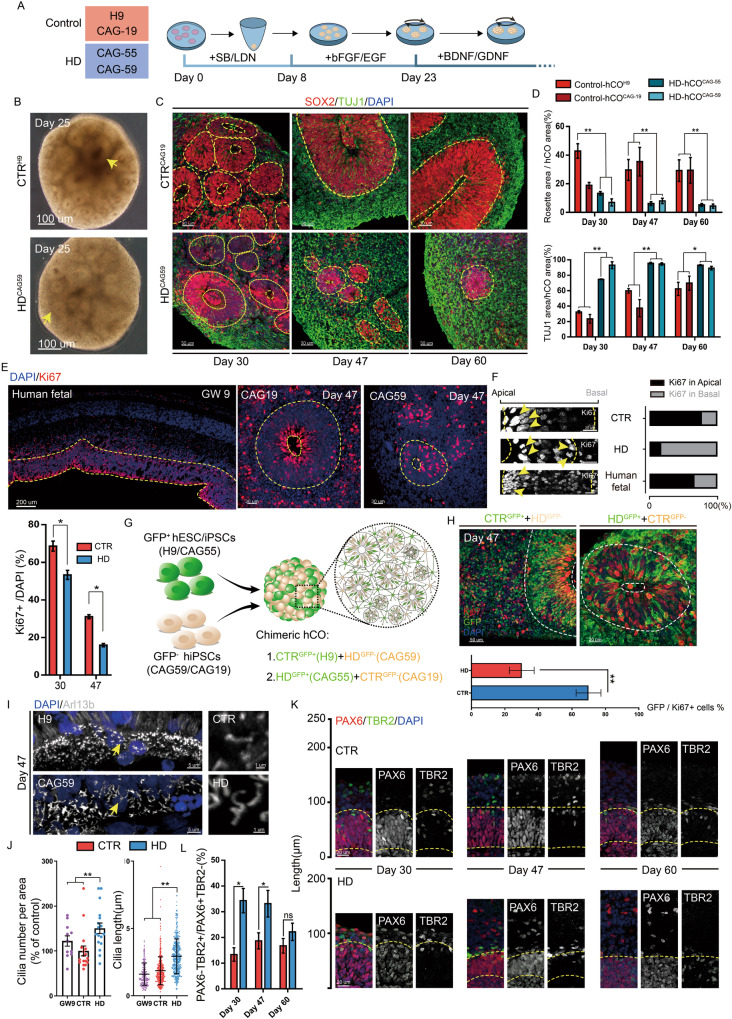
Impaired neural tubes present in HD hCO. **A** The schematic of the construction of hCOs and comparing strategy. CTR, control Group, including H9 and CAG 19; HD, Huntington’s Group, including CAG 55 and 59. **B** Representative bright-field images of CTR and HD hCOs on Day 25 showed multiple typical neural tube-like structure presented in hCOs (yellow arrows, neural tubes). **C** Immunostaining with TUJ1 and SOX2 antibodies outlined the neural tubes of hCOs on Day 30, 47 and 60 (yellow dashed lines, the borders of neural tubes in hCOs). **D** Comparing the ratio of neural tube (*n* = 6) and TUJ1+ coverage area (*n* = 6) of HD hCOs on Day 30, 47 and 60 with CTR (CAG 55 and 59 vs. H9 and CAG 19). Data, mean ± s.e.m. One-way ANOVA. *P < 0.05; **P < 0.01. **E** Representative images and counting of Ki67 (*n* = 6) antibody immunostaining in HD and CTR hCO (CAG 55 and 59 vs. H9 and CAG 19) and human fetal brain tissue (*n* = 1) (yellow dashed lines, the borders of neural tube). Comparing the ratio of Ki67+ (*n* = 6) cells on Day 30, 47 and 60 with CTR (CAG 55 and 59 vs. H9 and CAG 19). Data, mean ± s.e.m. One-way ANOVA. *P < 0.05. **F** Representative images and counting of Ki67 (*n* = 6) antibody immunostaining in the apical and basal side of CTR and HD hCO (CAG 55 and 59 vs. H9 and CAG 19) on Day 47 and human fetal brain tissue (*n* = 1) (yellow dashed lines, the borders of apical surface and basal surface; yellow arrows, typical Ki67+ cells). **G** Schematic of CTR/HD chimerism and chimeric rosette. **H** Representative images of Ki67 antibody immunostaining and counting Ki67+ cells (*n* = 5) in chimeric organoids (CAG 55 and 59 vs. H9 and CAG 19). Data, mean ± s.e.m. Student’s t-test. **P < 0.01. **I**–**J** Representative images of Arl13b antibody immunostaining (**I**) and calibrating Arl13b^+^ cilia length (*n* = 6) and density (*n* = 6) in the HD and CTR hCOs (CAG 55 and 59 vs. H9 and CAG 19) on Day 47(**J**). Data, mean ± s.e.m. Student’s t-test. **P < 0.01. **K** Immunostaining of PAX6 and TBR2 antibodies in HD and CTR hCOs on Day 30, 47 and 60 (dashed lines, boundaries of the VZ-like zones). **L** Comparing the percentage of PAX6-negative, TBR2-positive cells (PAX6^+^/TBR2^+^) over PAX6-positive, TBR2-negative (PAX6^+^/TBR2^-^) progenitors (*n* = 6) in HD and CTR hCOs (CAG 55 and 59 vs. H9 and CAG 19). Data, mean ± s.e.m. One-way ANOVA. *P < 0.05; ns, nonsignificant.

To study early corticogenesis in HD patients, we used human iPSC lines derived from the dermal fibroblasts of two HD patients (CAG 55 and 59) to generate HD-hCOs (Fig. 1A). Healthy hCOs were derived from hiPSCs (CAG 19; healthy sibling) and the embryonic stem cell line H9 was used as a control (CTR) (Fig. 1A). We performed a rigorous growth trajectory analysis of hCOs for 60 days (Fig. S1A). The growth curve showed that CTR-hCOs expanded faster than HD-hCOs after approximately 20 days (Fig. S1A). Notably, we observed that the neural rosettes in the CTR group were larger and richer than those in the HD group (Fig. 1B, S1B).

Brain organoids, a model of human brain development in vitro, comprise a population of neuroepithelial cells (NEs) assembled into large neural rosettes during the early period, representing the origin of neurogenesis [15, 20]. Staining with the apical complex (AC) markers N-cadherin (NCAD), β-catenin, and ZO-1 revealed the formation of junctional complexes in the apical domain (Fig. S1C), demonstrating apicobasal polarity in the neuroepithelium of the rosettes. To investigate HD neurogenesis, we selected organoids on days 30, 47, and 60, and stained the neural tube with antibodies against SOX2, an early neuroepithelial marker, and TUJ1, a pan neural marker (Fig. 1C, S2A). Calibration of the immunostaining results showed that the thickness of the neural tube and coverage of the neural tube-like area in HD-hCOs matched observations in the bright field (Fig. 1D, S2B). The increase in the TUJ1^+^ neuronal area paralleled the decrease of the neural tube in both the HD and CTR groups, whereas the increase in the CTR group was slower (Fig. 1D).

The balance between proliferation and differentiation of progenitors is a crucial determinant of neural tube size during development [21]. Therefore, we evaluated proliferative NEs using Ki67 and phospho-histone 3 (PH3) antibody immunolabeling (Fig. 1E, S2C). A sharp decline in mitotic progenitors in the HD group compared to the CTR group was observed from day 30 to 47 (Fig. 1E, S2D). TUNEL staining showed that the neural tubes in early HD cortical organoids did not contain more apoptotic cells than those in the CTR group (Fig. S2E). This excluded the possibility that the increase in apoptosis in progenitor cells caused a thinner neural tube in HD-hCOs. Remarkably, more Ki67^+^ cells were present basally in the VZ zone of the HD-hCOs compared with the CTR group and healthy human fetal cortex (GW9) (Fig. 1F) To exclude variations between different organoids, we constructed two chimeric hCOs that contained a uniform mixture (1:1) of HD and CTR cells, and one line (HD or CTR) was stably transfected with green fluorescent gene (*GFP*) (Fig. 1G). During monitoring, a large neural tube containing both GFP^+^ and unlabeled cells was observed (Fig. S3A), and histology further revealed that GFP^+^ cells were uniformly mixed with the untagged cells (Fig. S3B). However, the HD cells tended to be excluded from the VZ-like area (Fig. S3C). The GFP^+^ fluorescence intensity indicated that HD cells tended to be distributed basally compared to CTR cells in the VZ (Fig. S3D and S3E). Consistent with the phenotype of single hCOs, the proportion of HD Ki67^+^ neuroepithelial cells in the same neural tube was lower (Fig. 1H) and tended to be basally distributed (Fig. S3F). These findings indicate that early exhaustion of progenitors might be a common phenomenon in HD-hCOs, contributing to the thinner cortex in the fetal and childhood brain that carry mHTT.

### Premature neurogenesis in HD-hCOs

Defects in neural progenitor differentiation and neurogenesis biased toward the neuronal lineage have been observed in the human HD fetal brain at 13 gestational weeks (GW) [6]. We investigated whether HD-hCOs model premature neurogenesis in HD fetuses. Arl13b antibody staining, a marker for cilia, showed that Arl13b-positive cilia were present in the apical domain of the neuroepithelium in cortical organoids, resembling cilia in healthy human fetuses (GW9) (Fig. S3G). Apical cilia in the neural tube of the HD group were longer and denser than those in the CTR group and normal human fetal brains (Fig. 1I, J), reflecting less mitotic activity in the neural tube in HD-hCOs. To determine whether the reduction in mitosis disrupts the status of the apical progenitor (AP) and basal progenitor (BP) populations in HD-hCOs, we stained hCOs with antibodies against the AP marker PAX6 and BP marker TBR2 (Fig. 1K). The staining results showed that the ratios of TBR2^+^PAX6^-^/TBR2^-^PAX6^+^ in the HD group were higher than those in the CTR group (Fig. 1L), indicating that early lineage specification of the neural progenitors was preceded in HD-hCOs, which is consistent with the observations in HD fetuses and embryos of HD mice [6].

To understand the mechanisms underlying neurodevelopment in HD cortical organoids, we analyzed the global transcriptomes of the organoids on days 30 and 60. Differential gene expression analysis between the HD (55 or 59 CAGs) and control (19 CAGs, H9) groups identified 471 differentially expressed genes (DEGs) on day 30 and 1110 DEGs on day 60 (Fig. S4A). Principal component analysis showed a clear separation of HD and CTR hCOs on days 30 and 60 (Fig. S4B). The DEGs included several critical genes relevant to neurodevelopment (Fig. S4C): *HES3* [22], *NEUROD4* [23], *NEUROG1* [24], *NKX6.1/2* [25], and *WNT5A* [25], typical neurogenesis regulators, were upregulated on day 30, whereas *FEZF2* [26], which regulates the fate of subcortical projection neurons, was downregulated. Notably, several genes showed opposite differential trends at the two time points, including *FAM107A* [27] and *GSX2* [28], and differences in the earliest transcription factors expressed in neuronal progenitors, including *DLX2/5* [29], *ARX* [30], and *ZNF536* [31], which are regulators of neuronal differentiation. The glutamate receptor-related genes *GRIK2* [32] and *GRM5* [33] and the synaptic transmission-related genes *CPLX1* [34], *NGEF* [35], *SYNGR1* [36], and *SYNPR* [37] were also upregulated on day 60 (Fig. S4C). To further investigate the divergent developmental trajectory between CTR and HD, we compared the similarity of transcriptomic profiles from HD and CTR organoids with published transcriptomic profiles from the human fetal brain in three different cortical regions and distinct developmental periods ranging from 8 post-conception week to 4 months (Fig. S4D). The results showed that on day 60, the highest correlated human brain development stage of HD-hCOs was later than that of the CTR group, similar to the pattern on day 30. Transcriptomics verified premature neurogenesis in HD-hCOs.

### Deficiency of cortical projection neurons and laminations in HD hCOs

**Fig. 2:**
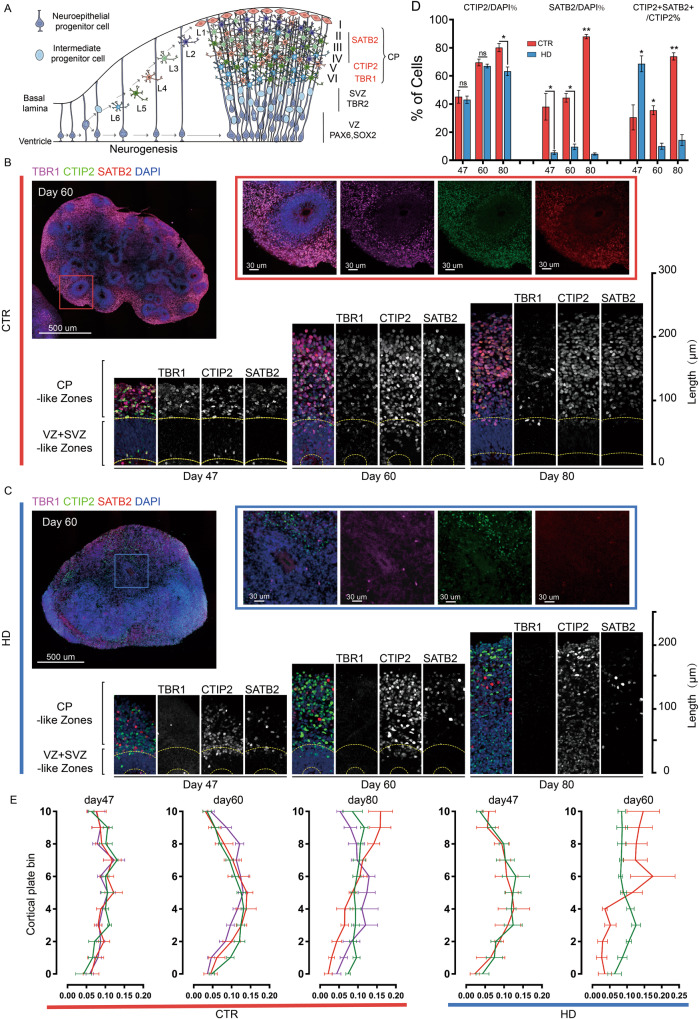
Perturbed cortical laminations and fate specification in cortical neuron subtypes in HD hCOs. **A** Schematic of the neuronal marker expression during cortical maturation and neural layering in healthy fetal brain (VZ, ventricular zone; SVZ, subventricular zone; CP, cortical plate). **B**, **C** Stitched and single channel images of TBR1, CTIP2 and SATB2 antibodies immunostaining in the different layers, including CP, VZ and SVZ regions, of CTR (**B**) and HD hCOs (**C**). The magnified images with single channel in the lower panel showed the gradient patterns of TBR1, CTIP2 and SATB2^+^ cells in the different layers of hCOs (yellow dashed lines, the margins of the VZ and SVZ-like area). The VZ-like area on Day 80 HD hCOs are chaotic (not indicate by a dashed line). **D** Comparing TBR1^+^, CTIP2^+^ or SATB2^+^ cells in HD hCOs with CTR (*n* = 8) on Day 47, 60 and 80 (CAG 55 and 59 vs. H9 and CAG 19). Data, mean ± s.e.m. One-way ANOVA. *P < 0.05; **P < 0.01; ns, nonsignificant. **E** Comparing the distributing tendency of TBR1^+^, CTIP2^+^ and SATB2^+^ neurons (*n* = 8) in the CP-like of hCOs of HD group with CTR (CAG 55 and 59 vs. H9 and CAG 19). The CP-like is evenly divided into 10 bins, following the apical-to-basal direction. Curves representing the normalized abundance within each bin. The value, marked cells in a bin / total neurons of CP-like. Data, mean ± s.e.m.

From the beginning of neurogenesis, newborn neurons radially migrate toward the pia surface, populating the cortical plate (CP) that constitutes the different layers in a nested “inside-out” progression from the deeper cortex to the more superficial cortex [38, 39] (Fig. 2A). To determine whether premature neurogenesis and the deficiency of proliferative progenitors alter the structure of the CP-like region in hCOs, we used TBR1, CTIP2, and SATB2 antibodies, representing the projection neurons in the different cortical layers, to stratify the cortical layers [26, 40–42]. TBR1^+^, CTIP2^+^, and SATB2^+^ cells covered most regions outside the VZ in CTR-hCOs, outlining the CP-like regions in hCOs and mimicking the layering of three projection neurons in the human fetal brain (Figs. 2B, S5A and S5B). In contrast, TBR1^+^, CTIP2^+^, and SATB2 ^+^ cells in HD-hCO were scattered in the CP-like regions of HD-hCOs (Figs. 2C, S5C). The cell count results of hCOs on days 47, 60, and 80 showed that the ratio of TBR1^+^, CTIP2^+^, and SATB2^+^ cells in the HD group was lower than that in the CTR group, and the differences in SATB2^+^ and TBR1^+^ cells were striking (there were too few TBR1^+^ cells in the HD group to count) (Fig. 2D).

Based on another protocol [43], we divided the thickness of the CP into 10 evenly spaced bins to evaluate the laminar patterns of TBR1^+^, CTIP2^+^, and SATB2^+^ cells. In the CTR group, TBR1 and SATB2 exhibited two separate peaks, representing the upper and deep layers, on day 80, and CTIP2^+^ cells spanned the entire CP (Fig. 2B, E), indicating layer-specific marker expression in the human neocortex [41]. In contrast, TBR1^+^ cells were barely observed in HD-hCOs on days 47, 60, and 80, and SATB2^+^ cells displayed more mutually exclusive domains with CTIP2^+^ cells in the deep layers on day 60 (Fig. 2C, E) than that in CTR group. Assessment of laminar expression patterns in the HD group was challenging on day 80 because of neural tube exhaustion, and SATB2^+^ cells were scattered in the region of CTIP2^+^ cells (Fig. 2C, S6A). Collectively, these results revealed that the decrease in cortical projection neurons in the HD group was accompanied by the perturbation of neuronal layering.

Similar to the segregation of CTIP2 and SATB2, including the increase in CTIP2^+^ and CTIP2^+^/SATB2^+^ cells, SATB2^+^ deep neurons continuously increased in CTR-hCOs, suggesting the post-mitotic fate specification of existing cortical neurons during the establishment of separated laminar expression domains (Fig. 2B, D). These observations are reminiscent of the cortical development of newborn excitatory neurons that co-express the projection specification transcription factors SATB2 and CTIP2, and these markers then segregate entirely by a narrow topographical transition [43, 44]. Neither SATB2^+^ cells in HD-hCOs increased from 47 to 80 days, nor did SATB2^+^/CTIP2^+^ cells increase compared to CTRs (Fig. 2D). The accumulation of CTIP2^+^ cells and the decrease in CTIP2^+^/SATB2^+^ cells in the HD group were dramatic compared to those in the CTR group (Fig. 2C–E). Together, these findings imply that the aberrant CP-like regions in the HD group may be due to the dysregulated fate specification of specific cortical neuron subtypes. The capacity of HD progenitors to differentiate into CTIP2^+^ and SATB2^+^ neurons was identical to that of CTRs in the chimeric organoids (Fig. S6B–S6D). However, the decrease in colabelled HD cells revealed impaired fate determination of neuronal subtypes in chimeric organoids (Fig. S6D).

### Delayed maturation of postmitotic neurons may contribute to the abnormal accumulation of CTIP2 in HD-hCOs

To determine whether HD neuronal progenitors also entered the mature stage, we used SOX2 and NEUN, mature neuron markers, to stain CTR and HD-hCOs on days 60 and 80. SOX2^+^ cells were restricted to the VZ-like zones, whereas NEUN^+^ cells covered the remaining areas in the CTR group. Massive SOX2^+^ cells ectopically appeared in the outer region of the VZ in HD-hCOs (Fig. S7A). NEUN^+^ cells were rarer in HD-hCOs than in CTR-hCOs. The increase in SOX2^+^ cells and decrease in NEUN^+^ cells in the outer region of the VZ-like zones in the HD group differed from those in the CTR group (Fig. S7B). Notably, the number of ectopic SOX2^+^ cells in the outer region of the VZ-like zones decreased in a time-dependent manner in HD-hCOs from days 60 to 80. At the same time, the ratio of NEUN^+^ mature neurons also decreased (Fig. S7A), indicating that the two phenotypes coexist in HD-hCOs, and some that diverged from the progenitor identity were retained in a stagnant stage before differentiating into mature neurons. In HD-hCOs, a population of cells that do not express SOX2 or NEUN strongly supported the incomplete or delayed maturation of these progenitors.

**Fig. 3:**
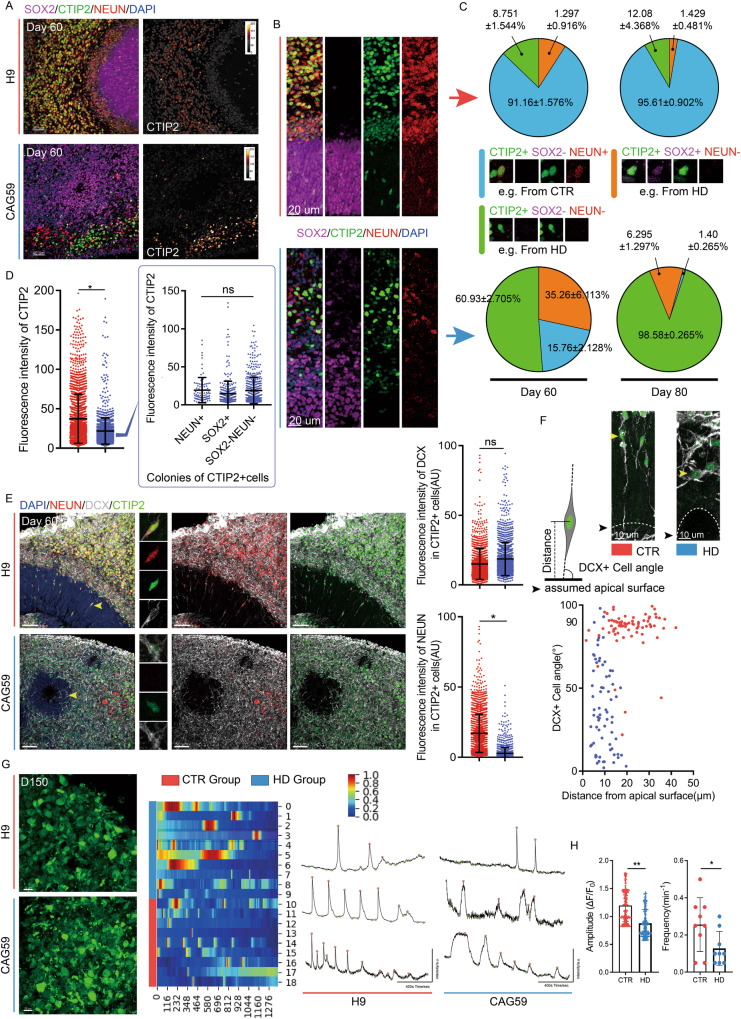
Delayed maturation of postmitotic neurons in HD hCOs. **A** Representative images of immunostaining with SOX2, CTIP2 and NEUN antibodies in HD and CTR hCOs on Day 60. The left panel is CTIP2^+^ cells in hCOs (displayed as gradient grey value). **B**, **C** Representative images (**B**) showing the distribution tendency of CTIP2^+^SOX2^-^NEUN^+^ / CTIP2^+^SOX2^+^NEUN^-^/CTIP2^+^SOX2^-^NEUN^-^ cells in the different layers of HD and CTR hCOs. Pie charts (**C**) showing the percentages of each type on CTIP2^+^ cells (*n* = 6) on Day 60 and 80. Data, mean ± s.e.m. **D** The graph of the intensity (*n* = 6) of CTIP2 antibody staining in three types of cells (CAG 55 and 59 vs. H9 and CAG 19) as described in **C**. Data, mean ± s.e.m. One-way ANOVA. *P < 0.05; ns, nonsignificant. **E** Immunostaining of NEUN, CTIP2 and DCX antibodies in HD and CTR hCOs on Day 60 (yellow arrows, typical DCX^+^ cells in VZ-like area). Comparing the CTIP2 immunostaining intensity (*n* = 8) of DCX/ NEUN in HD with CTR (CAG 55 and 59 vs. H9 and CAG 19). Data, mean ± s.e.m. One-way ANOVA. *P < 0.05; ns, nonsignificant. **F** The method of measuring DCX^+^ cell angle and the typical image for analysis. Scatter plots show the tendency of angle (*n* = 8) on Day 60 CTR/HD hCOs (CAG 55 and 59 vs. H9 and CAG 19). **G** Representative images of CTR/HD hCOs (CAG 55 and 59 vs. H9) stained with Ca2+ indicators for Ca2+ imaging on Day 150 (left panel). The heatmap showed the calcium traces normalized by the min-max (middle panel). Representative images indicate the calcium activity traces of individual neurons in CTR/HD hCOs (right panel). The red dots represent peaks, and the green represents bases. **H**. Quantifications of the average amplitude of ΔF/F per cell and calcium spike frequency of neurons from CTR/HD hCOs. Data, t-test. **P < 0.01.

Based on the exceptional accumulation of CTIP2^+^ cells and the dysregulated fate specification of specific cortical neuron subtypes in the HD group, we used CTIP2 antibody immunostaining, a specific neuronal layer marker, to characterize neurons with delayed maturation (Fig. 3A–D, S7B). The co-immunostaining of SOX2, NEUN, and CTIP2 antibodies showed that CTIP2^+^ cells nested around the VZ-like zones, and the expression levels of CTIP2 in neurons showed a gradient in the CTR group (Fig. 3D), similar to human fetal brain staining (Fig. S5A). CTIP2^+^ cells were scattered around the VZ-like zones, and the expression levels of CTIP2 in most cells were lower in the HD group than in the CTR group (Fig. 3B, C). In the chimeras, the fluorescence intensity of HD CTIP2^+^ cells was significantly lower than that of CTR cells in the CP region of the same neural tube (Fig. S8A, S8B).

In the CTR group, CTIP2^+^ cells highly overlapped with NEUN^+^ cells, whereas they were mutually exclusive within the SOX2^+^ regions (Fig. 3C). In contrast, all three markers displayed a scattered pattern in the HD group, and we identified many cells expressing CTIP2 but not other markers (Fig. 3C). These separate CTIP2^+^ cells appeared to lose the progenitor fate and were strangled at the intermediate stage. To track the destination of CTIP2^+^ cells in HD-hCOs, we identified CTIP2^+^ cells in both the CTR and HD groups. In hCOs from days 60 to 80, we found that there were three subpopulations of CTIP2^+^ cells: CTIP2^+^SOX2^-^NEUN^+^ cells represented mature CTIP2^+^ neurons; CTIP2^+^SOX2^+^NEUN^-^ cells included CTIP2^+^ cells with progenitor fate; and CTIP2^+^SOX2^-^NEUN^-^ cells. Only CTIP2^+^ cells were at an intermediate stage between the progenitor and mature cells (Fig. 3C, S7C). Generally, on days 60 and 80, significant CTIP2^+^ cell populations were CTIP2^+^SOX2^-^NEUN^+^ cells in the CTR group and CTIP2^+^SOX2^-^NEUN^-^ cells in the HD group (Fig. 3C). We also observed that CTIP2 and SOX2 expression were not mutually exclusive (Fig. 3B, C). CTIP2^+^SOX2^+^NEUN^-^ cells could be identified in both the CTR and HD groups. The percentage of CTIP2^+^SOX2^+^NEUN^-^ cells consistently decreased from days 60 to 80, revealing the equal potential of CTIP2^+^SOX2^+^ cells to leave the progenitor fate in both groups (Fig. 3E). The percentage of CTIP2^+^SOX2^-^NEUN^+^ cells in the CTR group increased from days 60 to 80 (Fig. 3C). However, the percentage of CTIP2^+^SOX2^-^NEUN^+^ cells decreased in the HD group, and the percentage of CTIP2^+^SOX2^-^NEUN^-^ cells increased significantly (Fig. 3C). Our results suggest that the exceptional accumulation of CTIP2^+^ cells in HD-hCOs may be due to delayed maturation. Moreover, all three HD group populations had the same levels of CTIP2 expression (Fig. 3D), indicating that CTIP2 expression is a consequence, but not the primary inducer, of delayed neural maturation.

To define the delayed maturation of CTIP2^+^ cells in HD-hCOs, we used doublecortin (DCX) antibody staining, a marker of immature and post-mitotic migrating neurons, to characterize their fate (Fig. 3E). Most DCX^+^ cells spanned the entire cortical plate, and a few were nested in the VZ-like zones (Fig. 3E). Almost all CTIP2^+^ cells expressed DCX, including NEUN^-^ cells in the HD group. The DCX expression level in CTIP2^+^ cells in the HD group was slightly higher than that in the CTR group; however, the expression level of NEUN decreased (Fig. 3E). Migrating DCX^+^ cells were present in the VZ-like zones of hCOs (Fig. 3E, F). To further investigate the possible cause of delayed maturation in DCX^+^ neurons, we quantified the migrating DCX^+^ cells by analyzing their positions in the substratum plane (Fig. 3F). In the VZ-like zone of the CTR group, the scattered DCX^+^CTIP2^+^NEUN^+^ cells were oriented perpendicular to the substratum plane (the angle between the cell axis and the assumed apical surface≈90°), and most migrating DCX^+^ neurons were 20 μm away from the apical surface, showing robust radial migration (Fig. 3F). In contrast, DCX^+^CTIP2^+^ scattered cells did not express NEUN in the VZ-like zone of HD-hCOs, corresponding to the location of DCX^+^CTIP2^+^NEUN^+^ cells in the CTR group (Fig. 3E). Meanwhile, the CTIP2^+^DCX^+^NEUN^-^ cell angle to the apical surface was more variable, and migrating neurons were within 20 μm from the assumed apical surface, similar to tumbling in place (Fig. 3F). Our analyses suggest that the delayed maturation of postmitotic neurons in HD- hCOs may result in the retention of CTIP2^+^ cells in the CP-like region, causing dysregulated fate specification of specific cortical neuron subtypes. To see the characteristics of cortical neuronal network in HD-hCOs, we performed calcium imaging with the fluorescence dyes (Cal-520 indicator) [45] in CTR/HD-hCOs cultured for 150 days and found that the frequency and the average amplitude of calcium activities in the neurons from HD-hCOs were significantly lower compared with those from CTR group (Fig. 3G, H). This result suggested that the altered corticogenesis in HD-hCO disrupts the cortical neuronal network of HD-hCOs.

### HD cortical projections aberrantly target striatal organoids early

**Fig. 4:**
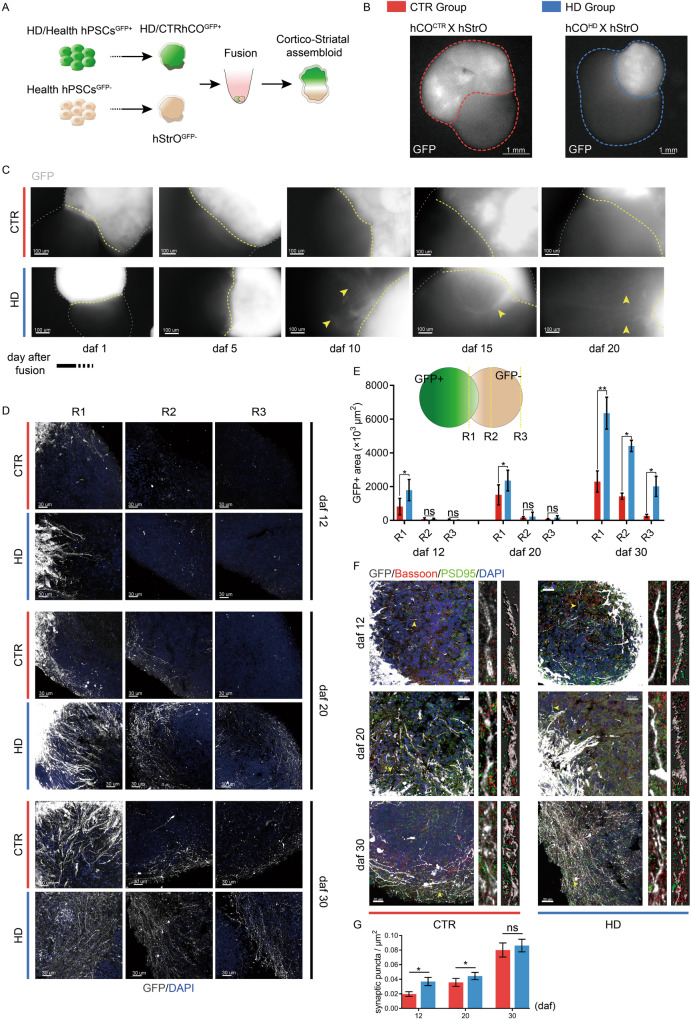
Formation of early projections from HD hCO in hC-StrO assembloids. **A**, **B** Schematic (**A**) of generating hC-StrO assembloids using GFP^+^ hCO and GFP^−^ hStrO and representative images (**B**) of hC-Stro assembloids. **C** Epifluorescence microscopy images showed the subcortical projections protruding from GFP^+^ hCO in hC-Stro assembloids (yellow arrows, GFP expressing projections). **D** Immunostaining revealed GFP-expressing projections in hC-StrO assembloids on Day 12, 20, and 30 (daf). The projections were immunostained by a GFP antibody. **E** The method of measuring GFP-expressing targeting projections in hC-Stro assembloids and comparing GFP^+^ projections area of HD and CTR hC-StrO assemblies on Day 12, 20, and 30 (daf). Data, mean ± SD. One-way ANOVA. *P < 0.05; **P < 0.01; ns, nonsignificant. **F** Immunostaining with Bassoon and PSD95 antibodies revealed the synapses on GFP-expressing projections in hC-StrO assembloids. Synapses are identified by the colocalization of presynaptic (red; Bassoon) with postsynaptic markers (green, PSD95). Neural surfaces were rendered, and PSD95^+^ puncta in a neuron were counted using Imaris 9.7 software (the top left panel). **G** Comparing the synapses on GFP expressing projections of HD with CTR of hC-StrO assembloids. The overlapped signal of Bassoon with PSD95 is defined as a synapse. Data, means ± s.e.m. One-way ANOVA. *P < 0.05; ns, nonsignificant.

Subcortical projections were constructed using an assembloid model to determine whether delayed maturation disrupts subcortical projections. The human cerebral cortex establishes projections toward the striatum [46]. We fused CTR or HD-hCOs tagged with GFP and healthy human striatal organoids (hStrOs) to create hC-Stro assembloids (Fig. 4A). Upon fusion of hCO with hStrOs for 30 days, we observed projection-like fibers in hCO^HD^-hStrO that aggregated into bundles protruding from the fluorescent margin of HD-hCOs and merged into hStrO after 5 days after fusion (daf) (Fig. S8C). Notably, the GFP^+^ positive spindle originated from the margin of the proximal fusion site, traversed the striatum, reached the distal part, and expanded as fusion proceeded (Fig. 4B, C). However, we did not observe any apparent projection/spindle protruding from a cortical organoid derived from the CTR group during the monitoring period (Fig. 4C). We further sectioned and stained the fused organoids to characterize the subcortical projections on 12, 20, and 30 daf. Based on the method of measuring the fused organoid [47], we calibrated the area of the GFP^+^ mass in a normalized region (see Methods) (Figs. 4D, E, S9A). The data showed that the volume of the GFP^+^ region (S _R1/2/3_) in hCO^HD^ -hStro was higher than that in hCO^CTR^-hStro at all three time points (Fig. 4E). Most GFP^+^MAP2^+^ neurons expressed the vesicular glutamate transporter (vGLUT1), indicating their glutamatergic identity in the assembloids (Fig. S9B).

Synapse formation is the hallmark of neuronal maturation [48]. To determine whether the projections of HD-hCOs were mature, we used Bassoon, a presynaptic marker, and PSD95, a postsynaptic marker, to stain GFP^+^ projections across striatal organoids. The Bassoon^+^ puncta paralleled the PSD95^+^ puncta on projections in 3D rendered images (Fig. 4F, G), and the synaptic puncta count on HD-hCO projections was higher than that on CTR-hCO projections on 12 and 20 daf and reached the same level on 30 daf (Fig. 4G). These data suggest that HD-hCOs form mature subcortical projections earlier than CTR-hCOs.

### PolyQ assemblies with mHTT lead to deficient Golgi apparatus, clathrin+ vesicles and the junctional complex in neuroepithelial cells of hCOs

**Fig. 5:**
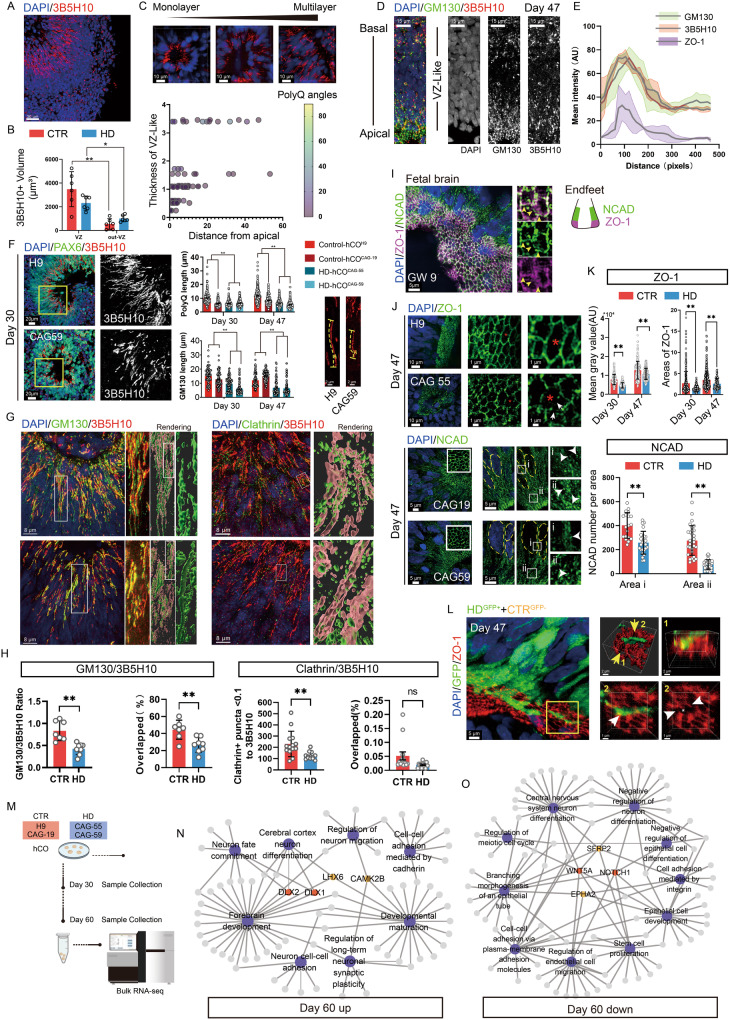
PolyQ assemblies with mHTT lead to deficient Golgi apparatus and the junctional complex. **A** Representative images of immunostaining of 3B5H10 antibodies in hCOs on Day 47. **B** Comparing of the volume of 3B5H10+ polyQ assemblies located VZ and out-VZ. Data, means ± s.e.m. One-way ANOVA. *P < 0.05, **P < 0. 01; ns, nonsignificant. **C** Representative images of immunostaining of 3B5H10 antibodies in neural tubes of different sizes. The scatterplot is a generalization to (*n* = 8) neural tubes, the horizontal axis is distance of origin of 3B5H10^+^ polyQ assemblies from apical, vertical axis is area of neural tube, pseudocolor is the angle of 3B5H10^+^ polyQ assemblies compared to the angle of the long axis of the NEs. **D-E** Representative images (**D**) of immunostaining of 3B5H10 antibodies in neural tubes on Day 30. The statistical plot (**E**) is the mean intensity of GM130, 3B5H10 and ZO-1 in concentric circles at different distances from the lumen of the neural tube. Neural tubes reveal consistent tendencies of GM130, 3B5H10 and ZO-1 protein localization in the VZ-like area. Data, means ± SD. **F** Representative images of immunostaining of 3B5H10 antibodies in hCOs on Day 30. Comparing the length of 3B5H10+ assemblies (*n* = 10) and GM130+ assemblies (*n* = 10) in neural tube of HD hCOs with CTR (CAG 55 and 59 vs. H9 and CAG 19). The right panel images with single channel showed the structure of 3B5H10^+^ PolyQ assemblies in the neuroepithelium (yellow dashed lines, the length and width of the 3B5H10 assemblies). Data, means ± s.e.m. One-way ANOVA. *P < 0.05. **G** Representative images of 3B5H10, GM130 and Clathrin antibodies immunostaining images and their sectional views in VZ-like area revealed the spatial relationships between polyQ assemblies, Golgi apparatus and Clathrin-coated vesicles (Taken by SIM microscopy). **H** Measuring the overlapped volume GM130^+^ Golgi with 3B5H10^+^ PolyQ assemblies (*n* = 3) and the overlapped volume of clathrin^+^ vesicles with 3B5H10 stained assemblies (*n* = 3) and the number of clathrin^+^ vesicles surrounded (within < 0.1 µm) the 3B5H10 stained assemblies (*n* = 3) in neural tubes of HD and CTR hCOs (CAG 47, 55 and 59 vs. H9 and CAG 19). Data, Students’ *t*-test. **P < 0.01. **I** Immunostaining of ZO-1 and NCAD antibodies in human fetal brain (GW9). Schematic indicated the position of junctional complexes at the apical endfeet. **J** Immunostaining of ZO-1 and NCAD antibody in hCO on Day 47. **K** Quantification of the area (*n* = 6) and mean gray value (*n* = 6) of ZO-1 at the junction between two adjacent cells (white arrows, the lower signal belt of ZO-1 staining) (CAG 55 and 59 vs. H9 and CAG 19). Quantifications of the density (*n* = 6) of NCAD staining in areas i and ii (CAG 55 and 59 vs. H9 and CAG 19). The arrow points to the typical spot-like NCAD structure. Data, mean ± SD. One-way ANOVA. **P < 0.01. **L** Representative images of GFP and ZO-1 staining in chimera on Day 47 and magnified view of ZO-1 structure surrounding a single GFP expressing protrusion (white arrows, the lower signal belt of ZO-1 staining). **M** Schematic diagram of Bulk RNA-seq. **N-O** GO biological processes related with neurodevelopment and cell adhesion. The purple dots represent pathways and other dots represent genes.

*CAG* expansion in patients with HD encodes a pathogenic mHTT with expanded polyQ, which is prone to aggregate [49]. To test the relationship between mHTT and aberrant corticogenesis in HD-hCOs, we used the 3B5H10 antibody, which is a polyQ antibody that identifies both polyQ in HTT and mHTT, to characterize the polyQ of HTT or mHTT in hCOs (Fig. 5A). Compared to the discontinuous fragments of HTT in the VZ region of the human fetal brain that we observed and others have reported [6], polyQ of HTTs preferentially exhibited as a long and continuous assembly, paralleling the orientation of NEs in the VZ-like zone of healthy and HD hCOs (Fig. 5B−E). The NEs of the VZ region contain more 3B5H10 stained polyQ assemblies than other regions, including the CP (Fig. 5B, E). Long and continuous 3B5H10^+^ polyQ assemblies were also conjugated with HAP40, a stabilizer of HTT, and co-labeled with HTT antibodies (C-terminal and 3E10) (Fig. S10A and S10B). In addition, the location, orientation, and size of the polyQ assemblies depended on changes in NE in the VZ; the larger the neural tube, the longer and richer the polyQ assemblies (Fig. 5C). To our knowledge, the polyQ assemblies highly resemble the orientation of the Golgi apparatus in the NEs of the VZ [50]. By characterizing the polarity of polyQ assemblies in the VZ-like zone from apical to basal, we found that the peak of polyQ signals approximated the apical domain, resembling GM130^+^ Golgi and apical complex (Fig. 5D, S1C). In Pax6+ cortical progenitors, polyQ assemblies in the HD VZ-like zone were shorter than those in the CTR group (Fig. 5F). Similar to polyQ assemblies, Golgi stacks in the HD VZ-like zone were shorter than those in the CTR group (Fig. 5F, the plot graph of the left lower panel). These observations indicate that the expanded polyQ of mHTT may alter the Golgi apparatus and trafficking in the neural tube during corticogenesis. SIM scanning images with nearly 64 nm resolution showed that 3B5H10^+^ polyQ assemblies scaffolded GM130^+^ Golgi and clathrin+ vesicles and included straight flat Golgi stacks in their inner grooves in NEs (Fig. 5G). Strikingly, polyQ assemblies with mHTT in the neural tube scaffolded fewer Golgi and clathrin^+^ vesicles than those without mHTT (Fig. 5H).

The junctional complexes are disrupted in the neural tube of the HD fetal brain, especially the tight junctions (TJ) [6]. To observe the alterations of junctional complexes in HD-hCOs, we used ZO-1, TJ antibody, and NCAD, adherent junctional (AJ) antibody, to characterize junctional complexes in HD-hCOs. Using ultrahigh-resolution scanning by SIM, we found that ZO-1 and NCAD exhibited an apparent hexagonal structure on the apical domain of the neural tube in hCOs, which is nearly identical to the TJs and AJs in the VZ of the human fetal brain (Fig. 5I-K). ZO-1-stained hexagonal reticular structures were partially blurred or irregular/discontinuous in HD-hCOs compared to the continuous and well-organized structure of TJs in healthy hCOs or fetuses (Fig. 5K, S10C). Counting of NCAD^+^ puncta in the neural tubes revealed fewer AJs in HD-hCOs (Fig. 5K, S10D). In addition, chimeric hCO data demonstrated that blurred TJs’ and discontinuous patterns were unique characteristics of scattered or patched HD progenitors, but not of healthy progenitors (Fig. 5L, S10E). Clathrin+ vesicles regulate TJs in epithelial cells, and the impairment of junctional complexes causes forced detachment of the apical endfeet and induces precocious neurogenesis [51]. Taken together, the impairment of junctional complexes related to the expanded polyQ of mHTT may cause aberrant corticogenesis in HD-hCOs by dismantling Golgi-related activities.

### Gene Ontology (GO) networks link aberrant neurodevelopmental and cell adhesion in HD-hCOs

To enhance our global view of HD corticogenesis, we performed GO functional enrichment analysis based on hCO bulk RNA sequencing (Fig. 5M). The results showed that the top 20 highly enriched biological processes were related to neurodevelopment (Table S1), including regionalization, regulation of neurogenesis, glial cell proliferation, and pattern specification process. In addition, GO analysis revealed that the enrichment of DEGs in HD-hCOs was particularly related to cell adhesion, cilium movement, and extracellular matrix terms, such as cell‒cell adhesion mediated by cadherin and cilium organization. We also performed GO analysis using the same parameters to examine two previously published HD human cortex transcriptional profiling datasets (GSE79666 61 and GSE64810 62) (Fig. S11A and S11B). We found some shared GO terms such as pattern specification, neuron fate commitment, extracellular structure organization, and regulation of adhesion. To further explore the link between the two biological processes and the prevalent simultaneous alteration of neurodevelopment and cell adhesion, we constructed GO networks using functional terms related to these two biological processes (Figs. 5N and 5O, S11C). The results showed that as hCOs developed, the number of intersections between the genes of the GO term increased. Wnt signaling plays a key role in neurodevelopment and neuronal maturation, and regulates morphogenesis by controlling cell adhesion and migration65. Hub genes common to both neurodevelopment-and cell adhesion-related pathways at both time points were members of the WNT family, such as WNT5A and WNT3A. Transcriptomic analysis confirmed the altered cell adhesion in HD- hCOs, supporting its role in aberrant neural development.

### Endogenous mHTT reduced the binding of ARF1 to Golgi-polyQ assemblies complexes in the neural tubes of hCOs

**Fig. 6:**
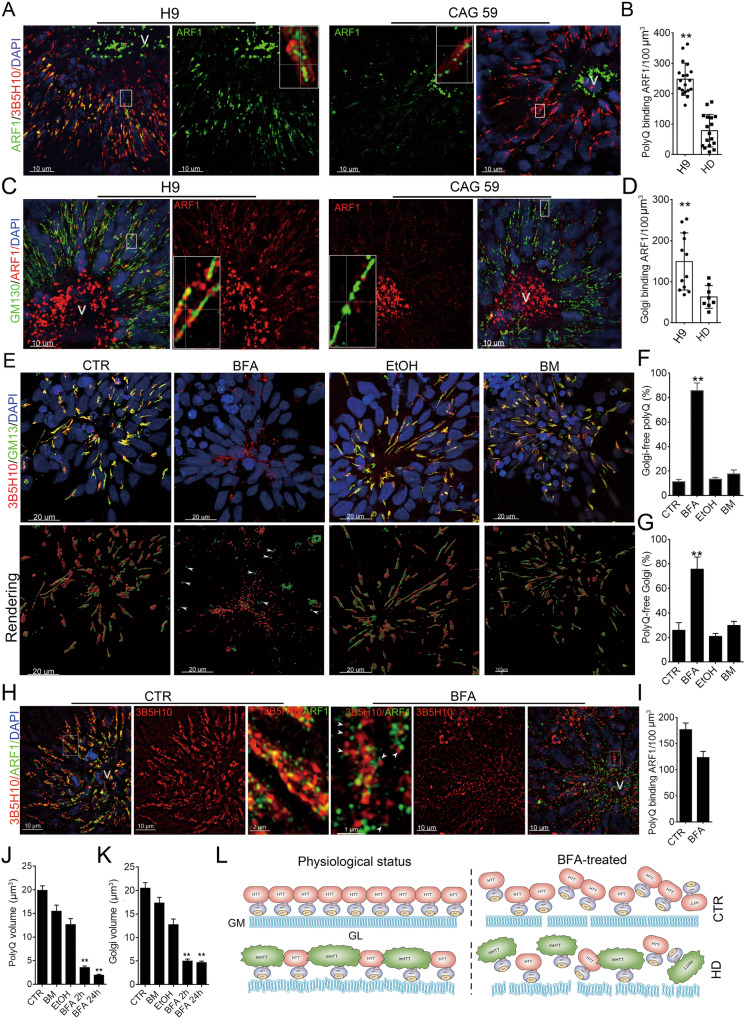
ARF1 mediates the binding of HTTs to Golgi stacks, and mHTT reduced Golgi stacks recruiting ARF1. **A** The immunostaining of 3B5H10 and ARF1 antibodies in the neural tubes of HD-hCO (CAG 59) and CTR-hCO (H9). The middle panels showed the immunostaining signals of ARF1 antibody (green). The inner inserts in the middle panel are a sectional view image that reveals the relationship between polyQ assemblies and ARF1 (white arrows, ARF1^+^ puncta). Images were taken by SIM and displayed with 3D projection. v, ventricle. **B** Comparing the counts of polyQ assemblies attached ARF1^+^ puncta in HD-hCO (CAG 59) with CTR-hCO (H9). ARF1 counting was performed by IMARIS spots, and polyQ volume was measured by IMARIS surface. Data, means ± SD; t-test, **p < 0.001. SIM images for analysis, *n* > 20. **C** The immunostaining of GM130 and ARF1 antibodies in the neural tubes of HD-hCO (CAG 59) and CTR-hCO (H9). The middle panel showed the immunostaining signals of ARF1 antibody (red). The inner inserts in the middle panel are a sectional view image revealing the relationship of ARF1^+^ puncta with Golgi (white arrows, ARF1^+^ puncta). v, ventricle. **D** Comparing the count of Golgi attached ARF1 puncta in HD-hCO (CAG 59) with CTR-hCO (H9). ARF1 counting was performed using IMARIS spots, and Golgi volume was measured using the IMARIS surface. Data, means ± SD. *t*-test. **p < 0.001. SIM images for analysis, *n* ≥ 8. **E** The immunostaining of 3B5H10 and GM130 antibodies in the neural tubes of CTR-hCOs (H9) treated by BFA, EtOH and BM for 2 h. The lower panels were the rendering images of 3B5H10 and GM130 staining by IMARIS surface (white arrows, polyQ-free Golgi). **F**,**G** Comparing Golgi-free polyQ assemblies and polyQ-free Golgi in the NEs of CTR-hCOs (H9) treated by BFA, EtOH, and BM. The distances between two objects were measured by IMARIS distance after rendering the surface of signals. Data, mean ± s.e.m. One-way ANOVA. **p < 0.001. SIM image for analysis, *n* = 8, **H** The immunostaining of 3B5H10 and ARF1 antibodies in the neural tubes of CTR-hCO (H9) treated by BFA for 2 h revealed that the fragmented polyQ puncta in BFA-treated hCOs were attached by ARF1 (white arrows, the fragmented polyQ with ARF1). The boxed regions are the magnified part in the inner inserts of the middle panels. v, ventricle. **I** The count of ARF1^+^ puncta attached to 3B5H10^+^ polyQ assemblies in BFA-treated NEs of hCOs and CTR-hCOs. The polyQ assemblies were measured by IMARIS surface, and IMARIS spots counted ARF1 puncta. The distance ≤0 µm is defined as a conjugate puncta. **J**,**K** Quantification of the volume of 3B5H10^+^ polyQ assemblies and GM130^+^ Golgi in CTR-hCOs (H9) treated by BFA, EtOH and BM. Data, mean ± s.e.m. One-way ANOVA. **p < 0.001. Repeats, *n* = 3. **L** Graphical illustration of the relationships of HTTs with ARF1, ARFIP2 and Golgi. GM, Golgi membrane; GL, Golgi lumen.

The lower levels of clathrin+ and shorter Golgi in the neural tubes of HD-hCOs implied that polyQ assemblies with mHTT might dismantle a critical protein that regulates Golgi sorting and structure. PolyQ of HTTs binds ARFIP2, one of three known proteins that physically attach ADP-ribosylation factor (ARF1) [52]. ARF1, a guanosine triphosphatase (GTPase), recruits to Golgi after converting its GDP into GTP by guanine nucleotide-exchange factors (GEFs) and governs Golgi sorting activities, vesicle formation and Golgi structure [53, 54]. Thus, ARF1 might mediate the binding of polyQ assemblies of HTTs to long flat Golgi stacks via ARFIP2. The 3D SIM images of the immunostaining of 3B5H10 and ARF1 antibodies in hCOs revealed that ARF1 in NEs preferentially attached to the inner surface of long 3B5H10^+^ polyQ assemblies that face the Golgi stacks (Fig. 6A), supporting the binding of ARF1 to polyQ assemblies. The counting of 3B5H10^+^ polyQ assemblies binding ARF1 revealed that the polyQ assemblies with mHTT in the NEs of HD-hCOs attached fewer ARF1 puncta than that of polyQ assemblies without mHTT in CTR-hCOs (Fig. 6B). The immunostaining of GM130 and ARF1 antibodies in hCOs showed that the Golgi stacks of neural tube in HD-hCOs also recruited fewer ARF1 compared with that of healthy neural tubes in CTR-hCOs (Fig. 6C, D), suggesting that the incorporation mHTTs to polyQ assemblies reduced the recruiting of ARF1 to flat Golgi stacks. Brefelidin A (BFA) inhibits the recruiting of ARF1 to Golgi by inhibiting the Sec7 domains of GEFs, a catalytic domain that converts ARF1-GDP to ARF1-GTP [55]. BFA treatment, but not branaplam (BM), an inhibitor of *mHTT* gene transcription [56], and Ethyl Alcohol (EtOH), a Golgi fragmenting agent in long-term exposure [57], caused the disassociation of polyQ assemblies from the large Golgi stacks, and fragmented polyQ assemblies of HTTs and Golgi stacks (Fig. 6E−G). Consistent with the effects of BFA on ARF1, BFA treatments fragmented extended flat Glogi stacks and dramatically reduced Golgi recruiting ARF1 in the neural tubes (Fig. S12A−C). Notably, the SIM images of 3B5H10 and ARF1 antibodies’ immunostaining revealed many fragmented polyQ puncta/short assemblies in the neural tubes treated by BFA for 2 h conjugated with ARF1 (Fig. 6H, I). Strikingly, over 30% of smaller polyQ assemblies/puncta of HTTs were attached by ARF1 in the NEs of hCOs treated with BFA for 24 h (Fig. S12D, E). The data of polyQ assemblies and Golgi volume in the neural tubes showed that BFA treatments dramatically dwindled polyQ assemblies and Golgi volume compared with those in BM- and EtOH-treated neural tubes (Fig. 6J, K). The 3D SIM images of ZO-1 and NCAD antibodies’ immunostaining showed that BFA treatment for 24 h induced the structural collapse of TJs in the neural tubes but did not significantly reduced ZO-1 and NCAD expression (Fig. S12F). These findings support that the conjugation of polyQ assemblies of HTTs with Golgi stacks, which stabilize the long polyQ assemblies, depends on ARF1-GTP in the NEs, incorporation of mHTT to polyQ assemblies reduces Golgi recruiting ARF1 (Fig. 6L). Disrupting the binding of ARF1-GTP to Golgi replicates the impaired tight junctions in HD neural tubes.

## Discussion

Cortical malformations in pre-symptomatic HD children and fetuses suggest that the effects of pathogenic mHTT on the brain cover the entire life span [8, 58, 59]. Despite accumulating evidence indicating that mHTT impairs neuronal progenitors [5, 6], how mHTT affects human corticogenesis remains elusive. In this study, healthy and HD hCOs derived from a large HD family were used to characterize corticogenesis impairment in cellular and molecular levels. Our HD-hCO models exhibit deficient junctional complexes, premature neuronal differentiation, and delayed neuronal maturation, including the early formation of TBR2^+^ BPs, resulting in premature neuronal differentiation and the depletion of proliferation pools, replicating the alterations of corticogenesis in human fetus brain [6]. HD cortical progenitors differentiate into DCX^+^ post-mitotic neurons but do not immediately mature, forming disordered CP-like regions in HD hCOs.

We observed long, orientated, and enriched polyQ assemblies of HTTs in the neural tubes of hCOs, which include oriented Golgi stacks in their inner grooves. NEs have extended, flat, and polarized/oriented Golgi stacks involved with AP and dendrite polarity and radial glia migration in neurogenesis [60–62]. Thus, a large polyQ assembly of HTTs might be essential to build large, flat, orientated Golgi stacks in NEs, which regulate corticogenesis. Selective degradation of mHTT was considered a strategy to cure HD, especially mHTT aggregates [63]. Our findings revealed that the long assemblies of HTTs, including mHTTs, are a stable form of HTTs that resist degradation in the cell. The recruiting of HTTs to Golgi stacks and the formation of polyQ assemblies depend on the recruitment of ARF1 to the Golgi stacks. Of note, ARF1-dependent disassociation of polyQ assemblies from the Golgi disassembled and degraded HTTs, indicating that the polyQ assemblies of HTTs are fragile. Thus, endogenous polyQ assemblies of HTTs/mHTTs in NEs, which include flat Golgi, are physiological rather than pathological. The Golgi in the NEs of HD-hCO attached by fewer ARF1 compared with that in CTR-hCOs, suggesting the incorporation of mHTT to polyQ assemblies in NEs altered the structure of polyQ assemblies and dismantled ARF1 recruitments to Golgi. The lower levels of ARF1 recruitment to Golgi in NEs of HD-hCOs might be the leading cause of lower levels of clathrin^+^ vesicle and shorter Golgi. How endogenous mHTT disrupts the structure of polyQ assemblies and the recruitment of ARF1 to Golgi needs a deeper look in the future.

HTTs facilitate Golgi-related vesicular trafficking, and mHTTs impair vesicular trafficking [64, 65]. Junctional complexes attach to neighboring progenitors and seal the border of the neuroepithelium, which is critical for spatiotemporal differentiation in neurogenesis [51, 66]. Junctional complex proteins are synthesized on the ER, transferred to the cell membrane by clathrin+ vesicles after sorting in the Golgi apparatus, and form new junctional complexes, including TJ, AJ, and gap junctions [66]. The results of ARF1 inhibition in the neural tubes by BFA support the idea that the deficiency of ARF1 recruitment contributes to the junctional complex impairments in NEs of HD-hCOs, consequently altering the corticogenesis in the human fetal brain, which is regulated by a strict spatiotemporal manner [67].

Using HD-hCOs and hCO-hStrO assembloids, we recapitulated the altered corticogenesis and impaired junctional complexes in HD fetal brain. We identified long, oriented, polyQ assemblies of HTTs holding Golgi stacks in the neural tubes. PolyQ assemblies with mHTT reduced the recruiting of ARF1 to Golgi stacks of NEs in HD-hCOs. Inhibiting ARF1 recruitments to the Golgi leads to junctional complex impairments in NEs. Thus, restoring the recruitment of ARF1 to Golgi in HD neural tubes might rescue the altered corticogenesis in HD fetal brain.

## Materials and methods

### Human-induced pluripotent stem cell culture

Induced pluripotent stem cells (iPSCs) were reprogrammed from two HD patients’ skin fibroblasts and a health’s from one family. The iPSC and H9 human ES cell lines were cultured and maintained in feeder-free conditions using E8 medium at 37 °C with 5% CO_2_. The culture medium was changed daily, and the cells were passed on to a new plate precoated with Matrigel every six days by EDTA.

### Generation of human cortical organoids and striatal organoids

hCOs were generated as previously described [68]. Briefly, iPSCs were dissociated with Accutase (Gibco, CAT#: A1110501) and plated at a concentration of 9000 single iPSCs/well into three-dimensional embryoid bodies for one day using HNM medium (DMEM/F12; Gibco, CAT#: C11330500BT) with 1:100 N2 supplement (Gibco, CAT#: 17502-048), 1% (v/v) GlutaMAX supplemented (Gibco, CAT#: 35050-061), 1% (v/v) MEM-NEAA (Gibco, CAT#: 11140-050) and E8 at a ratio of 1:1 with the SMAD pathway inhibitors −0.5 mM SB-431542 (Ametek Scientific, CAT#: DM-0970), 0.1 mM LDN (STEMGENT, CAT#: 040074) and 10 µM Rock inhibitor Y27632 (APE. BIO, CAT#: A3008) in ultralow attachment 96-well plates (Corning; CAT#: 3474). On Day 2, EBs were transferred into 5 cm tissue culture plates in the same medium without Y27632. Day 8 spheroids were transferred to neural medium containing Neurobasal medium (Gibco, CAT#: 21103-049), 1% N2 supplement, 2% (v/v) B-27 supplement (Gibco, CAT#: 117504-044), 1% (v/v) GlutaMAX, 1% MEM-NEAA and 0.1 mM penicillin–streptomycin with basic fibroblast growth factor (bFGF, 20 ng*ml^−1^) and epidermal growth factor (EGF, 20 ng*ml^–1^). The hCOs were cultured for up to 30, 47, 60, or 80 d as indicated.

For human striatal organoids [68], Day 6 spheroids were maintained with HNM medium and E8 at a ratio of 1:1 with SB and LDN and transferred to neural medium with the sonic hedgehog (SHH) agonist 5 µM purmorphamine (Pur; APE. BIO, CAT#: A8228) on Day 10. Until the end of the differentiation process on Day 25, the medium was exchanged for neural medium and refreshed every 2–3 days.

### Generation of human cortico-striatal assembloids

To create assembloids [68], hCOs and hStrOs were derived separately and fused by placing them in position as close as possible in 1.5 ml Eppendorf tubes for 24 h in an incubator between Day 27 and 30 of differentiation. On Day 2, assembloids were transferred into 5 cm tissue culture plates in neural medium as described.

### Live Imaging

Real-time 2D live hCO imaging was recorded by an inverted phase contrast microscope with 1.25 X and 10X every two days after differentiation. Fiji (ImageJ, version 2.1.0, NIH) was used to calibrate the area of hCO derived from different cell lines. Consecutive serial images were obtained in hCO on the hStros side from Day 1 to Day 30 after fusion using the OLYMPUS IX73 fluorescence microscope with 1.25X, 10X and 20X objectives.

### Tissue preparation and immunostaining

For fixation, organoids were incubated in 4% paraformaldehyde (PFA) for 20 minutes at room temperature, followed by three washes with phosphate-buffered saline (PBS). The organoids were then incubated overnight in a 30% sucrose solution. Organoids were embedded in Tissue Frozen Medium (Leica), frozen in cold isopentane, and sectioned with a cryostat (Leica). For immunostaining, slides were permeabilized with 0.3% Triton-X and blocked in 10% donkey serum in PBS for 1 h. Primary antibodies were diluted in blocking solution and applied to the sections overnight. After washing with PBS-Tween (PBST), secondary antibodies, including DAPI, were diluted in blocking solution and applied to the sections for 1 h at room temperature. Finally, the sections were washed with PBST. See Supplementary Data (Tables S2 and S3) for details on immunostaining.

Leica SP8 Laser-Scanning Confocal Microscope and Nikon A1, Structured Illumination microscopy (SIM) (Nikon, Japan) were applied to scan all Z-stacked and stitched images. ImageJ software (Fiji, NIH, Bethesda, MD, USA) was used to count cells and perform morphological analyses. Imaris 9.8 (Bitplane AG, Zürich, Switzerland) was used for the particle size and rendering displays.

### Neural tube quantification

In hCO serial sections, the rosette lumen areas were quantified on Days 30, 47 and 60 of neural differentiation after staining for SOX2 and TUJ1. For rosette size, the contour of each rosette (as highlighted by markers SOX2 and TUJ1) and each lumen (as highlighted by marker SOX2) was outlined using ImageJ software, and two lists of regions of interest (ROIs) were measured to estimate rosette mean areas and VZ-like area mean thickness, respectively.

### GFP^+^ cells of chimerism quantification

After resuming the centrum of the neural tube lumen as the center of the circle, we drew the concentric circles along the direction of cell polarity by using the Radial Profile Plot (one plugin of ImageJ). The fluorescence intensity statistics of concentric rings were used as a measure of the distribution pattern of GFP cells in the VZ-like area.

### Quantification of GFP^+^ projections in hC-Stro assembloids

Based on Xiang’s [47] method, ImageJ calibrated the area of GFP^+^ mass in a standard region within 3 categories: approaching the fusion boundary (R1), already crossing the midline of the targeting organoid (R2), and approaching the opposite tip of the targeting organoid (R3).

### Live calcium imaging and analysis

Cal-520 calcium imaging of hCOs was performed as previously described [69]. hCOs were loaded with 1 μM Cal-520 (Abcam, ab171868) for 60 min at 37 °C in HHBS medium, and then incubate the plate at room temperature for a further 30 min. The Cal-520 dye working solution was replaced with HHBS. Calcium transients were recorded using a 40X objective on a Leica TCS SP8 confocal microscope and at a frame rate of 1.35 Hz with 512 × 512-pixel resolution. The signal extraction and downstream analysis were completed by platform-Mesmerize [70].

### 2D cortical neuron differentiation

Cortical organoids were dissociated into small clumps with Accutase at Day 30 and then transferred into new 5 cm tissue culture plates for 1 day. Finally placed these neurospheres onto ornithine/laminin-coated coverslips for culture and prepared for small molecules intervention.

### Ethyl Alcohol intervention

Anhydrous ethanol was supplemented to the cell culture media with a final concentration of 100 nM for 2 h.

### Branaplam intervention

Branaplam was reconstituted in DMSO with a concentration of 5 M. Branaplam was supplemented to the cell culture media with a final concentration of 10 nM for 2 h.

### Brefeldin A intervention

Brefeldin A was reconstituted in DMSO with a concentration of 1 M. Brefeldin A was supplemented to the cell culture media with a final concentration of 5 µg/ml for 2 h and 24 h.

### Living imaging of Golgi dynamics

For visualization of the Golgi apparatus in living cells, cardiomyocytes were loaded for 30 min with GOLGI ID® Green assay kit (ENZO, ENZ-51028-K100), a membrane-permeant Golgi apparatus marker. Following incubation, transfected cells were imaged live by microscopy.

### RNA extraction for RNA sequencing

Total RNA was extracted from the tissue using TRIzol® Reagent according the manufacturer’s instructions (Invitrogen), and genomic DNA was removed using DNase I (TaKaRa). Then, RNA quality was determined by a 2100 Bioanalyzer (Agilent) and quantified using an ND-2000 (NanoDrop Technologies). Only high-quality RNA samples (OD260/280 = 1.8 ~ 2.2, OD260/230 ≥ 2.0, RIN ≥ 6.5, 28 S:18 S ≥ 1.0, >1 μg) were used to construct a sequencing library.

### RNA sequencing

The RNA-seq transcriptome library was prepared following the TruSeqTM RNA Sample Preparation Kit from Illumina (San Diego, CA) using 1 μg of total RNA. Briefly, messenger RNA was isolated according to the polyA selection method by oligo(dT) beads and then fragmented by fragmentation buffer first. Second, double-stranded cDNA was synthesized using a Super Script double-stranded cDNA synthesis kit (Invitrogen, CA) with random hexamer primers (Illumina). Then, the synthesized cDNA was subjected to end repair, phosphorylation and ‘A’ base addition according to Illumina’s library construction protocol. Libraries were size selected for cDNA target fragments of 300 bp on 2% Low Range Ultra Agarose followed by PCR amplification using Phusion DNA polymerase (NEB) for 15 PCR cycles. After quantification by TBS380, the paired-end RNA-seq sequencing library was sequenced with the Illumina HiSeq xten/NovaSeq 6000 sequencer (2 × 150 bp read length).

### Differential expression analysis and functional enrichment

To distinguish the differentially expressed genes (DEGs) between HD and control groups, we first calculated the normalized expression level of each transcript from raw count data by the transcripts per million reads (TPM) method. Then, the differential expression analysis was performed by using the R package limma [71]. Genes with |log2FC | > 0.5 and P.adjust ≤ 0.05 were considered to be significant DEGs. Functional enrichment analysis of the Gene Ontology (GO) database including categories of biological process, molecular functions, and cell components was performed to identify the significant GO terms by the R package clusterProfiler [72]. The Benjamini-Hochberg FDR was used for multiple correction, and FDR ≤ 0.05 is considered to be significant. Subsequently, significant GO terms that were relative to the semantics such as neuron development and cell adhesion were selected to establish the pathway interaction network by using the Cytoscape platform [73].

## Supplementary information

Supplementary Material

## Data Availability

The sequencing datasets generated and/or analyzed during this study are available in the Gene Expression Omnibus with the following accession numbers for readers: GSE220847. Raw data are within the paper and its Supporting Information files: Tables S1.
