# Supplementary material for: Endogenous mutant Huntingtin alters the corticogenesis via lowering Golgi recruiting ARF1 in cortical organoid
Source: Mol Psychiatry. 2024 Apr 23;29(10):3024–39. doi: 10.1038/s41380-024-02562-0 (PMC11449793; doi:10.1038/s41380-024-02562-0)
Supplement: Supplementary file 1 — Supplementary Material [file 41380_2024_2562_MOESM1_ESM.pdf]

SUPPLEMENTARY FIGURES

Endogenous mutant Huntingtin alters the corticogenesis via lowering Golgi recruiting ARF1 in cortical organoid

Yang Liu[1#], Xinyu Chen[1#], Yunlong Ma[2#], Chenyun Song[1], Jixin Ma[1], Cheng Chen[2], Jianzhong Su[2], Lixiang Ma[1*] and Hexige Saiyin[3*]

[1]Department of Anatomy and Histology & Embryology, School of Basic Medical Sciences, Fudan University, Shanghai 200032, China

[2]Oujiang Laboratory (Zhejiang Lab for Regenerative Medicine, Vision and Brain Health), Eye Hospital, Wenzhou Medical University, Wenzhou, 325027, Zhejiang, China

[3]State Key Laboratory of Genetic Engineering, School of Life Sciences, Fudan University, Shanghai 200433, China

[#] the authors equally contributed to this study.

* Corresponding author. Email: lxma@fudan.edu.cn, saiyin@fudan.edu.cn

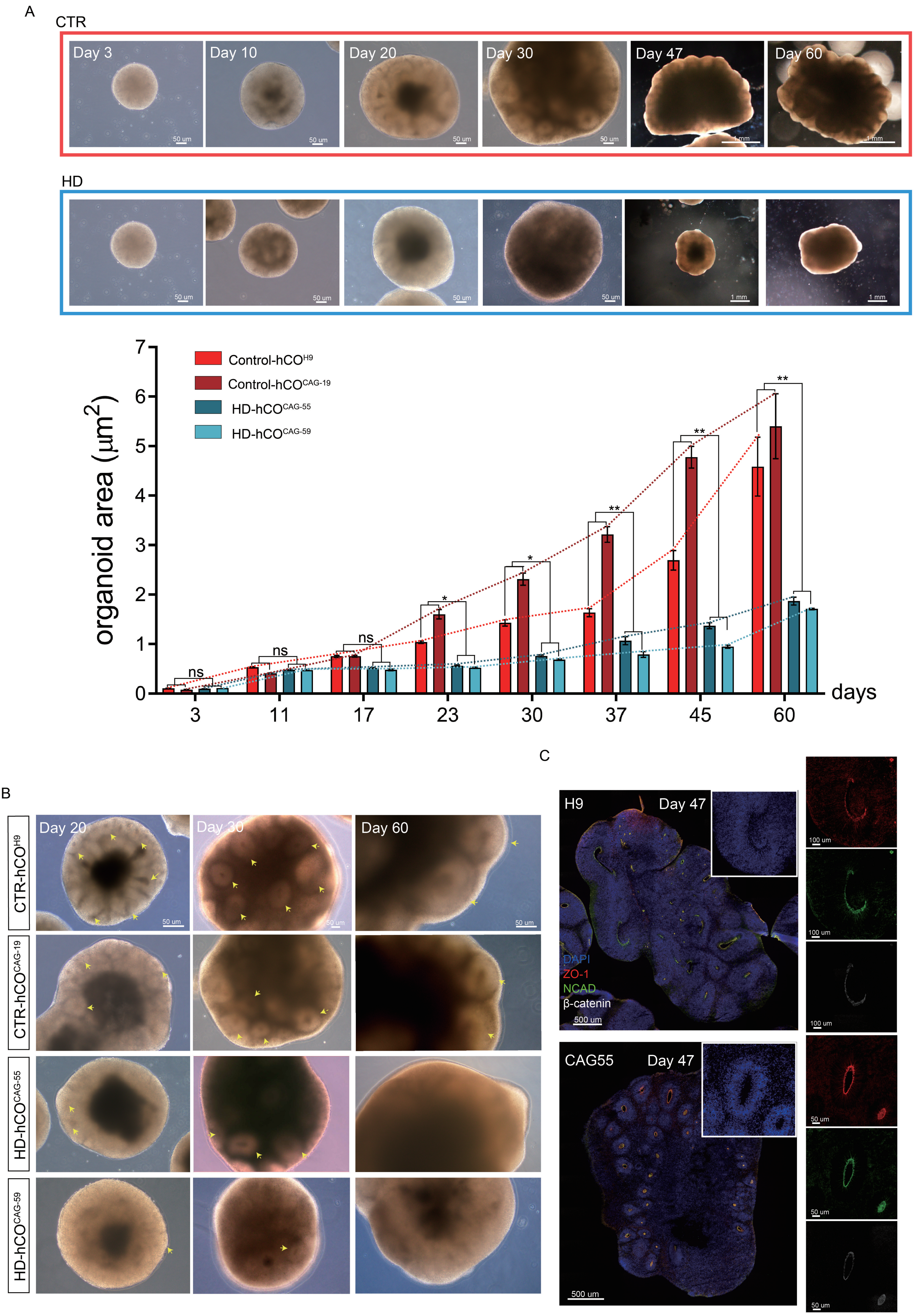

A
CTR
Day 3
Day 10
Day 20
Day 30
Day 47
Day 60
HD
Control-hCO^H9
Control-hCO^CAG-19
HD-hCO^CAG-55
HD-hCO^CAG-59
organoid area (µm^2)
days
ns
*
**
3
11
17
23
30
37
45
60
B
Day 20
Day 30
Day 60
CTR-hCO^H9
CTR-hCO^CAG-19
HD-hCO^CAG-55
HD-hCO^CAG-59
C
H9
Day 47
DAPI
ZO-1
NCAD
β-catenin
500 um
CAG55
Day 47

Supplementary Fig. 1. mHTT impaired the neuroepithelial structures in HD-hCO.

A. Representative bright-field images of hCOs during cultured and growth curves throughout differentiation (n=20) (CAG 55 and 59 vs. H9 and CAG 19). The largest cross-sectional area of each organoid was measured on the day listed in the figure. Data, mean ± s.e.m, One-way ANOVA. *$p < 0.05$, **$p < 0.01$.

B. Neural tube-like structural development in human cortical organoids.. Representative bright-field images captured at different stages of maturation.

C. The representative images of immunostaining with ZO-1, NCAD, and β-catenin antibodies in hCOs on Day 47 showed the junctional complexes in the apical domain of the neural tube, indicating the apicobasal polarity of the neuroepithelium.

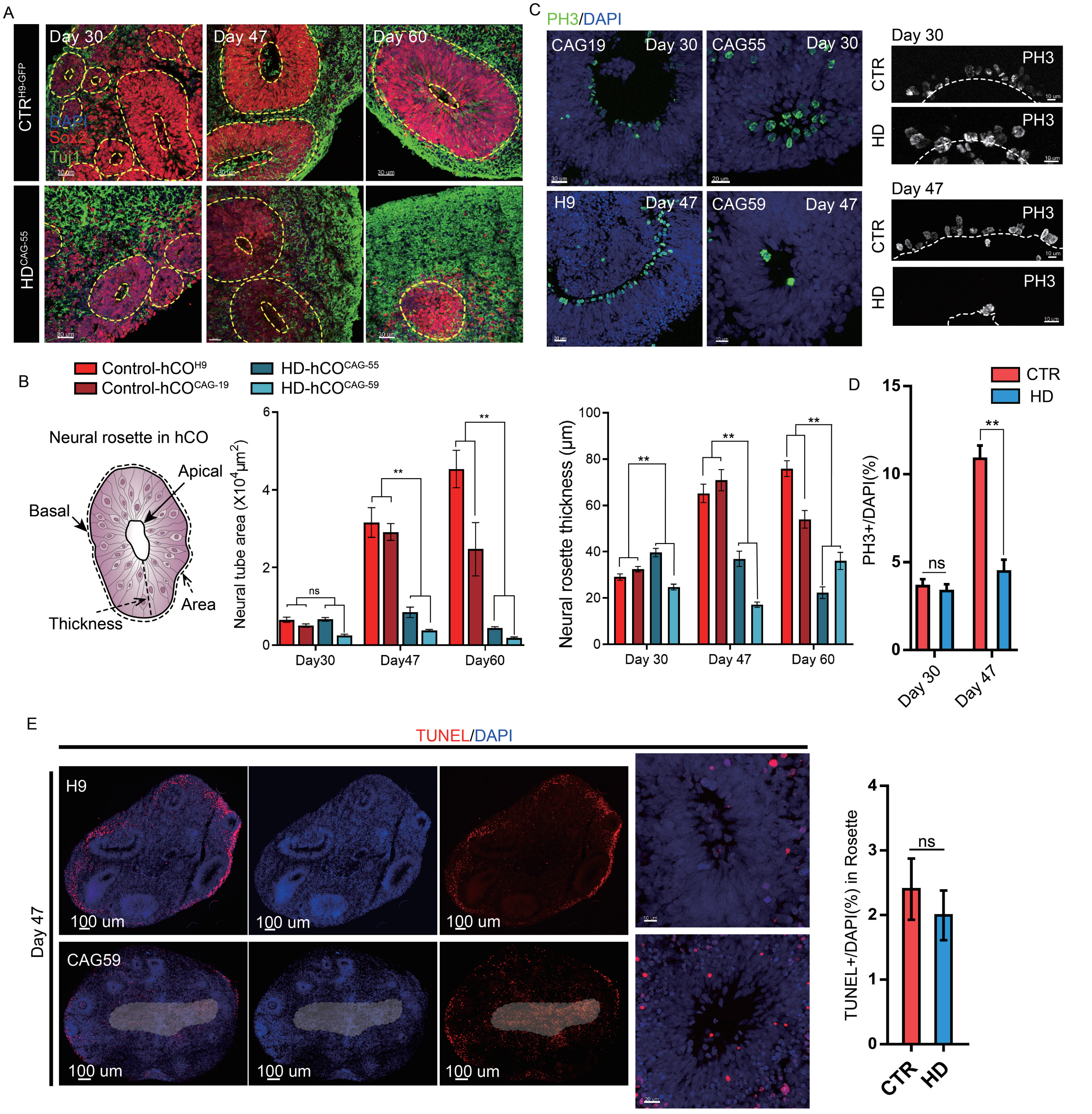

Supplementary Fig. 2. The neuroepithelial proliferation in HD hCOs were deficient.

A. Immunostaining with SOX2 and TUJ1 antibodies in HD hCOs and CTR hCOs on Day 30,47 and 60 (yellow arrows, neural tubes).

B. The method of calibrating the VZ-like area in hCOs. The minimum thickness of each VZ-like area was used to represent the neural tube depth. Comparing the area and thickness (n=6) of the neural rosette of HD hCOs on Day 30, 47, and 60 with CTR (CAG

55 and 59 vs. H9 and CAG 19). Data, mean ± s.e.m. One-way ANOVA. $^{**}P<0.01$.

C-D. Representative images (J) and counting (K) of pH3 (n=8) antibody immunostaining in hCOs (CAG 55 and 59 vs. H9 and CAG 19) on Day 30 and 47 (white dashed lines, the borders of neural tube lumen). Data, mean ± s.e.m. One-way ANOVA. $^{**}P < 0.01$; ns, nonsignificant.

E. TUNEL staining and comparing TUNEL^{+} cells in HD with CTR hCOs (CAG 55 and 59 vs. H9 and CAG 19) on Day 47. The grey area indicates the central necrotic area in the brain organoid. Data, mean ± s.e.m, Students' t test. ns, non-significant.

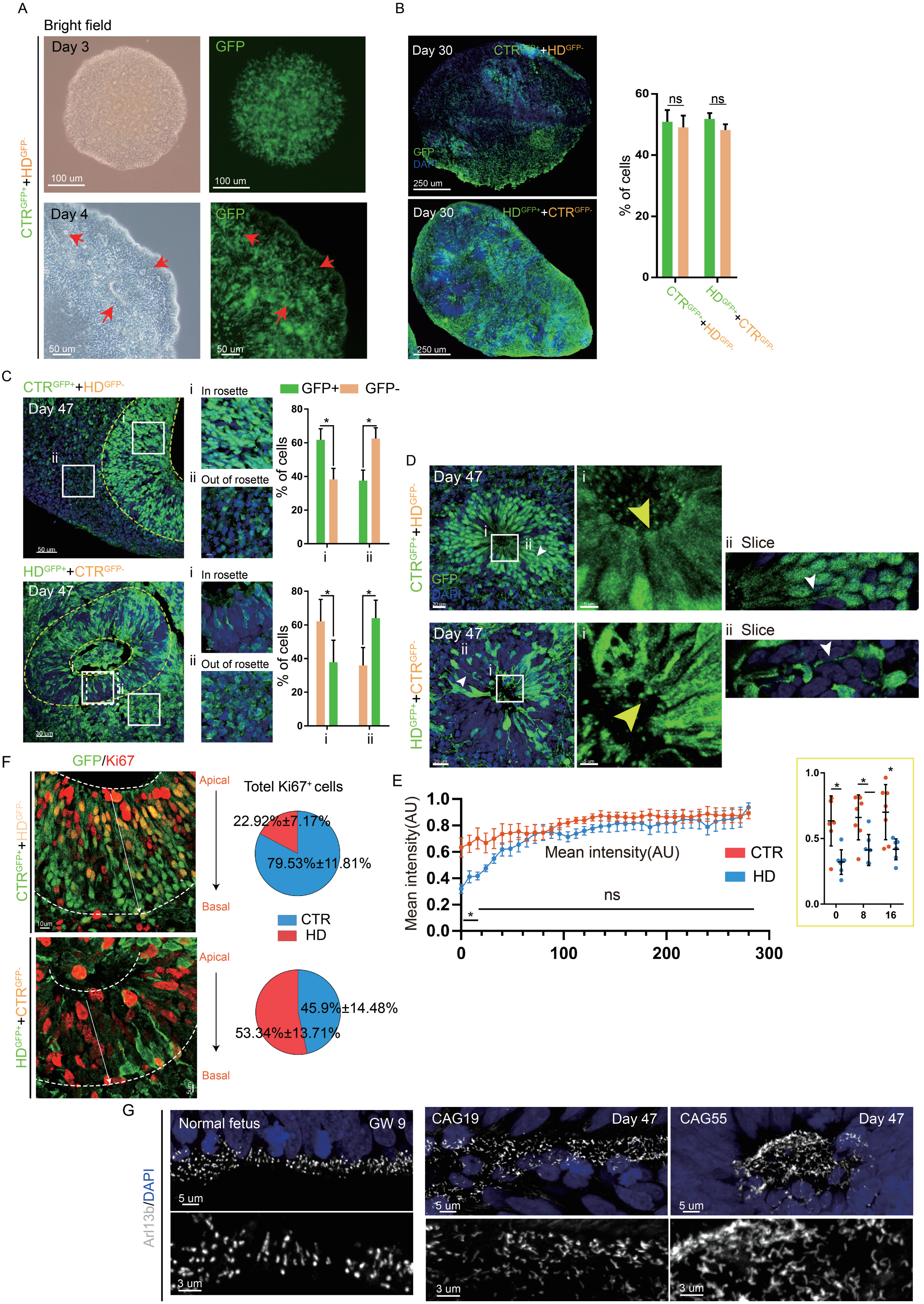
A
Bright field
Day 3
GFP
100 um
Day 4
50 um
CTRGFP++HDGFP-
B
Day 30
CTRGFP++HDGFP-
GFP
DAPI
250 um
HDGFP++CTRGFP-
60
40
20
0
ns
% of cells
C
Day 47
i In rosette
ii Out of rosette
GFP+
GFP-
80
% of cells
*
50 um
30 um
D
ii Slice
20 um
5 um
F
GFP/Ki67
Apical
Basal
10um
5um
Totel Ki67+ cells
22.92%±7.17%
79.53%±11.81%
CTR
HD
45.9%±14.48%
53.34%±13.71%
E
Mean intensity(AU)
1.0
0.8
0.6
0.4
0.2
0.0
0
100
200
300
ns
0.5
8
16
G
Normal fetus
GW 9
CAG19
Day 47
CAG55
5 um
3 um
Arl13b/DAPI

Supplementary Fig. 3. The preferences of progenitor distribution in chimeric HD-CTR hCOs.

A. Representative images of chimeric organoids on Day 3 and Day 4 (red arrows, neural tubes).

B. Representative images of chimeric organoids showed the HD cells uniformly intermingled with the CTR cells in chimeras. Counting GFP-expressing cells (n=5) in the whole hCO section (H9 vs. CAG59 and CAG55 vs. CAG 19). Data, mean ± SD. One-way ANOVA. ns, nonsignificant.

C. Representative GFP^+ cells in chimeras on Day 30 showed the different localization tendencies of HD and CTR cells in the whole hCO section. Counting GFP^+ cells (n=5) located inside (i) and outside (ii) of neural tube (H9 vs. CAG59 and CAG55 vs. CAG 19), separately. Data, mean ± SD. One-way ANOVA. *P<0.05.

D-E. Representative images of the GFP expressing cells in a VZ-like zone and high-magnification images of typical progenitor protrusion in section (I). The statistical plot (J) (n=5) is the mean intensity of GFP in concentric circles at different distances from the lumen of the neural tube. Chimeras reveal different tendencies of HD and CTR cell localization in the VZ-like area (white arrows, protruding single cell in two chimeric groups). Data, means ± SD. One-way ANOVA. *P<0.05.

F. Representative images (K) of Ki67 antibody immunostaining and counting $Ki67^+$ cells (L) (n=5) in chimeric organoids revealed the distribution patterns of $Ki67^+$ proliferating cells in the VZ-like zones (CAG 55 and 59 vs. H9 and CAG 19). Data, mean ± s.e.m. Student's t-test. **P < 0.01.

G. Immunostaining of Arl13b antibody in GW9 human brain and Day 47 hCOs sections.

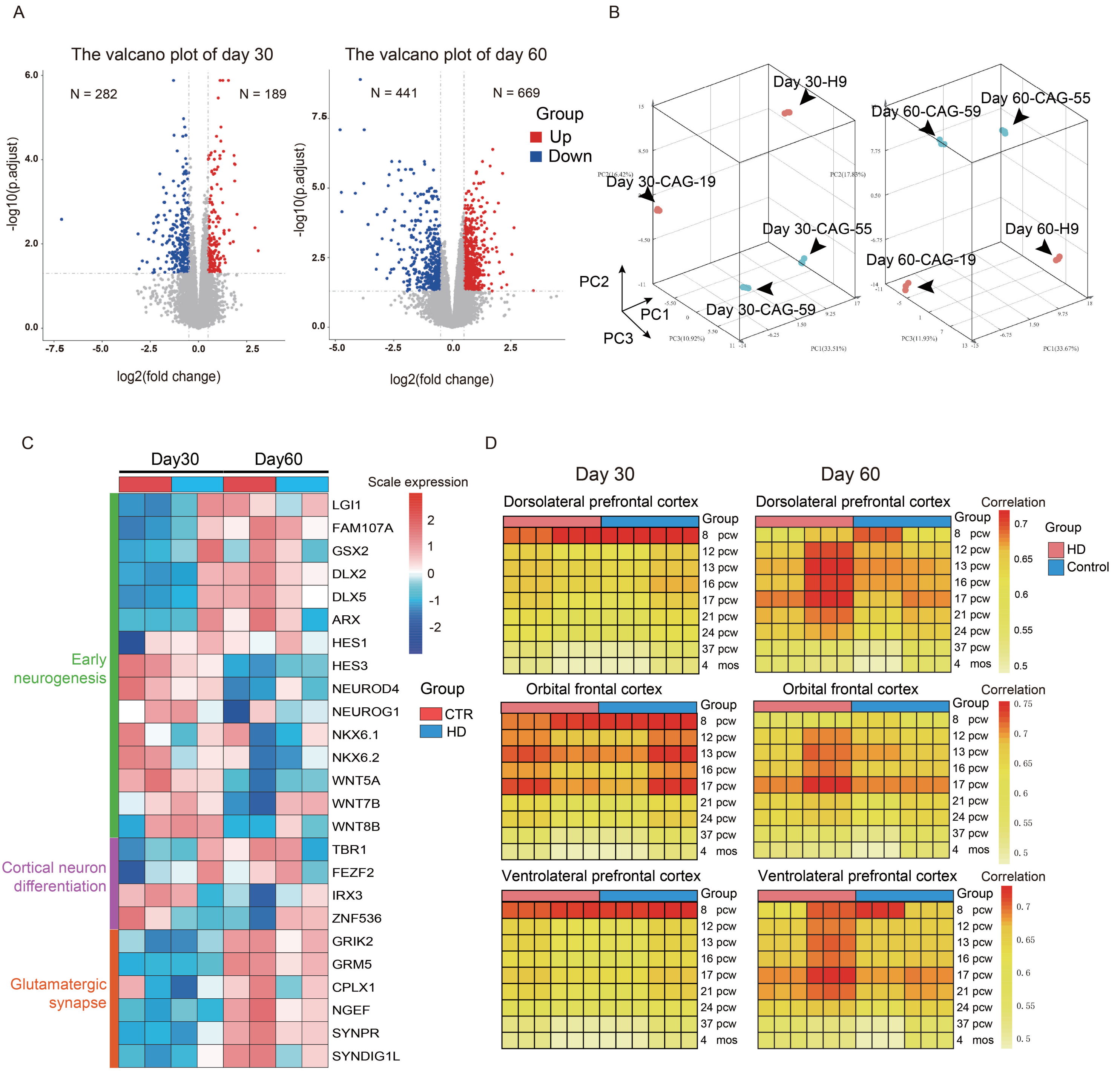

Supplementary Fig. 4. Transcriptome data revealed premature neurogenesis in HD hCOs

A. Volcano plot shows differentially expressed genes (DEGs) between CTR and HD hCOs on Day 30 and 60.

B. Principal component analysis (PCA) of CTR/HD hCO transcriptomes on Day 30 and 60.

C. Expression profile of key genes related to premature neurogenesis, Cortical neuron differentiation and Glutamatergic synapse.

D. Heatmaps of Pearson's correlation analysis comparing genes expressed in HD and CTR

hCOs (Day 30 and 60) gene expression profiles of human fetal brain (8 pcw-4 mos).

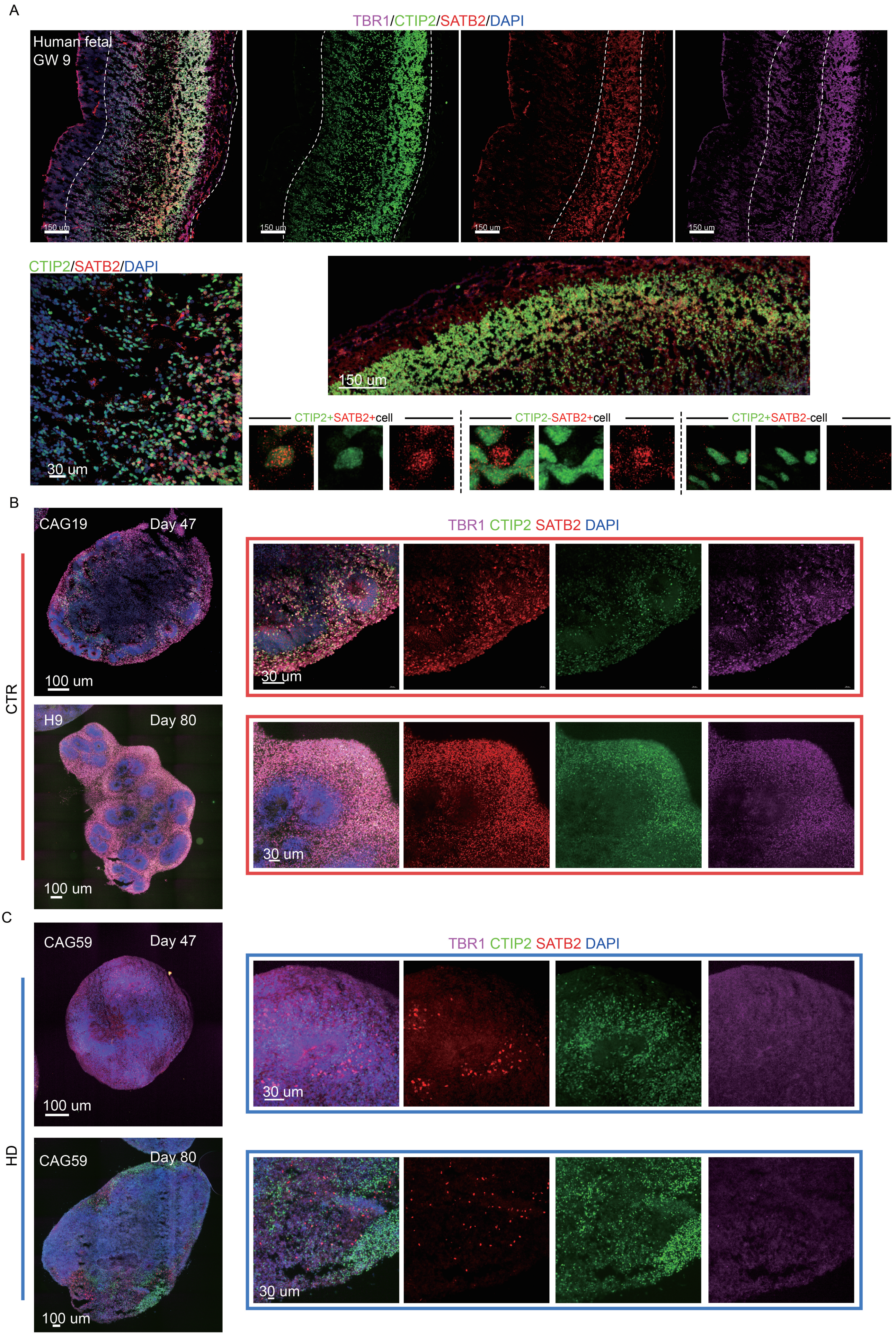
A
TBR1/CTIP2/SATB2/DAPI
Human fetal
GW 9
150 um
CTIP2/SATB2/DAPI
30 um
150 um
CTIP2+SATB2+cell
CTIP2-SATB2+cell
CTIP2+SATB2-cell
B
CAG19
Day 47
100 um
TBR1 CTIP2 SATB2 DAPI
30 um
CTR
H9
Day 80
100 um
30 um
C
CAG59
Day 47
100 um
TBR1 CTIP2 SATB2 DAPI
30 um
HD
CAG59
Day 80
100 um
30 um

Supplementary Fig. 5. HD-hCO exhibited an altered cortical plate.

A. Representative images of immunostaining with TBR1, CTIP2, and SATB2 antibodies in GW9 human fetal brain sections display the layering pattern in the fetal cortex.

B-C. Immunostaining of TBR1, CTIP2, and SATB2 antibodies in CTR and HD hCOs on Day 47 and 80.

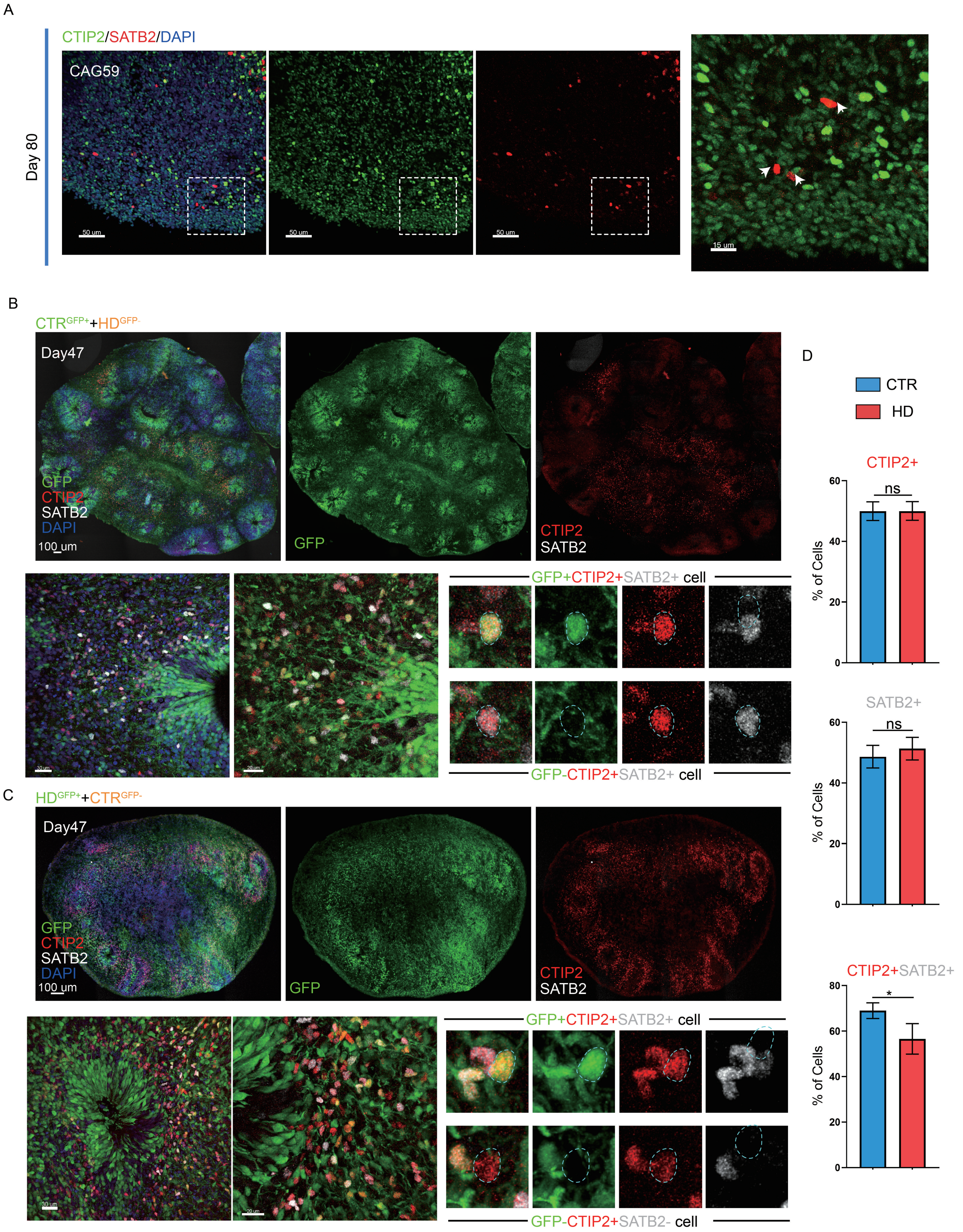

Supplementary Fig. 6. Differentiation patterns of CTIP2⁺ neuron and SATB2⁺ neuron in chimeras.

A. immunostaining showed SATB2$^+$ cells were scattered in CTIP2$^+$ cells in HD hCOs on Day 80.

B-D. Immunostaining (B and C) for GFP, CTIP2, and SATB2 antibodies and the comparison (D) of three populations (n=5) of neurons revealed the neural differentiation capacity of CTR/HD cells in chimeras. Data, mean ± s.e.m, One-way ANOVA. *$p < 0.05$, ns, non-significant.

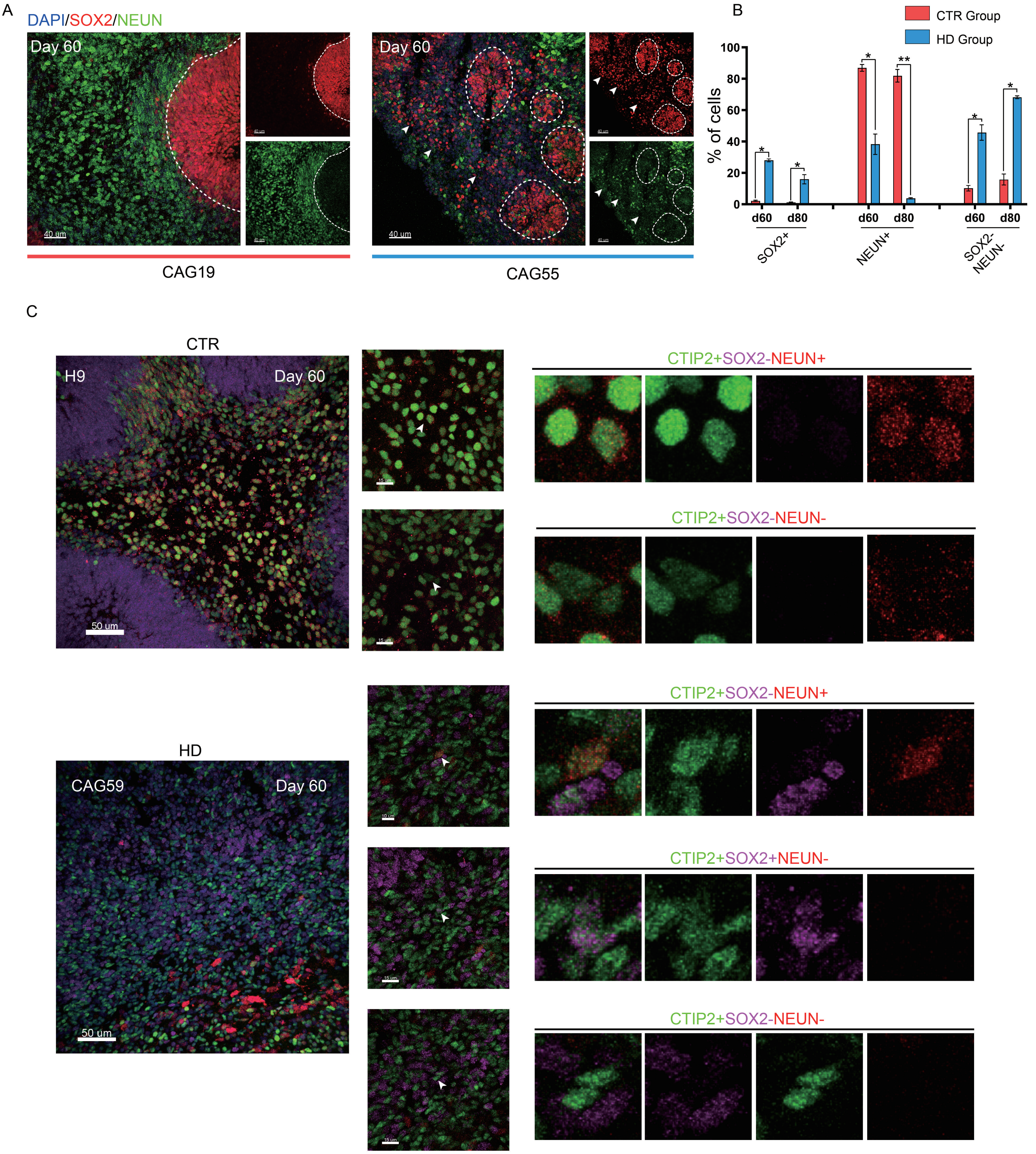

Supplementary Fig. 7. Defective cortical progenitor differentiation and maturation in HD hCO.

A. Representative images of SOX2 and NEUN antibodies' immunostaining in HD and CTR hCOs on Day 60 (dashed lines, the outer boundaries of the VZ-like area). The white arrows indicate the region where SOX2^{+} progenitors and sparse NEUN^{+} neurons present

in the HD group (outside of the VZ-like region).

B. Comparing the percentage of $SOX2^{+}$, $NEUN^{+}$, and $SOX2^{-}/NEUN^{-}$ cells (n=6) in the CP-like regions of HD hCOs with CTR hCOs (CAG 55 and 59 vs. H9 and CAG 19). Data, means ± s.e.m. One-way ANOVA. *$P < 0.05$; **$P < 0.01$.

C. Analysis of different $CTIP2^{+}$ cell types in the CP region of hCO on Day 60 revealed the presence of immature $CTIP2^{+}$ cells in HD hCO.

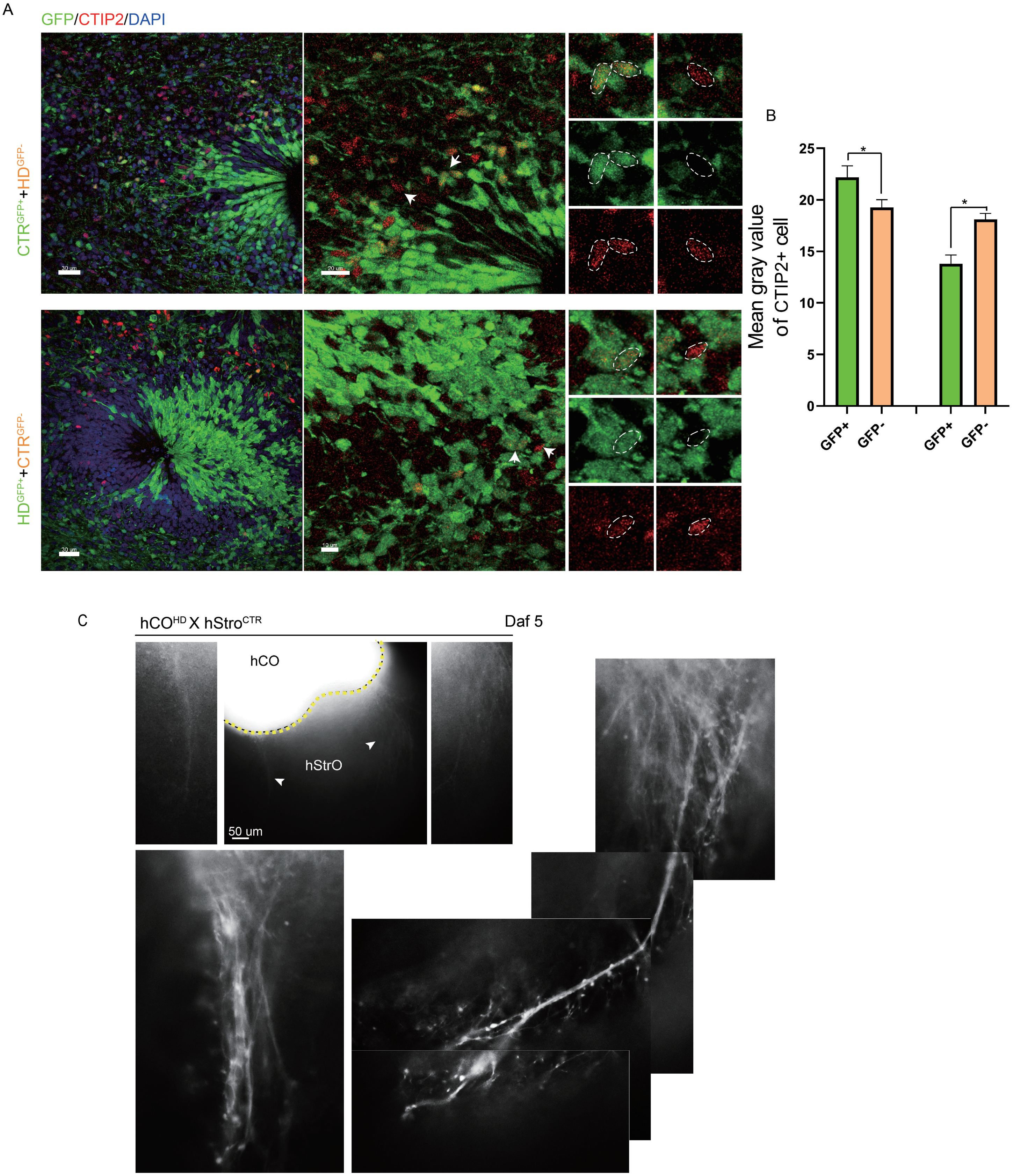

Supplementary Fig. 8. The levels of CTIP2 in HD CTIP2^{+} neurons of chimeras were weaker than that in CTR.

A-B. Immunostaining (A) of CTIP2 and GFP antibodies and comparing the mean gray value (B) (n=5) of CTIP2 in CTRGFP^{+}-HDGFP^{-} with CTR^{GFP-}HD^{GFP+} chimera. Data, means ±

s.e.m, One-way ANOVA. *$p < 0.05$.

C. GFP^+ projection of HD hCO to hStrO on Day 5 after fusion (epifluorescent microscopy images).

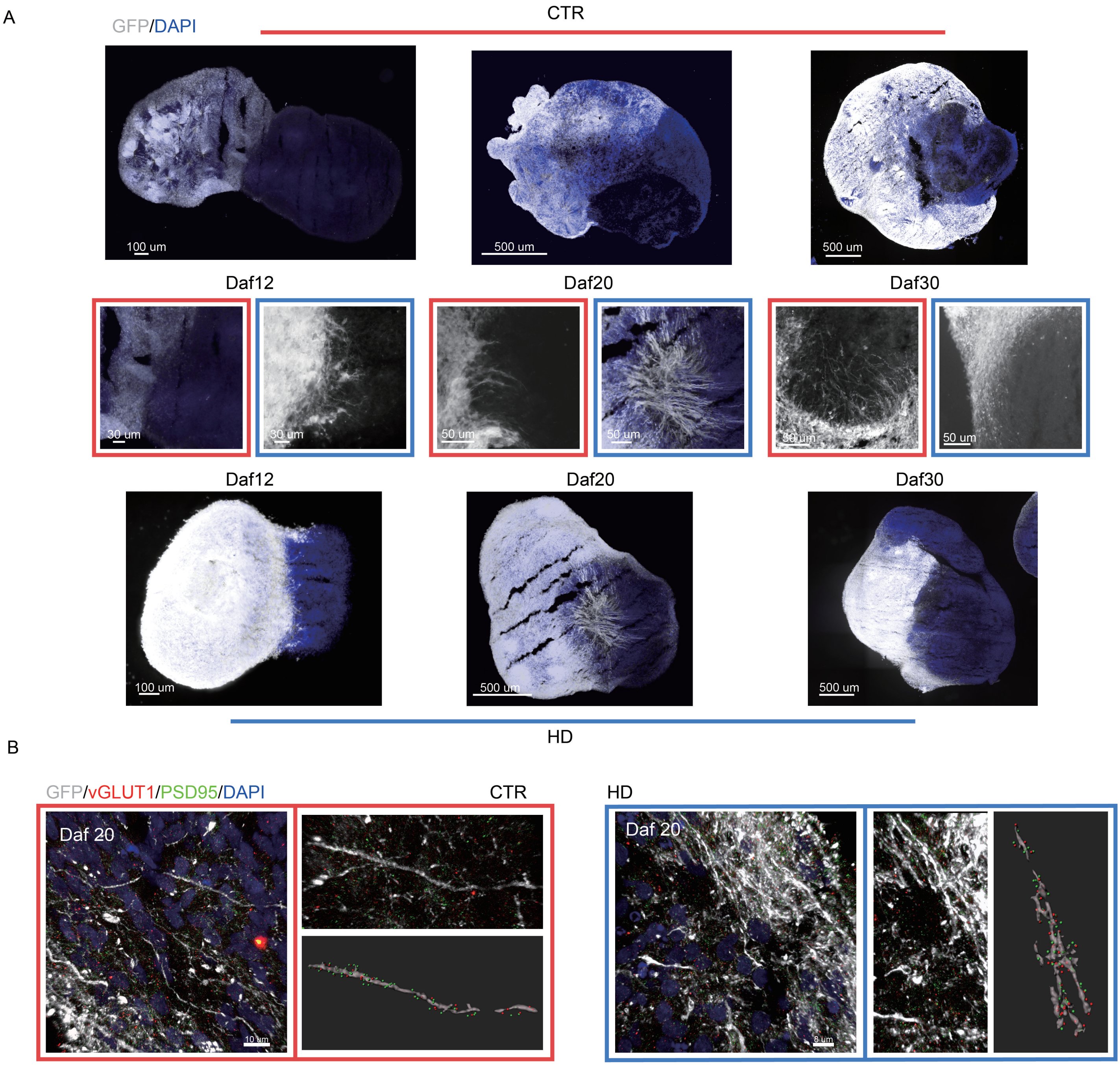

Supplementary Fig. 9. Subcortical projections increased in HD hCO.

A. The representative images of immunostaining with GFP antibody revealed the projections in CTR and HD at 12, 20, and 30 Days after fusion. The formation and projecting of the subcortical projections in HD hCO are earlier than CTR.

B. Immunostaining images of vGLUT1 and PSD95 antibodies in 20 Days after fusion revealed the synapse formation in cortical projection (arrows, synapses).

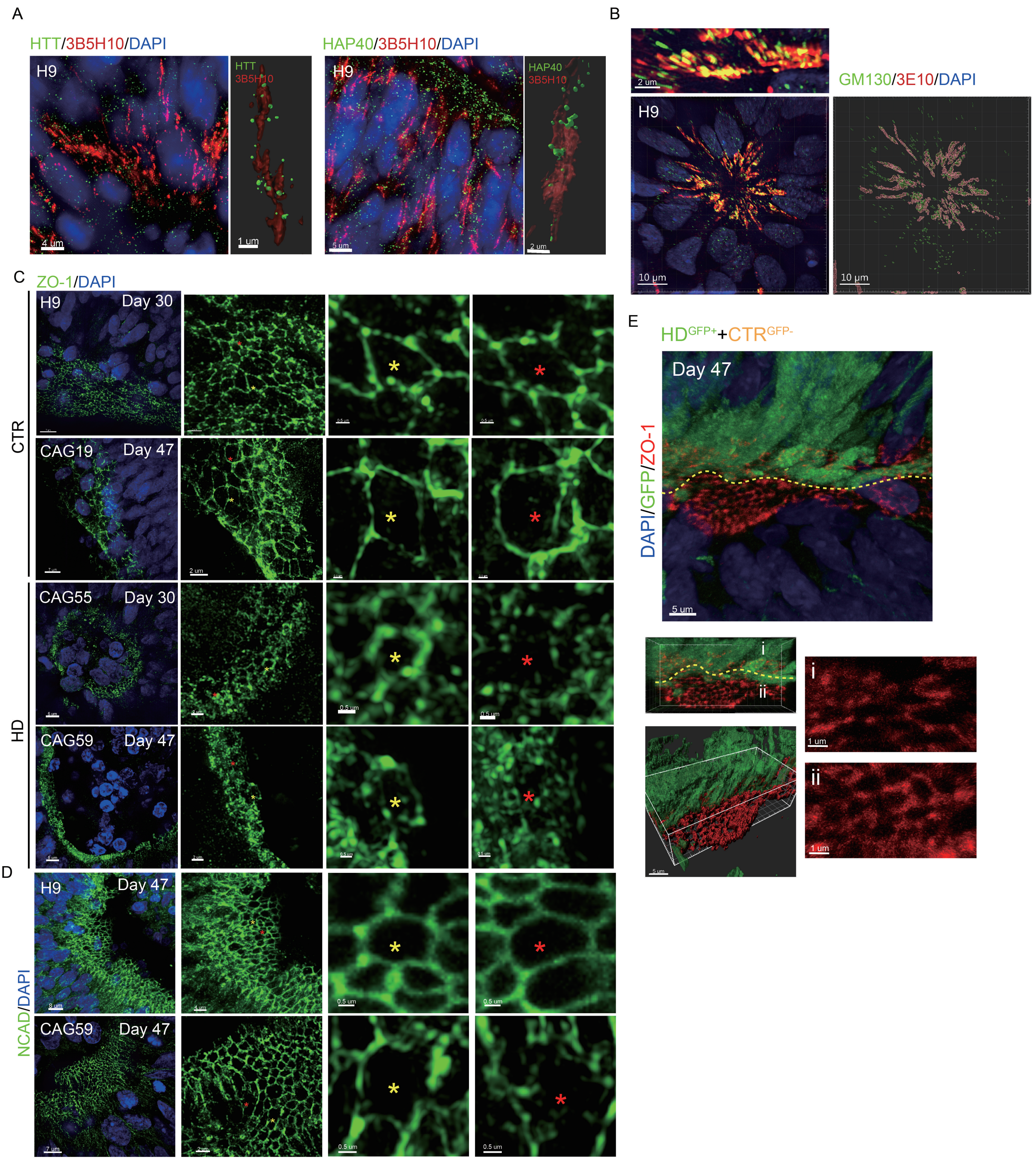

Supplementary Fig. 10. The characteristics of HAP40 on polyQ assemblies and impaired junctional complexes in HD-hCOs

A. Representative SIM images of 3B5H10, HTT, and HAP40 antibodies' immunostaining on Day 30 showed that HAP40 conjugated with polyQ assemblies.

B. Representative SIM images of immunostaining with GM130 and 3E10 antibodies on

hCOs on Day 30.

C. Representative images of ZO-1 antibody immunostaining on Day 30 and 47 showed impaired TJs in the neuroepithelium of HD-hCOs.

D. Representative SIM images of NCAD antibodies' immunostaining on Day 47 showed impaired AJs in the neuroepithelium of HD-hCOs.

E. Magnification and 3D view of ZO-1 antibody immunostained TJs surrounded mass GFP expressing protrusions. The yellow dashed line marked the margin between the GFP^+ and GFP^- cell populations.

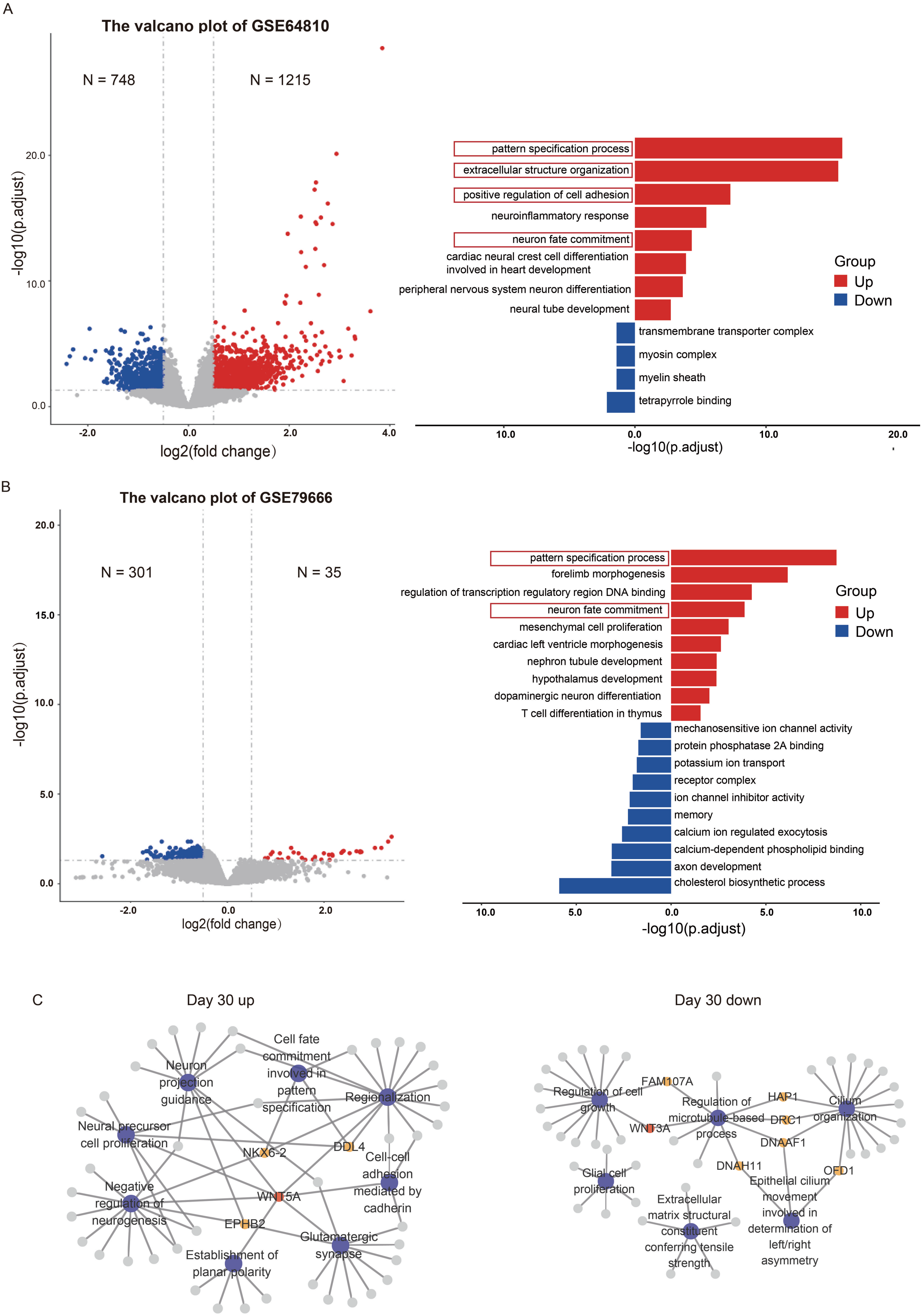
A
The valcano plot of GSE64810
N = 748
N = 1215
-log10(p.adjust)
log2(fold change)
pattern specification process
extracellular structure organization
positive regulation of cell adhesion
neuroinflammatory response
neuron fate commitment
cardiac neural crest cell differentiation involved in heart development
peripheral nervous system neuron differentiation
neural tube development
transmembrane transporter complex
myosin complex
myelin sheath
tetrapyrrole binding
Group
Up
Down
-log10(p.adjust)
B
The valcano plot of GSE79666
N = 301
N = 35
-log10(p.adjust)
log2(fold change)
pattern specification process
forelimb morphogenesis
regulation of transcription regulatory region DNA binding
neuron fate commitment
mesenchymal cell proliferation
cardiac left ventricle morphogenesis
nephron tubule development
hypothalamus development
dopaminergic neuron differentiation
T cell differentiation in thymus
mechanosensitive ion channel activity
protein phosphatase 2A binding
potassium ion transport
receptor complex
ion channel inhibitor activity
memory
calcium ion regulated exocytosis
calcium-dependent phospholipid binding
axon development
cholesterol biosynthetic process
Group
Up
Down
-log10(p.adjust)
C
Day 30 up
Cell fate commitment involved in pattern specification
Neuron projection guidance
Regionalization
Neural precursor cell proliferation
NKX6-2
DDL4
Cell-cell adhesion mediated by cadherin
Negative regulation of neurogenesis
WNT5A
EPHB2
Glutamatergic synapse
Establishment of planar polarity
Day 30 down
FAM107A
Regulation of cell growth
Regulation of microtubule-based process
HAP1
Cilium organization
WNT3A
DRC1
DNAAF1
Glial cell proliferation
DNAH11
OFD1
Epithelial cilium movement involved in determination of left/right asymmetry
Extracellular matrix structural constituent conferring tensile strength

Supplementary Fig. 11. Altered transcriptions in HD-hCOs

A-B. Volcano plot shows DEGs of GSE64810 (A) and GSE79666 (B) and bar plot showing the results from GO-term enrichment analysis GSE79666 and GSE64810. Red boxes indicate GO terms that also appear in our Go enrichment result.

C. GO biological processes of Day 30 DEGs related with neurodevelopment and cell adhesion. The purple dots represent pathways and other dots represent genes.

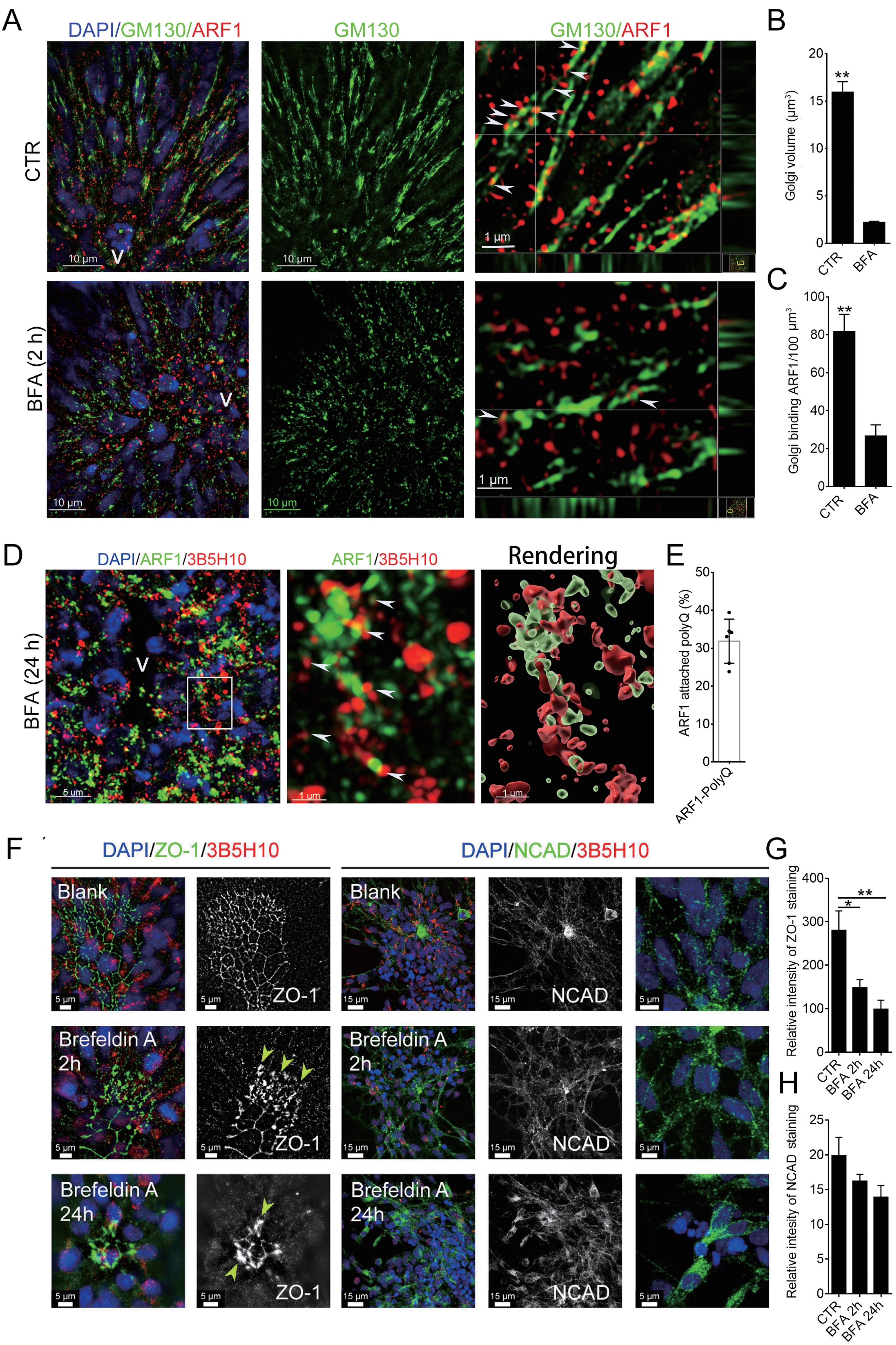

A
DAPI/GM130/ARF1
GM130
GM130/ARF1
CTR
BFA (2 h)
V
10 µm
1 µm
B
Golgi volume (µm³)
CTR
BFA
C
Golgi binding ARF1/100 µm³
D
DAPI/ARF1/3B5H10
ARF1/3B5H10
Rendering
BFA (24 h)
5 µm
E
ARF1 attached polyQ (%)
ARF1-PolyQ
F
DAPI/ZO-1/3B5H10
DAPI/NCAD/3B5H10
Blank
Brefeldin A 2h
Brefeldin A 24h
ZO-1
NCAD
15 µm
G
Relative intensity of ZO-1 staining
CTR
BFA 2h
BFA 24h
H
Relative intesity of NCAD staining

Supplementary Fig. 12. ARF1 mediates the attachment of 3B5H10⁺ polyQ assemblies to Golgi stacks

A. Representative 3D SIM images of immunostaining with GM130 and ARF1 antibodies in the neural tubes of CTR-hCOs (H9) treated by BFA. The middle panel is a GM130 staining image that reveals extended Golgi stacks in CTR-hCOs and fragmented Golgi in CTR-hCOs (H9) treated by BFA. The right panel is a sectional view that shows the status of ARF^+ puncta. v, ventricle.

B. Comparing the volume of $GM130^+$ Golgi in the neural tube of BFA-treated hCO with those in CTR-hCOs. Data, mean ± s.e.m. t-test. **p <0.01. SIM images for analysis, n=4

C. Comparing the $GM130^+$ Golgi binding ARF1 in the neural tube of BFA-treated hCO with those in CTR-hCOs. Data, mean ± s.e.m. t-test. **p <0.01. SIM images for analysis, n⩾5

D. Representative 3D SIM images of immunostaining with 3B5H10 and ARF1 antibodies in the neural tubes of CTR-hCOs treated with BFA for 24 h. The right panel is the rendering images of 3B5H10 and ARF1 staining by IMARIS surface. The boxed region in the right panel is the magnified part in the middle panel (ARF1 and 3BH5H10). v, ventricle.

E. The count of polyQ puncta of HTTs attached by ARF^+ puncta in the neural tubes of CTR-hCOs treated with BFA for 24 h.

F-G. Representative 3D SIM images of immunostaining with 3B5H10, ZO-1, and NCAD antibodies in 2D neural rosettes (C). Comparing the relative intensity of ZO-1 (D) and NCAD (E) before and after BFA intervention. Data, means ± s.e.m. One-way ANOVA. **p <0.01, ***p <0.001, ns, no significance.

SUPPLEMENTARY TABLES

Suppl.Table 1. Gene Ontology (GO) functional enrichment analysis of DEGs

Go_Up_Day30

ID	Description	GeneRatio	pvalue	p.adjust	geneID
GO:0003002	regionalization	16/157	3.54E-08	9.40E-05	3169/390992/84504/2735/3170/5076/201164/64388/6091/56603/7474/121643/7832/80319/54567/5727
GO:0010721	negative regulation of cell development	15/157	1.25E-07	0.000165914	2048/84504/79727/56963/9745/6653/359845/7025/116/7474/22882/4880/10501/85458/6658
GO:0021953	central nervous system neuron differentiation	11/157	3.01E-07	0.000267032	2048/84504/2049/6091/7474/8321/121643/7832/54567/4915/5727
GO:0050768	negative regulation of neurogenesis	13/157	7.78E-07	0.000408453	2048/84504/79727/56963/9745/6653/7025/116/7474/22882/10501/85458/6658
GO:0009953	dorsal/ventral pattern formation	8/157	8.90E-07	0.000408453	3169/84504/2735/64388/121643/80319/54567/5727
GO:0007389	pattern specification process	16/157	9.22E-07	0.000408453	3169/390992/84504/2735/3170/5076/201164/64388/6091/56603/7474/121643/7832/80319/54567/5727
GO:0051961	negative regulation of nervous system development	13/157	1.63E-06	0.000617356	2048/84504/79727/56963/9745/6653/7025/116/7474/22882/10501/85458/6658
GO:0061564	axon development	16/157	4.77E-06	0.001534937	26053/2048/8013/2049/23767/5076/56963/6091/7474/22854/10501/6498/10417/2674/4915/5727
GO:0050808	synapse organization	14/157	5.20E-06	0.001534937	10882/2048/2049/23767/7474/22854/392617/8321/27253/1006/79012/1607/4915/8404
GO:0007409	axonogenesis	15/157	6.80E-06	0.001807109	26053/2048/8013/2049/23767/5076/6091/7474/22854/10501/6498/10417/2674/4915/5727
GO:0045165	cell fate commitment	11/157	1.26E-05	0.003053531	3169/84504/3170/5076/56603/8029/7474/121643/58158/54567/5727
GO:0002009	morphogenesis of an epithelium	15/157	1.46E-05	0.003241664	3169/81792/23767/23245/8549/5076/22881/7474/131405/8321/121643/55366/166336/54567/5727
GO:0021954	central nervous system neuron development	6/157	3.91E-05	0.008007597	2048/2049/6091/7474/7832/4915
GO:2000049	positive regulation of cell-cell adhesion mediated by cadherin	3/157	6.90E-05	0.010632727	3169/3170/7474
GO:0007411	axon guidance	10/157	7.53E-05	0.010632727	2048/8013/2049/23767/6091/7474/10501/10417/2674/5727
GO:0099560	synaptic membrane adhesion	4/157	7.54E-05	0.010632727	23767/22854/27253/8404
GO:0060562	epithelial tube morphogenesis	11/157	7.66E-05	0.010632727	3169/81792/8549/5076/7474/131405/8321/121643/55366/54567/5727
GO:0097485	neuron projection guidance	10/157	7.77E-05	0.010632727	2048/8013/2049/23767/6091/7474/10501/10417/2674/5727
GO:2000177	regulation of neural precursor cell proliferation	6/157	8.01E-05	0.010632727	2735/116/7474/131405/7832/54567
GO:0071772	response to BMP	8/157	8.40E-05	0.010632727	81792/56963/64388/285704/7474/8321/6498/222008
GO:0071773	cellular response to BMP stimulus	8/157	8.40E-05	0.010632727	81792/56963/64388/285704/7474/8321/6498/222008
GO:0050807	regulation of synapse organization	9/157	9.07E-05	0.010959876	2048/2049/23767/7474/8321/1006/79012/1607/4915
GO:0001655	urogenital system development	11/157	9.85E-05	0.011171543	3169/2048/2735/2049/79727/8549/5076/56603/7474/55366/5727
GO:0045665	negative regulation of neuron differentiation	9/157	0.000100834	0.011171543	2048/56963/9745/7025/7474/22882/10501/85458/6658
GO:0050803	regulation of synapse structure or activity	9/157	0.000111925	0.011904359	2048/2049/23767/7474/8321/1006/79012/1607/4915
GO:0021554	optic nerve development	3/157	0.000124845	0.012294934	2048/5076/89797
GO:0099550	trans-synaptic signaling, modulating synaptic transmission	3/157	0.000124845	0.012294934	2048/10675/4915
GO:1905330	regulation of morphogenesis of an epithelium	7/157	0.000148167	0.014070549	81792/5076/22881/7474/8321/55366/166336
GO:0071542	dopaminergic neuron differentiation	4/157	0.000213247	0.019433923	3169/3170/7474/8321
GO:0061351	neural precursor cell proliferation	7/157	0.000221423	0.019433923	116/7474/131405/7832/58158/85458/54567
GO:0035567	non-canonical Wnt signaling pathway	6/157	0.000234163	0.019433923	22881/11211/7474/8321/26523/166336
GO:0000578	embryonic axis specification	4/157	0.000238326	0.019433923	201164/7474/80319/5727
GO:0060579	ventral spinal cord interneuron fate commitment	3/157	0.000253412	0.019433923	84504/121643/54567
GO:0060581	cell fate commitment involved in pattern specification	3/157	0.000253412	0.019433923	84504/121643/54567
GO:0051963	regulation of synapse assembly	6/157	0.000260228	0.019433923	2048/2049/23767/7474/8321/4915
GO:0043010	camera-type eye development	10/157	0.000263114	0.019433923	2048/1411/5076/7474/121643/58158/6498/10586/54567/4915
GO:0032526	response to retinoic acid	6/157	0.000274088	0.019697275	5076/56603/11211/7474/19/5727
GO:0071300	cellular response to retinoic acid	5/157	0.000300151	0.021002683	5076/56603/11211/7474/19
GO:2000047	regulation of cell-cell adhesion mediated by cadherin	3/157	0.000309952	0.021132392	3169/3170/7474
GO:0030900	forebrain development	11/157	0.000359387	0.02312905	2048/2735/8013/2049/6091/116/7474/7832/85458/6658/4915
GO:0071695	anatomical structure maturation	7/157	0.000361873	0.02312905	3169/10882/27289/5076/359845/7474/4880
GO:0021514	ventral spinal cord interneuron differentiation	3/157	0.000374031	0.02312905	84504/121643/54567
GO:0061081	positive regulation of myeloid leukocyte cytokine production involved in immune response	3/157	0.000374031	0.02312905	8013/7474/10417
GO:0050919	negative chemotaxis	4/157	0.000396456	0.023958587	23767/6091/7474/10501
GO:2000027	regulation of animal organ morphogenesis	8/157	0.000408736	0.024151742	2735/5076/6091/22881/7474/8321/55366/166336
GO:0044331	cell-cell adhesion mediated by cadherin	4/157	0.000435314	0.024643633	3169/3170/7474/1006
GO:0048762	mesenchymal cell differentiation	8/157	0.000435596	0.024643633	3169/3170/5076/84541/359845/7474/10501/55223
GO:0021915	neural tube development	7/157	0.000454908	0.025200018	3169/390992/5076/7474/131405/8321/5727
GO:0021983	pituitary gland development	4/157	0.000476813	0.025874405	2735/116/7474/6658
GO:0060485	mesenchyme development	9/157	0.000496104	0.026055174	3169/3170/5076/6091/84541/359845/7474/10501/55223
GO:0021675	nerve development	5/157	0.000499742	0.026055174	390992/26018/2048/5076/89797
GO:0098742	cell-cell adhesion via plasma-membrane adhesion molecules	9/157	0.000509549	0.026055598	23767/6091/22854/57451/120114/27253/1006/56147/8404
GO:0007416	synapse assembly	7/157	0.000526885	0.02643374	2048/2049/23767/7474/8321/27253/4915
GO:0001736	establishment of planar polarity	5/157	0.000562357	0.027187402	23245/22881/7474/8321/166336
GO:0007164	establishment of tissue polarity	5/157	0.000562357	0.027187402	23245/22881/7474/8321/166336
GO:0016055	Wnt signaling pathway	12/157	0.000605341	0.028333789	2735/8549/22881/11211/7474/8321/26523/55366/85458/80319/55182/166336
GO:0060039	pericardium development	3/157	0.000615441	0.028333789	23767/7474/54567
GO:0021517	ventral spinal cord development	4/157	0.00061817	0.028333789	84504/121643/54567/5727
GO:0198738	cell-cell signaling by wnt	12/157	0.000628693	0.028333789	2735/8549/22881/11211/7474/8321/26523/55366/85458/80319/55182/166336

GO:0009798	axis specification	5/157	0.000667095	0.029259753	3170/201164/7474/80319/5727
GO:0030850	prostate gland development	4/157	0.000671247	0.029259753	3169/2735/7474/5727
GO:0060070	canonical Wnt signaling pathway	9/157	0.00073143	0.031251197	2735/8549/22881/11211/7474/8321/55366/85458/55182
GO:0001708	cell fate specification	5/157	0.000744609	0.031251197	3169/84504/3170/5076/5727
GO:0021545	cranial nerve development	4/157	0.000786948	0.031251197	390992/2048/5076/89797
GO:0034260	negative regulation of GTPase activity	4/157	0.000786948	0.031251197	116/11211/23677/51291
GO:0030177	positive regulation of Wnt signaling pathway	6/157	0.00078745	0.031251197	8549/22881/7474/55366/85458/55182
GO:0035270	endocrine system development	6/157	0.00078745	0.031251197	84504/2735/3170/116/7474/6658
GO:0021513	spinal cord dorsal/ventral patterning	3/157	0.0008211	0.031824763	84504/121643/54567
GO:0006029	proteoglycan metabolic process	5/157	0.000828661	0.031824763	2135/50859/81792/9348/10675
GO:0001654	eye development	10/157	0.000849469	0.031824763	2048/1411/5076/7474/121643/58158/6498/10586/54567/4915
GO:0009994	oocyte differentiation	4/157	0.000849777	0.031824763	3625/8549/201164/4880
GO:0150063	visual system development	10/157	0.000868041	0.032057245	2048/1411/5076/7474/121643/58158/6498/10586/54567/4915
GO:0050770	regulation of axonogenesis	7/157	0.000881373	0.032103727	2048/2049/6091/7474/10501/6498/4915
GO:0030514	negative regulation of BMP signaling pathway	4/157	0.000916063	0.032916362	64388/7474/8321/6498
GO:0048880	sensory system development	10/157	0.000965989	0.032990211	2048/1411/5076/7474/121643/58158/6498/10586/54567/4915
GO:0001843	neural tube closure	5/157	0.000967746	0.032990211	5076/7474/131405/8321/5727
GO:0022404	molting cycle process	5/157	0.000967746	0.032990211	26018/8549/7474/55366/55504
GO:0022405	hair cycle process	5/157	0.000967746	0.032990211	26018/8549/7474/55366/55504
GO:0021515	cell differentiation in spinal cord	4/157	0.000985907	0.033183868	84504/121643/54567/5727
GO:0060606	tube closure	5/157	0.001017749	0.033827425	5076/7474/131405/8321/5727
GO:0001837	epithelial to mesenchymal transition	6/157	0.001039032	0.034108462	3169/3170/84541/359845/7474/55223
GO:0003176	aortic valve development	3/157	0.00106582	0.034144752	7133/6091/54567
GO:0021511	spinal cord patterning	3/157	0.00106582	0.034144752	84504/121643/54567
GO:0021537	telencephalon development	8/157	0.001284797	0.040574862	2048/8013/2049/6091/7474/7832/85458/4915
GO:0001738	morphogenesis of a polarized epithelium	5/157	0.001297053	0.040574862	23245/22881/7474/8321/166336
GO:0014020	primary neural tube formation	5/157	0.001359084	0.041428615	5076/7474/131405/8321/5727
GO:0042472	inner ear morphogenesis	5/157	0.001359084	0.041428615	26018/2048/8013/5076/7474
GO:0030111	regulation of Wnt signaling pathway	9/157	0.001381	0.041428615	2735/8549/22881/7474/8321/55366/85458/80319/55182
GO:0090596	sensory organ morphogenesis	8/157	0.001386667	0.041428615	26018/2048/8013/5076/56603/7474/121643/4915
GO:1903779	regulation of cardiac conduction	4/157	0.001485443	0.043886581	845/3270/4880/489
GO:0061082	myeloid leukocyte cytokine production	3/157	0.001682774	0.049170277	8013/7474/10417
GO:0050769	positive regulation of neurogenesis	11/157	0.001716248	0.049603312	3169/8835/2048/84504/79727/6091/116/7474/8321/6498/491
GO:0030073	insulin secretion	7/157	0.001764019	0.049621935	9066/3625/3170/387597/8620/116/80024
GO:0008630	intrinsic apoptotic signaling pathway in response to DNA damage	5/157	0.001778281	0.049621935	7133/57007/138151/666/6498
GO:0048754	branching morphogenesis of an epithelial tube	6/157	0.001782718	0.049621935	3169/5076/7474/55366/54567/5727
GO:0009880	embryonic pattern specification	4/157	0.00179154	0.049621935	201164/7474/80319/5727
GO:0098978	glutamatergic synapse	11/164	0.00017713	0.023206843	2048/9066/23767/7474/22854/10675/27253/1006/79012/1607/8404
GO:0033267	axon part	11/164	0.000305808	0.023206843	26053/9066/23767/7133/8620/116/57451/5874/1006/4915/5727
GO:0043235	receptor complex	11/164	0.000505771	0.023206843	2048/4547/2049/7133/8029/84171/2893/3696/2570/2674/491
GO:0043679	axon terminus	6/164	0.000513129	0.023206843	9066/23767/8620/116/1006/4915
GO:0031225	anchored component of membrane	7/164	0.000550371	0.023206843	79465/56963/285704/56341/22854/5874/2674
GO:0150034	distal axon	9/164	0.000587515	0.023206843	26053/9066/23767/8620/116/57451/1006/4915/5727
GO:0044306	neuron projection terminus	6/164	0.001067593	0.036145641	9066/23767/8620/116/1006/4915
GO:0046658	anchored component of plasma membrane	4/164	0.001395234	0.041333797	79465/285704/56341/22854
GO:0000982	transcription factor activity, RNA polymerase II proximal promoter sequence-specific DNA binding	13/150	0.000117866	0.041606662	3169/9516/390992/84504/8013/253738/9745/7025/138151/7832/6498/2649/4808

Go_Down_Day30

ID	Description	GeneRatio	pvalue	p.adjust	geneID
GO:0043062	extracellular structure organization	24/240	5.58E-10	1.75E-06	9509/2192/5654/89780/8751/81621/1288/255631/7276/348/1287/341/10516/3909/56999/9719/3383/25975/1301/10549/4015/2331/1292/3371
GO:0030198	extracellular matrix organization	22/240	1.05E-09	1.75E-06	9509/2192/5654/89780/8751/81621/1288/255631/7276/1287/10516/3909/56999/9719/3383/25975/1301/10549/4015/2331/1292/3371
GO:0035082	axoneme assembly	8/240	1.17E-06	0.001252191	286207/387885/374407/6674/123872/92749/85452/200162
GO:0003341	cilium movement	8/240	1.50E-06	0.001252191	8701/83659/387885/123872/92749/200162/26074/8481
GO:1902991	regulation of amyloid precursor protein catabolic process	5/240	1.23E-05	0.00819822	9001/347731/348/1191/4067
GO:0014009	glial cell proliferation	6/240	1.83E-05	0.009276076	2119/1191/5179/706/4067/407006
GO:0001578	microtubule bundle formation	8/240	2.05E-05	0.009276076	286207/387885/374407/6674/123872/92749/85452/200162
GO:0009308	amine metabolic process	8/240	2.22E-05	0.009276076	6611/7200/3251/3067/1312/6817/6303/1728
GO:0044782	cilium organization	16/240	4.18E-05	0.01454476	4952/9001/83659/286207/387885/374407/6674/10257/123872/92749/647309/375307/85452/200162/26074/8481
GO:0007626	locomotory behavior	11/240	4.79E-05	0.01454476	7200/793/3251/348/2119/3766/5179/3358/1392/477/58524
GO:0060541	respiratory system development	11/240	4.79E-05	0.01454476	9509/2719/1512/157506/2034/123872/9719/4015/1392/3371/5916
GO:0042987	amyloid precursor protein catabolic process	5/240	6.23E-05	0.017341107	9001/347731/348/1191/4067
GO:0060252	positive regulation of glial cell proliferation	4/240	7.34E-05	0.017676831	2119/706/4067/407006
GO:0030324	lung development	10/240	7.41E-05	0.017676831	9509/2719/1512/157506/2034/123872/9719/4015/1392/3371
GO:0044106	cellular amine metabolic process	7/240	8.73E-05	0.018079092	6611/7200/3251/3067/1312/6303/1728
GO:0030323	respiratory tube development	10/240	9.00E-05	0.018079092	9509/2719/1512/157506/2034/123872/9719/4015/1392/3371
GO:1902003	regulation of amyloid-beta formation	4/240	9.20E-05	0.018079092	9001/347731/348/1191
GO:0050804	modulation of chemical synaptic transmission	16/240	0.000120097	0.021706356	9001/89780/793/348/57406/4920/11170/3766/2890/5028/143425/4889/1392/9211/477/2918
GO:0099177	regulation of trans-synaptic signaling	16/240	0.000123442	0.021706356	9001/89780/793/348/57406/4920/11170/3766/2890/5028/143425/4889/1392/9211/477/2918
GO:0051384	response to glucocorticoid	9/240	0.000145087	0.02423677	54541/7200/3485/11170/133522/133/3383/1392/9076
GO:0043270	positive regulation of ion transport	12/240	0.00015735	0.02503357	1756/9001/7114/7200/348/219527/51083/844/5028/706/1392/5652
GO:0031099	regeneration	10/240	0.000178482	0.026963498	8751/4856/133/706/2027/9076/728/407006/3371/4147
GO:0042063	gliogenesis	12/240	0.000194421	0.026963498	4920/2119/1191/3766/5028/5179/4919/706/4067/407006/170825/4147
GO:0016101	diterpenoid metabolic process	7/240	0.000195047	0.026963498	2239/2719/7276/157506/348/1392/6121
GO:0003351	epithelial cilium movement	4/240	0.000201762	0.026963498	8701/123872/200162/8481
GO:0003418	growth plate cartilage chondrocyte differentiation	4/240	0.000239661	0.027285326	309/1292/4147/5916
GO:0060287	epithelial cilium movement involved in determination of left/right asymmetry	3/240	0.00024216	0.027285326	8701/123872/8481
GO:1902430	negative regulation of amyloid-beta formation	3/240	0.00024216	0.027285326	9001/348/1191
GO:0045471	response to ethanol	8/240	0.000254733	0.027285326	7200/1365/5179/3383/1392/9076/3371/1728
GO:0015849	organic acid transport	13/240	0.000261629	0.027285326	26266/9121/28232/7200/348/11001/201780/10257/3766/23428/4889/6567/477
GO:0046942	carboxylic acid transport	13/240	0.000261629	0.027285326	26266/9121/28232/7200/348/11001/201780/10257/3766/23428/4889/6567/477
GO:0006874	cellular calcium ion homeostasis	16/240	0.000263272	0.027285326	1756/9001/793/348/553/844/133/2890/5028/309/3358/255061/4067/728/477/55013
GO:0034205	amyloid-beta formation	4/240	0.00028242	0.027285326	9001/347731/348/1191
GO:0048678	response to axon injury	6/240	0.000294118	0.027285326	706/4067/3956/407006/3371/4147
GO:0034765	regulation of ion transmembrane transport	16/240	0.000305193	0.027285326	1756/9001/7114/79026/219527/54102/30818/51083/3769/844/3766/3756/56659/1392/4067/477
GO:0060271	cilium assembly	14/240	0.000321071	0.027285326	4952/9001/83659/286207/387885/374407/6674/10257/123872/92749/647309/85452/200162/8481
GO:0031960	response to corticosteroid	9/240	0.000325541	0.027285326	54541/7200/3485/11170/133522/133/3383/1392/9076
GO:0021534	cell proliferation in hindbrain	3/240	0.000329783	0.027285326	1950/22809/728
GO:0051953	negative regulation of amine transport	4/240	0.000330389	0.027285326	7200/5028/4889/1392
GO:0060632	regulation of microtubule-based movement	4/240	0.000330389	0.027285326	8701/9001/123872/92749
GO:0006816	calcium ion transport	15/240	0.000342618	0.027285326	1756/9001/89780/79026/80036/844/3383/1950/706/309/3358/1392/4067/477/55013
GO:0033555	multicellular organismal response to stress	6/240	0.000343006	0.027285326	348/1312/5179/706/3358/1392
GO:0055074	calcium ion homeostasis	16/240	0.000361293	0.028071607	1756/9001/793/348/553/844/133/2890/5028/309/3358/255061/4067/728/477/55013
GO:0042692	muscle cell differentiation	14/240	0.000388316	0.029485558	1756/7138/89780/4772/3856/85461/4856/3880/844/133/3756/50937/407006/124857
GO:0006721	terpenoid metabolic process	7/240	0.000414773	0.029626456	2239/2719/7276/157506/348/1392/6121
GO:0010959	regulation of metal ion transport	14/240	0.000420628	0.029626456	1756/9001/79026/219527/30818/51083/844/3383/1950/706/1392/4067/5652/477
GO:0042982	amyloid precursor protein metabolic process	5/240	0.000422732	0.029626456	9001/347731/348/1191/4067
GO:0035641	locomotory exploration behavior	3/240	0.000435504	0.029626456	348/5179/1392
GO:1902992	negative regulation of amyloid precursor protein catabolic process	3/240	0.000435504	0.029626456	9001/348/1191
GO:0002026	regulation of the force of heart contraction	4/240	0.000443377	0.029626456	4625/133/477/8862
GO:0071804	cellular potassium ion transport	10/240	0.000466018	0.029941667	3775/219527/30818/51083/3769/3766/84679/3756/56659/477
GO:0071805	potassium ion transmembrane transport	10/240	0.000466018	0.029941667	3775/219527/30818/51083/3769/3766/84679/3756/56659/477
GO:0048608	reproductive structure development	15/240	0.000497411	0.03135565	5654/8751/3856/157506/2034/4920/3880/151449/133/3383/10549/431707/3371/5916/7274
GO:0003413	chondrocyte differentiation involved in endochondral bone morphogenesis	4/240	0.000509116	0.031499165	309/1292/4147/5916
GO:0061458	reproductive system development	15/240	0.000534673	0.032478967	5654/8751/3856/157506/2034/4920/3880/151449/133/3383/10549/431707/3371/5916/7274
GO:0014819	regulation of skeletal muscle contraction	3/240	0.000560743	0.032867405	1756/4625/844
GO:1902993	positive regulation of amyloid precursor protein catabolic process	3/240	0.000560743	0.032867405	347731/1191/4067
GO:0042445	hormone metabolic process	10/240	0.000579403	0.033375636	7276/157506/1312/51083/6817/133/2690/706/1392/6121
GO:0072503	cellular divalent inorganic cation homeostasis	16/240	0.000599339	0.033607693	1756/9001/793/348/553/844/133/2890/5028/309/3358/255061/4067/728/477/55013
GO:0048545	response to steroid hormone	14/240	0.00060355	0.033607693	54541/7200/4306/3485/11170/133522/133/3383/706/4015/1392/9076/477/5916
GO:1903522	regulation of blood circulation	11/240	0.000652968	0.034724932	1756/2034/3775/553/4625/844/133/3383/477/2697/8862
GO:0070286	axonemal dynein complex assembly	4/240	0.000660903	0.034724932	387885/6674/123872/92749

GO:0006584	catecholamine metabolic process	5/240	0.000665189	0.034724932	3251/3067/2034/1312/6817
GO:0009712	catechol-containing compound metabolic process	5/240	0.000665189	0.034724932	3251/3067/2034/1312/6817
GO:0051480	regulation of cytosolic calcium ion concentration	13/240	0.000687676	0.035346533	1756/9001/793/553/844/133/2890/5028/3358/255061/4067/728/477
GO:0021527	spinal cord association neuron differentiation	3/240	0.000706854	0.035781804	89780/151449/170825
GO:0031102	neuron projection regeneration	5/240	0.000724062	0.03586014	133/706/407006/3371/4147
GO:0008015	blood circulation	16/240	0.000729868	0.03586014	1756/7138/348/2034/3775/553/4625/844/133/5028/3383/1392/255061/477/2697/8862
GO:0034754	cellular hormone metabolic process	7/240	0.000758634	0.036733287	7276/157506/1312/6817/133/706/6121
GO:0019229	regulation of vasoconstriction	5/240	0.000786744	0.037186803	553/133/3383/477/8862
GO:0007568	aging	12/240	0.000790261	0.037186803	84417/3485/133/5028/5179/3383/706/4889/604/2027/9076/1728
GO:0060251	regulation of glial cell proliferation	4/240	0.000842227	0.038528721	2119/706/4067/407006
GO:0003013	circulatory system process	16/240	0.000865569	0.038528721	1756/7138/348/2034/3775/553/4625/844/133/5028/3383/1392/255061/477/2697/8862
GO:0003352	regulation of cilium movement	3/240	0.000875131	0.038528721	8701/123872/92749
GO:0043101	purine-containing compound salvage	3/240	0.000875131	0.038528721	56952/3251/2766
GO:0051283	negative regulation of sequestering of calcium ion	7/240	0.0008835	0.038528721	1756/9001/844/309/3358/4067/477
GO:0001523	retinoid metabolic process	6/240	0.000887971	0.038528721	2239/2719/7276/157506/348/6121
GO:0009914	hormone transport	12/240	0.000907421	0.038867851	7200/7276/4856/51083/133/5028/143425/3358/1392/6567/4067/8862
GO:0051282	regulation of sequestering of calcium ion	7/240	0.000975371	0.041249543	1756/9001/844/309/3358/4067/477
GO:0070838	divalent metal ion transport	15/240	0.001013535	0.042327739	1756/9001/89780/79026/80036/844/3383/1950/706/309/3358/1392/4067/477/55013
GO:0051146	striated muscle cell differentiation	11/240	0.001029221	0.042452183	1756/7138/89780/3856/85461/4856/3880/844/3756/50937/124857
GO:0072511	divalent inorganic cation transport	15/240	0.001081597	0.042709327	1756/9001/89780/79026/80036/844/3383/1950/706/309/3358/1392/4067/477/55013
GO:0015718	monocarboxylic acid transport	8/240	0.001085581	0.042709327	9121/28232/7200/348/11001/201780/10257/6567
GO:0001558	regulation of cell growth	14/240	0.001124508	0.042709327	684/5654/89780/8751/81621/348/10516/4856/51083/3485/11170/604/9211/2697
GO:0006720	isoprenoid metabolic process	7/240	0.001127101	0.042709327	2239/2719/7276/157506/348/1392/6121
GO:0051208	sequestering of calcium ion	7/240	0.001127101	0.042709327	1756/9001/844/309/3358/4067/477
GO:0050806	positive regulation of synaptic transmission	8/240	0.001130565	0.042709327	9001/793/348/4920/11170/143425/1392/9211
GO:0006813	potassium ion transport	10/240	0.001133911	0.042709327	3775/219527/30818/51083/3769/3766/84679/3756/56659/477
GO:0051924	regulation of calcium ion transport	10/240	0.001170249	0.042709327	1756/9001/79026/844/3383/1950/706/1392/4067/477
GO:0001662	behavioral fear response	4/240	0.001176072	0.042709327	348/5179/3358/1392
GO:0002209	behavioral defense response	4/240	0.001176072	0.042709327	348/5179/3358/1392
GO:0003417	growth plate cartilage development	4/240	0.001176072	0.042709327	309/1292/4147/5916
GO:0050435	amyloid-beta metabolic process	4/240	0.001305334	0.04689379	9001/347731/348/1191
GO:0060047	heart contraction	10/240	0.001366505	0.048569062	1756/7138/2034/3775/4625/844/133/477/2697/8862
GO:0051952	regulation of amine transport	6/240	0.001411387	0.048792335	7200/5028/143425/4889/1392/477
GO:0002576	platelet degranulation	7/240	0.001420541	0.048792335	7114/1191/10257/8407/1950/4067/10184
GO:0032886	regulation of microtubule-based process	9/240	0.001421112	0.048792335	4281/8701/9001/89780/10735/11170/123872/92749/22809
GO:0042596	fear response	4/240	0.001444203	0.048792335	348/5179/3358/1392
GO:0034599	cellular response to oxidative stress	11/240	0.001445807	0.048792335	10587/2034/10516/2119/133522/6778/5179/51765/3315/7444/1728
GO:0035296	regulation of tube diameter	7/240	0.00148579	0.049148755	348/553/133/5028/3383/477/8862
GO:0097746	regulation of blood vessel diameter	7/240	0.00148579	0.049148755	348/553/133/5028/3383/477/8862
GO:0062023	collagen-containing extracellular matrix	26/257	2.82E-13	1.01E-10	2239/9509/2192/2719/5654/81621/1288/255631/348/25878/1287/10516/4856/3909/56999/1191/50937/3383/25975/309/1301/2331/3956/1292/3371/4147
GO:0031012	extracellular matrix	29/257	1.24E-11	2.22E-09	2239/9509/2192/2719/5654/347731/81621/1288/255631/348/25878/1287/10516/4856/3909/56999/1191/9719/50937/3383/25975/309/1301/4015/2331/3956/1292/3371/4147
GO:0005788	endoplasmic reticulum lumen	17/257	4.99E-07	5.95E-05	2719/89780/1288/255631/23491/348/1287/5329/844/143888/11001/91851/5179/1301/3956/1292/3371
GO:0044420	extracellular matrix component	7/257	3.23E-06	0.000289403	2192/1288/1287/10516/3909/1301/3371
GO:0005930	axoneme	9/257	1.90E-05	0.001136184	8701/387885/374407/123872/158798/92749/85452/200162/26074
GO:0097014	ciliary plasm	9/257	2.04E-05	0.001136184	8701/387885/374407/123872/158798/92749/85452/200162/26074
GO:0031514	motile cilium	11/257	2.22E-05	0.001136184	8701/83659/8751/146845/387885/402160/374407/92749/200162/26074/84229
GO:0032838	plasma membrane bounded cell projection cytoplasm	11/257	5.66E-05	0.002533477	8701/9001/387885/374407/123872/158798/92749/85452/3315/200162/26074
GO:0043034	costamere	4/257	9.47E-05	0.003768904	1756/79026/3856/3880
GO:0005604	basement membrane	7/257	0.000204701	0.007328288	2192/1288/1287/3909/25975/3371/4147
GO:0045121	membrane raft	12/257	0.000695988	0.021368313	1756/684/79026/6778/23180/3383/199920/284119/4067/219699/477/2697
GO:0098857	membrane microdomain	12/257	0.000716256	0.021368313	1756/684/79026/6778/23180/3383/199920/284119/4067/219699/477/2697
GO:0098589	membrane region	12/257	0.000973799	0.025116892	1756/684/79026/6778/23180/3383/199920/284119/4067/219699/477/2697
GO:0005581	collagen trimer	6/257	0.000982225	0.025116892	1288/255631/1287/1301/4015/1292
GO:0044447	axoneme part	4/257	0.001086226	0.025924585	8701/387885/200162/26074
GO:0030018	Z disc	7/257	0.001356479	0.030117399	1756/3856/4625/3880/844/91977/3315
GO:0016327	apicolateral plasma membrane	3/257	0.001558181	0.030117399	3856/1365/3880
GO:0098793	presynapse	15/257	0.001779816	0.030117399	2239/85439/9001/89780/793/85461/30818/3766/2890/3756/5028/143425/5179/4919/2918
GO:0016010	dystrophin-associated glycoprotein complex	3/257	0.001832574	0.030117399	1756/3856/3880
GO:0090665	glycoprotein complex	3/257	0.001832574	0.030117399	1756/3856/3880
GO:0098644	complex of collagen trimers	3/257	0.001832574	0.030117399	1288/1287/1301
GO:0042383	sarcolemma	7/257	0.00185079	0.030117399	1756/79026/3856/3880/844/477/1292
GO:0044449	contractile fiber part	9/257	0.002082205	0.031216572	1756/7138/79026/3856/4625/3880/844/91977/3315
GO:0044441	ciliary part	14/257	0.002092731	0.031216572	4952/8701/286207/387885/374407/128344/123872/158798/92749/2986/85452/200162/26074/8481
GO:0031674	I band	7/257	0.002190093	0.031362126	1756/3856/4625/3880/844/91977/3315
GO:0030016	myofibril	9/257	0.002359595	0.032489805	1756/7138/79026/3856/4625/3880/844/91977/3315
GO:0043292	contractile fiber	9/257	0.003276092	0.043438555	1756/7138/79026/3856/4625/3880/844/91977/3315
GO:0005201	extracellular matrix structural constituent	12/242	1.56E-06	0.000800243	2192/1288/255631/25878/1287/10516/3909/1301/2331/1292/3371/4147
GO:0030020	extracellular matrix structural constituent conferring tensile strength	5/242	0.000142312	0.036431868	1288/255631/1287/1301/1292

Go_t ”_DayU0

ID	Description	GeneRatio	pvalue	p.adjust	geneID
GO:0050804	modulation of chemical synaptic transmission	42/551	5.80E-12	1.26E-08	59283/5582/1268/9465/57616/5923/3274/729956/9162/2983/2915/815/6857/5581/26045/10590/22849/783/5575/55638/5864/6809/2898/7143/6812/85358/23096/2899/776/6855/56853/9148/6529/6616/9378/23349/816/6543/5530/729993/6853/57338
GO:0099177	regulation of trans-synaptic signaling	42/551	6.27E-12	1.26E-08	59283/5582/1268/9465/57616/5923/3274/729956/9162/2983/2915/815/6857/5581/26045/10590/22849/783/5575/55638/5864/6809/2898/7143/6812/85358/23096/2899/776/6855/56853/9148/6529/6616/9378/23349/816/6543/5530/729993/6853/57338
GO:0042391	regulation of membrane potential	41/551	3.52E-11	4.71E-08	2891/6546/2561/2257/401190/1268/2558/2554/2562/9162/2915/2743/27133/23415/3746/783/2567/482/57582/2898/10861/88/85358/6262/2899/57369/3760/776/666/3747/3762/56853/9481/9378/23630/5530/642938/3756/773/388591/2556
GO:0050808	synapse organization	37/551	3.64E-10	3.65E-07	139065/56098/8828/2119/114798/56127/729956/6792/2915/25791/2743/84966/57717/347731/26045/6620/84623/783/1740/9118/3371/8536/55638/5864/9379/7143/1007/85358/56097/27445/9148/23768/9378/22891/816/6853/58512
GO:0048167	regulation of synaptic plasticity	23/551	1.99E-09	1.43E-06	57616/5923/3274/729956/9162/2915/815/26045/10590/22849/5864/6809/2898/7143/6812/85358/6855/9148/6616/816/6543/729993/57338
GO:0048168	regulation of neuronal synaptic plasticity	13/551	2.14E-09	1.43E-06	5923/729956/2915/815/5864/2898/85358/6855/9148/816/6543/729993/57338
GO:0099504	synaptic vesicle cycle	23/551	2.03E-08	9.96E-06	29993/5582/1268/9162/815/6857/140679/6620/783/5575/5864/6809/6812/776/27445/6855/6861/5533/6616/9378/148281/415117/6853
GO:0071804	cellular potassium ion transport	24/551	2.24E-08	9.96E-06	3765/7881/27133/23415/3746/84561/482/57582/9312/3739/3738/3754/88/3760/776/3747/3762/6616/23630/389816/3756/388591/6553/56660
GO:0071805	potassium ion transmembrane transport	24/551	2.24E-08	9.96E-06	3765/7881/27133/23415/3746/84561/482/57582/9312/3739/3738/3754/88/3760/776/3747/3762/6616/23630/389816/3756/388591/6553/56660
GO:0045666	positive regulation of neuron differentiation	32/551	3.06E-08	1.17E-05	57795/1745/57611/29993/2119/1268/114798/10154/6792/51761/6857/57554/1746/7732/57699/22849/81544/57698/163404/8536/650/9037/85358/5915/8507/91584/1620/9148/257194/816/1272/8437
GO:0061564	axon development	39/551	3.21E-08	1.17E-05	139065/9890/9369/57715/8013/9201/57611/84570/5293/8828/1268/114798/10154/10100/6792/51761/3670/57731/1749/4929/7070/80031/57698/5578/3371/5864/4917/7143/9037/6812/4685/9241/85358/91584/6710/10371/23768/5909/9378
GO:0010975	regulation of neuron projection development	38/551	4.26E-08	1.42E-05	57715/57611/29993/1268/29116/114798/27124/10154/6792/51761/6857/57554/25791/57699/22849/7070/80031/57698/163404/8536/7143/23108/9037/85358/8507/91584/9856/9148/10371/6616/22891/140578/257194/816/1272/5530/5396/8437
GO:0051668	localization within membrane	19/551	5.11E-08	1.58E-05	59283/23180/2119/10082/729956/57554/2743/57576/7070/1740/1012/9379/6809/2898/85358/23096/8174/6616/9378
GO:0034765	regulation of ion transmembrane transport	36/551	1.04E-07	2.82E-05	59283/6546/3765/7881/2257/5923/729956/2915/5581/27133/23415/3746/783/7070/482/9312/3739/84329/3738/3754/88/85358/6262/3760/776/3747/3762/23630/389816/4318/729993/3756/773/388591/56660/57338
GO:0007611	learning or memory	25/551	1.06E-07	2.82E-05	9369/7881/5582/6310/1268/57144/2558/5923/3274/729956/9162/2915/22849/29767/5575/9379/7143/85358/1620/6529/6616/9378/7067/6543/57338
GO:0007269	neurotransmitter secretion	20/551	1.32E-07	3.11E-05	5582/1268/9162/815/6857/140679/783/5864/9379/6809/6812/776/27445/6855/6861/6616/9378/148281/415117/6853
GO:0099643	signal release from synapse	20/551	1.32E-07	3.11E-05	5582/1268/9162/815/6857/140679/783/5864/9379/6809/6812/776/27445/6855/6861/6616/9378/148281/415117/6853
GO:0016079	synaptic vesicle exocytosis	17/551	1.44E-07	3.21E-05	5582/1268/9162/815/6857/783/5864/6809/6812/776/27445/6855/6861/6616/148281/415117/6853
GO:0060078	regulation of postsynaptic membrane potential	18/551	1.71E-07	3.60E-05	2891/6546/2561/401190/2558/2554/2562/9162/2915/2743/2567/2898/85358/56853/9378/5530/642938/2556
GO:0006813	potassium ion transport	24/551	2.03E-07	4.08E-05	3765/7881/27133/23415/3746/84561/482/57582/9312/3739/3738/3754/88/3760/776/3747/3762/6616/23630/389816/3756/388591/6553/56660
GO:0007409	axonogenesis	35/551	2.36E-07	4.51E-05	139065/9890/9369/57715/8013/9201/57611/84570/5293/8828/114798/10154/6792/51761/3670/57731/1749/4929/7070/80031/57698/5578/5864/4917/7143/9037/6812/9241/85358/91584/6710/10371/23768/5909/9378
GO:0050769	positive regulation of neurogenesis	35/551	2.92E-07	5.32E-05	57795/1745/57611/29993/2119/1268/114798/10154/6792/51761/6857/57554/1746/7732/57699/22849/81544/57698/163404/8536/6604/650/9037/85358/5915/8507/91584/1620/9148/10371/22891/257194/816/1272/8437
GO:0043113	receptor clustering	11/551	3.65E-07	6.36E-05	2119/729956/57554/2743/7070/1740/9379/2898/85358/8174/9378
GO:0050890	cognition	26/551	4.58E-07	7.65E-05	9369/7881/5582/6310/1268/57144/2558/5923/3274/729956/9162/2915/22849/29767/5575/9379/7143/85358/1620/6529/6616/9378/7067/6543/152789/57338
GO:0006836	neurotransmitter transport	25/551	5.02E-07	8.05E-05	8604/5582/1268/9162/815/6857/140679/783/5864/9379/6809/6812/6505/776/27445/6855/6861/6529/6616/9378/388662/148281/415117/6853/6534
GO:0051966	regulation of synaptic transmission, glutamatergic	12/551	5.85E-07	9.03E-05	59283/1268/57616/9162/2915/6857/2898/7143/6812/85358/2899/9378
GO:0051932	synaptic transmission, GABAergic	10/551	6.34E-07	9.42E-05	2561/1268/2558/2554/5581/2567/6812/6529/23349/2556
GO:0030900	forebrain development	30/551	9.58E-07	0.000137217	6496/6546/1745/8013/9201/8828/3670/6656/1746/1749/140679/4929/3746/84623/81544/650/3362/7143/9037/9241/26038/85358/5915/91584/3747/10371/26468/27164/152789/23314
GO:0050806	positive regulation of synaptic transmission	18/551	1.28E-06	0.00017765	59283/1268/57616/729956/6857/5581/26045/10590/783/6809/2898/7143/6812/85358/776/6616/9378/6543
GO:0048172	regulation of short-term neuronal synaptic plasticity	6/551	1.67E-06	0.000223042	729956/5864/2898/6855/6543/729993
GO:0023061	signal release	33/551	2.34E-06	0.000302779	5582/1268/9162/3670/815/6857/5581/140679/5019/2587/783/10411/5578/55638/5864/9379/6809/111/6812/91608/7447/776/3747/27445/6855/6861/6616/9378/148281/415117/5530/773/6853
GO:0035249	synaptic transmission, glutamatergic	13/551	2.55E-06	0.000316881	2891/59283/1268/57616/9162/2915/6857/2898/7143/6812/85358/2899/9378
GO:0021879	forebrain neuron differentiation	10/551	2.61E-06	0.000316881	1745/8828/6656/1746/1749/85358/10371/26468/27164/23314
GO:0017156	calcium ion regulated exocytosis	17/551	3.27E-06	0.000385449	5582/1268/9162/815/6857/783/5864/6809/6812/776/27445/6855/6861/6616/148281/415117/6853
GO:0099003	vesicle-mediated transport in synapse	20/551	3.50E-06	0.000387362	29993/5582/1268/9162/815/6857/6620/783/5864/6809/6812/776/27445/6855/6861/5533/6616/148281/415117/6853
GO:0048489	synaptic vesicle transport	17/551	3.57E-06	0.000387362	5582/1268/9162/815/6857/783/5864/6809/6812/776/27445/6855/6861/6616/148281/415117/6853
GO:0097480	establishment of synaptic vesicle localization	17/551	3.57E-06	0.000387362	5582/1268/9162/815/6857/783/5864/6809/6812/776/27445/6855/6861/6616/148281/415117/6853
GO:0007158	neuron cell-cell adhesion	6/551	4.23E-06	0.00044623	9369/460/9379/7143/4685/9378
GO:0021953	central nervous system neuron differentiation	18/551	5.11E-06	0.000525193	1745/9201/8828/3670/6656/57731/1746/1749/4929/81544/51286/26038/85358/91584/10371/26468/27164/23314
GO:0035637	multicellular organismal signaling	18/551	5.53E-06	0.000537194	59283/6546/2257/57731/783/482/2898/4882/7143/6262/3760/776/3762/23630/489/6543/388591/1133
GO:0001505	regulation of neurotransmitter levels	27/551	5.58E-06	0.000537194	2572/5582/1268/140885/9162/815/6857/1806/140679/783/5864/9379/6809/6812/6505/776/27445/6855/6861/6616/9378/2644/285671/23576/148281/415117/6853
GO:0010721	negative regulation of cell development	26/551	5.62E-06	0.000537194	6496/57715/1745/1903/29116/340533/27124/6653/3670/1746/25791/7070/80031/4882/7143/23108/359845/9037/9241/9856/1620/3479/10371/2309/22891/5530
GO:0007214	gamma-aminobutyric acid signaling pathway	7/551	6.05E-06	0.000564154	2561/2558/2554/2562/2567/23349/2556
GO:0010976	positive regulation of neuron projection development	23/551	6.25E-06	0.000569843	57611/29993/1268/114798/10154/6792/51761/6857/57554/57699/22849/57698/163404/8536/9037/85358/8507/91584/9148/257194/816/1272/8437
GO:0098693	regulation of synaptic vesicle cycle	14/551	7.48E-06	0.000667019	5582/1268/9162/815/6857/783/5575/5864/6812/776/6855/5533/9378/6853
GO:0097479	synaptic vesicle localization	17/551	9.09E-06	0.000793026	5582/1268/9162/815/6857/783/5864/6809/6812/776/27445/6855/6861/6616/148281/415117/6853
GO:0032412	regulation of ion transmembrane transporter activity	21/551	9.72E-06	0.000820566	59283/7881/2257/5923/729956/2915/5581/3746/783/482/88/85358/6262/776/3747/23630/389816/4318/729993/388591/57338
GO:0001764	neuron migration	16/551	9.82E-06	0.000820566	9201/460/8828/340533/6792/815/6656/4929/84623/57698/9856/10371/23768/26468/816/23314
GO:0060384	innervation	7/551	1.06E-05	0.000864726	2561/84570/5582/2558/2562/3670/10371
GO:0051961	negative regulation of nervous system development	24/551	1.09E-05	0.000864726	6496/57715/1745/29116/340533/27124/6653/3670/1746/25791/7070/80031/51286/7143/23108/9037/9241/9856/1620/10371/2309/22891/5567/5530
GO:0007411	axon guidance	22/551	1.10E-05	0.000864726	9369/57715/8013/5293/8828/10154/3670/57731/1749/80031/5578/4917/7143/9037/9241/85358/91584/6710/10371/23768/5909/9378
GO:0032409	regulation of transporter activity	22/551	1.17E-05	0.000884234	59283/7881/2257/5923/729956/2915/5581/3746/783/482/9143/88/85358/6262/776/3747/23630/389816/4318/729993/388591/57338
GO:0097485	neuron projection guidance	22/551	1.17E-05	0.000884234	9369/57715/8013/5293/8828/10154/3670/57731/1749/80031/5578/4917/7143/9037/9241/85358/91584/6710/10371/23768/5909/9378
GO:0022898	regulation of transmembrane transporter activity	21/551	1.51E-05	0.001124077	59283/7881/2257/5923/729956/2915/5581/3746/783/482/88/85358/6262/776/3747/23630/389816/4318/729993/388591/57338
GO:0021872	forebrain generation of neurons	10/551	1.81E-05	0.001278135	1745/8828/6656/1746/1749/85358/10371/26468/27164/23314
GO:0086014	atrial cardiac muscle cell action potential	6/551	1.85E-05	0.001278135	783/6262/3760/3762/23630/388591
GO:0086026	atrial cardiac muscle cell to AV node cell signaling	6/551	1.85E-05	0.001278135	783/6262/3760/3762/23630/388591
GO:0086066	atrial cardiac muscle cell to AV node cell communication	6/551	1.85E-05	0.001278135	783/6262/3760/3762/23630/388591
GO:0007612	learning	15/551	2.07E-05	0.00138507	9369/6310/57144/2558/3274/9162/2915/9379/7143/85358/6529/6616/9378/6543/57338
GO:0030534	adult behavior	15/551	2.07E-05	0.00138507	9369/8013/53616/2257/114798/57731/2743/4929/9379/51286/680/85358/9378/7111/285671
GO:0051650	establishment of vesicle localization	23/551	2.12E-05	0.001392256	5871/5582/1780/1268/4641/9162/815/6857/783/55638/5864/6809/6812/1001/776/27445/6855/6861/7039/6616/148281/415117/6853
GO:0098661	inorganic anion transmembrane transport	13/551	2.39E-05	0.001543495	2561/2558/2554/2562/2915/2743/84561/2567/10861/54020/6505/266675/2556

GO:2000300	regulation of synaptic vesicle exocytosis	11/551	2.42E-05	0.001543495	5582/1268/9162/815/6857/783/5864/6812/776/6855/6853
GO:0061337	cardiac conduction	13/551	2.64E-05	0.00165358	6546/57731/783/482/4882/6262/3760/776/3762/23630/489/6543/388591
GO:2001257	regulation of cation channel activity	16/551	2.81E-05	0.001732808	59283/7881/2257/5923/729956/3746/783/88/85358/3747/23630/389816/4318/729993/388591/57338
GO:0044331	cell-cell adhesion mediated by cadherin	8/551	2.85E-05	0.001732808	340533/54825/1012/1008/1007/1016/1001/10076
GO:0043524	negative regulation of neuron apoptotic process	15/551	3.40E-05	0.00203298	1745/2561/8013/5582/2558/2562/3670/4929/116986/6620/2898/6812/666/55532/54463
GO:0050768	negative regulation of neurogenesis	22/551	3.48E-05	0.002051414	6496/57715/1745/29116/340533/27124/6653/3670/1746/25791/7070/80031/7143/23108/9037/9241/9856/1620/10371/2309/22891/5530
GO:0086019	cell-cell signaling involved in cardiac conduction	7/551	3.53E-05	0.002053977	783/6262/3760/776/3762/23630/388591
GO:0099171	presynaptic modulation of chemical synaptic transmission	5/551	3.69E-05	0.002116663	5582/1268/6857/783/776
GO:0086065	cell communication involved in cardiac conduction	9/551	3.77E-05	0.002129831	6546/783/482/6262/3760/776/3762/23630/388591
GO:0050679	positive regulation of epithelial cell proliferation	18/551	3.89E-05	0.002168019	1750/8013/8828/7423/2254/1749/4609/5578/1012/650/26051/9037/9241/1001/3479/7039/284361/7857
GO:0007156	homophilic cell adhesion via plasma membrane adhesion molecules	16/551	4.08E-05	0.002244395	56098/56127/64881/84966/57717/54825/84623/56147/56134/1012/1008/23705/1007/56097/1016/1001
GO:1902803	regulation of synaptic vesicle transport	11/551	4.32E-05	0.002340029	5582/1268/9162/815/6857/783/5864/6812/776/6855/6853
GO:0031629	synaptic vesicle fusion to presynaptic active zone membrane	6/551	4.46E-05	0.002385435	6857/5864/6809/6812/6616/415117
GO:0060047	heart contraction	20/551	4.55E-05	0.002401142	6546/2257/7423/57731/783/4882/6262/6444/3760/6505/776/3762/10371/4625/1839/7067/23630/489/6543/388591
GO:0051648	vesicle localization	23/551	4.91E-05	0.00255747	5871/5582/1780/1268/4641/9162/815/6857/783/55638/5864/6809/6812/1001/776/27445/6855/6861/7039/6616/148281/415117/6853
GO:1902476	chloride transmembrane transport	11/551	5.37E-05	0.002762032	2561/2558/2554/2562/2743/84561/2567/10861/6505/266675/2556
GO:0030048	actin filament-based movement	14/551	5.59E-05	0.002840134	2257/4641/644150/783/57698/88/6262/3760/776/3762/4625/7111/23630/388591
GO:0099500	vesicle fusion to plasma membrane	6/551	5.80E-05	0.002871217	6857/5864/6809/6812/6616/415117
GO:1901018	positive regulation of potassium ion transmembrane transporter activity	6/551	5.80E-05	0.002871217	3746/482/88/3747/389816/388591
GO:0017158	regulation of calcium ion-dependent exocytosis	12/551	6.21E-05	0.003037344	5582/1268/9162/815/6857/783/5864/6812/776/6855/6861/6853
GO:0021700	developmental maturation	21/551	6.70E-05	0.003238327	6496/5414/57731/84966/4929/64131/55638/650/5864/51286/4882/359845/6812/1001/6855/3479/9148/2309/9378/26468/816
GO:0003015	heart process	20/551	7.14E-05	0.003410982	6546/2257/7423/57731/783/4882/6262/6444/3760/6505/776/3762/10371/4625/1839/7067/23630/489/6543/388591
GO:0007626	locomotory behavior	17/551	7.35E-05	0.003467312	4886/53616/460/2257/2119/57144/2915/57731/5581/2743/4929/51286/7143/111/6616/7111/257194
GO:0006821	chloride transport	12/551	8.21E-05	0.003823763	2561/2558/2554/2562/2915/2743/84561/2567/10861/6505/266675/2556
GO:0021536	diencephalon development	10/551	8.29E-05	0.003823763	6496/8828/3670/4929/3746/650/9037/9241/3747/10371
GO:0008016	regulation of heart contraction	18/551	9.27E-05	0.004177971	6546/57731/783/482/4882/6262/3760/6505/776/3762/10371/4625/1839/7067/23630/489/6543/388591
GO:0045665	negative regulation of neuron differentiation	18/551	9.27E-05	0.004177971	6496/57715/1745/29116/27124/3670/1746/25791/7070/80031/7143/23108/9037/9856/10371/2309/22891/5530
GO:0048169	regulation of long-term neuronal synaptic plasticity	6/551	9.43E-05	0.004201685	2915/2898/85358/6855/9148/816
GO:1901016	regulation of potassium ion transmembrane transporter activity	9/551	9.79E-05	0.00431546	7881/3746/482/88/776/3747/23630/389816/388591
GO:0097120	receptor localization to synapse	8/551	0.000105195	0.004587413	59283/10082/57554/57576/1740/6809/23096/6616
GO:0035418	protein localization to synapse	9/551	0.000125416	0.005410406	10082/57554/1740/9379/6809/23096/27445/6616/9378
GO:0031346	positive regulation of cell projection organization	25/551	0.000136388	0.005821147	57611/29993/1268/114798/10154/6792/51761/6857/57554/57699/22849/57698/163404/8536/9750/9037/85358/8507/91584/89795/9148/257194/816/1272/8437
GO:2000302	positive regulation of synaptic vesicle exocytosis	4/551	0.000141885	0.005983083	1268/6857/783/776
GO:0071625	vocalization behavior	5/551	0.000143164	0.005983083	9369/9379/85358/1620/9378
GO:0070252	actin-mediated cell contraction	12/551	0.000151573	0.006269185	2257/783/57698/88/6262/3760/776/3762/4625/7111/23630/388591
GO:0071526	semaphorin-plexin signaling pathway	7/551	0.000159196	0.006451443	57715/8828/10154/80031/9037/91584/10371
GO:1901381	positive regulation of potassium ion transmembrane transport	7/551	0.000159196	0.006451443	3746/482/88/3747/23630/389816/388591
GO:0071241	cellular response to inorganic substance	17/551	0.000162472	0.006518379	84557/2308/2983/6857/57699/1740/131034/84329/10235/3747/55532/6861/30061/4318/148281/3756/8437
GO:0098656	anion transmembrane transport	20/551	0.000165194	0.00656196	2561/8604/2558/2554/2562/9194/2915/2743/140679/84561/2567/8671/10861/23428/54020/6505/266675/6508/2556/6534
GO:0021537	telencephalon development	19/551	0.000170488	0.006705873	6496/6546/1745/8013/8828/1746/1749/140679/84623/650/3362/7143/85358/5915/91584/10371/26468/27164/152789
GO:0002027	regulation of heart rate	11/551	0.000175325	0.00682917	6546/57731/783/6262/3760/6505/776/3762/10371/4625/23630
GO:0050919	negative chemotaxis	7/551	0.000187094	0.007217518	57715/8828/80031/9037/91584/10371/23768
GO:1904062	regulation of cation transmembrane transport	22/551	0.000196455	0.007506438	59283/6546/7881/2257/5923/729956/5581/3746/783/7070/482/88/85358/6262/776/3747/23630/389816/4318/729993/388591/57338
GO:0099624	atrial cardiac muscle cell membrane repolarization	4/551	0.000217716	0.008163331	3760/776/3762/23630
GO:1902805	positive regulation of synaptic vesicle transport	4/551	0.000217716	0.008163331	1268/6857/783/776
GO:0086002	cardiac muscle cell action potential involved in contraction	8/551	0.000238021	0.008842026	2257/783/6262/3760/776/3762/23630/388591
GO:0051968	positive regulation of synaptic transmission, glutamatergic	6/551	0.000265618	0.009776709	59283/57616/7143/6812/85358/9378
GO:0015698	inorganic anion transport	15/551	0.000276837	0.010097015	2561/2558/2554/2562/2915/2743/84561/2567/8671/10861/54020/6505/266675/6508/2556
GO:0099173	postsynapse organization	14/551	0.000296288	0.010709071	8828/2119/729956/6792/25791/2743/26045/1740/9118/9379/85358/9378/22891/816
GO:0046928	regulation of neurotransmitter secretion	11/551	0.000320759	0.011490058	5582/1268/9162/815/6857/783/5864/6812/776/6855/6853
GO:1990138	neuron projection extension	14/551	0.000336895	0.011752674	57715/9201/57611/8828/6792/6857/57699/80031/57698/7143/9037/91584/10371/8437
GO:0021954	central nervous system neuron development	9/551	0.000342171	0.011752674	9201/8828/6656/57731/4929/91584/10371/26468/27164
GO:0086001	cardiac muscle cell action potential	9/551	0.000342171	0.011752674	6546/2257/783/6262/3760/776/3762/23630/388591
GO:0007215	glutamate receptor signaling pathway	10/551	0.000342705	0.011752674	2891/59283/5923/729956/2915/2898/85358/2899/729993/23732
GO:1903522	regulation of blood circulation	19/551	0.000342738	0.011752674	6546/3274/57731/783/482/4882/6262/3760/6505/776/3762/10371/4625/1839/7067/23630/489/6543/388591
GO:1901215	negative regulation of neuron death	16/551	0.000356506	0.012121207	1745/2561/8013/5582/2558/2562/6653/3670/4929/116986/6620/2898/6812/666/55532/54463
GO:0043523	regulation of neuron apoptotic process	16/551	0.00037678	0.012702872	1745/2561/8013/5582/2558/2562/3670/4929/116986/6620/2898/6812/666/55532/2309/54463
GO:0001956	positive regulation of neurotransmitter secretion	5/551	0.000398685	0.013329382	1268/6857/783/6812/776
GO:0051592	response to calcium ion	13/551	0.000433064	0.014359107	8604/6857/57699/131034/10235/6262/6861/6529/148281/5530/3756/8437/311
GO:0043268	positive regulation of potassium ion transport	7/551	0.000448613	0.014671967	3746/482/88/3747/23630/389816/388591
GO:1902285	semaphorin-plexin signaling pathway involved in neuron projection guidance	4/551	0.000449814	0.014671967	8828/10154/91584/10371
GO:0021675	nerve development	9/551	0.000461592	0.014815245	2561/84570/5582/8828/2558/2562/3670/91584/10371
GO:0060291	long-term synaptic potentiation	9/551	0.000461592	0.014815245	57616/729956/26045/10590/6809/7143/85358/6616/6543
GO:0010977	negative regulation of neuron projection development	13/551	0.000493529	0.015472901	57715/29116/27124/25791/7070/80031/7143/23108/9037/9856/10371/22891/5530
GO:0010996	response to auditory stimulus	5/551	0.000497044	0.015472901	51761/3746/9379/85358/9378
GO:0021895	cerebral cortex neuron differentiation	5/551	0.000497044	0.015472901	1745/1746/81544/26038/26468
GO:0098742	cell-cell adhesion via plasma-membrane adhesion molecules	19/551	0.000497509	0.015472901	56098/114798/56127/64881/84966/57717/54825/84623/56147/56134/1012/650/1008/23705/1007/56097/1016/1001/9378
GO:0016339	calcium-dependent cell-cell adhesion via plasma membrane cell adhesion molecules	7/551	0.000511905	0.015798174	56127/57717/1012/1008/1007/1016/1001
GO:0007613	memory	11/551	0.000516019	0.015803567	6310/1268/57144/5923/3274/729956/22849/85358/1620/6543/57338
GO:1903539	protein localization to postsynaptic membrane	6/551	0.000529355	0.016089175	10082/57554/1740/6809/23096/6616
GO:0009190	cyclic nucleotide biosynthetic process	5/551	0.000612591	0.018205289	108/2983/4882/111/2977
GO:0052652	cyclic purine nucleotide metabolic process	5/551	0.000612591	0.018205289	108/2983/4882/111/2977
GO:0060074	synapse maturation	5/551	0.000612591	0.018205289	84966/55638/9148/9378/816
GO:0001508	action potential	12/551	0.000632433	0.018656786	6546/2257/1268/783/2898/6262/57369/3760/776/3762/23630/388591
GO:0050796	regulation of insulin secretion	14/551	0.000655914	0.019208234	1268/3670/5581/5019/10411/5578/55638/111/7447/776/3747/6616/5530/773
GO:0099072	regulation of postsynaptic membrane neurotransmitter receptor levels	8/551	0.000669292	0.019457957	59283/10082/57554/1740/6809/23096/6616/23732
GO:0032535	regulation of cellular component size	23/551	0.000689437	0.01989942	57715/57611/6792/4641/57731/5581/644150/84561/54825/29767/80031/57698/7143/23108/9037/88/343578/85358/91584/6710/10371/7111/8800
GO:0048675	axon extension	11/551	0.000694859	0.019912675	57715/9201/57611/8828/6792/80031/57698/7143/9037/91584/10371

GO:0048846	axon extension involved in axon guidance	6/551	0.000720928	0.020368762	57715/8828/80031/9037/91584/10371
GO:1902284	neuron projection extension involved in neuron projection guidance	6/551	0.000720928	0.020368762	57715/8828/80031/9037/91584/10371
GO:0021544	subpallium development	5/551	0.000747147	0.020816353	1745/1746/85358/5915/152789
GO:0045956	positive regulation of calcium ion-dependent exocytosis	5/551	0.000747147	0.020816353	1268/6857/783/6812/776
GO:1903818	positive regulation of voltage-gated potassium channel activity	4/551	0.000818939	0.022411075	3746/3747/389816/388591
GO:0048663	neuron fate commitment	8/551	0.000821144	0.022411075	23040/1745/116448/3670/6656/1746/5396/23314
GO:0086003	cardiac muscle cell contraction	8/551	0.000821144	0.022411075	2257/783/6262/3760/776/3762/23630/388591
GO:0043270	positive regulation of ion transport	18/551	0.000837051	0.022690865	2257/51761/815/3746/783/7070/482/6812/88/85358/6262/776/3747/6529/23630/389816/1272/388591
GO:0008015	blood circulation	28/551	0.000869295	0.023406779	6546/4886/2257/1268/7423/3274/2983/57731/783/4882/680/91608/6262/6444/3760/6505/776/1827/3762/10371/4625/1839/7067/23630/489/23576/6543/388591
GO:0050805	negative regulation of synaptic transmission	8/551	0.000906809	0.024254128	9162/2898/7143/6812/85358/2899/56853/6529
GO:0050803	regulation of synapse structure or activity	16/551	0.000952774	0.02490323	139065/8828/2119/114798/729956/6792/25791/347731/26045/8536/55638/85358/9148/23768/9378/816
GO:0045445	myoblast differentiation	9/551	0.000955168	0.02490323	7881/3670/9750/389072/10324/3479/126272/6665/4217
GO:0001941	postsynaptic membrane organization	6/551	0.000962114	0.02490323	2119/729956/2743/9379/85358/9378
GO:0051590	positive regulation of neurotransmitter transport	6/551	0.000962114	0.02490323	1268/6857/783/6812/776/6529
GO:2001222	regulation of neuron migration	6/551	0.000962114	0.02490323	340533/815/57698/10371/23768/816
GO:0031345	negative regulation of cell projection organization	14/551	0.000970163	0.024950598	57715/29116/27124/25791/7070/80031/7143/23108/9037/9856/10371/5909/22891/5530
GO:0051588	regulation of neurotransmitter transport	12/551	0.001060401	0.027097643	5582/1268/9162/815/6857/783/5864/6812/776/6855/6529/6853
GO:0032411	positive regulation of transporter activity	10/551	0.001079856	0.027420143	3746/783/482/9143/88/85358/6262/3747/389816/388591
GO:0003013	circulatory system process	28/551	0.001108637	0.0279739	6546/4886/2257/1268/7423/3274/2983/57731/783/4882/680/91608/6262/6444/3760/6505/776/1827/3762/10371/4625/1839/7067/23630/489/23576/6543/388591
GO:0010038	response to metal ion	22/551	0.001152425	0.028611879	84557/8604/6857/3746/6620/57699/1740/131034/84329/10235/6262/3747/55532/6861/6529/30061/4318/148281/5530/3756/8437/311
GO:0030073	insulin secretion	15/551	0.001158457	0.028611879	1268/3670/5581/5019/10411/5578/55638/111/7447/776/3747/27445/6616/5530/773
GO:0090276	regulation of peptide hormone secretion	15/551	0.001158457	0.028611879	1268/3670/5581/5019/10411/5578/55638/111/91608/7447/776/3747/6616/5530/773
GO:0050905	neuromuscular process	10/551	0.001162447	0.028611879	8013/2257/51761/2743/7143/111/4861/9378/3756/57338
GO:0017157	regulation of exocytosis	15/551	0.001217039	0.029772918	5582/5414/1268/9162/815/6857/783/5864/6812/7447/776/27445/6855/6861/6853
GO:0060999	positive regulation of dendritic spine development	6/551	0.001260956	0.030576954	6792/22849/8536/85358/9148/816
GO:0071248	cellular response to metal ion	14/551	0.001265148	0.030576954	84557/6857/57699/1740/131034/84329/10235/55532/6861/30061/4318/148281/3756/8437
GO:0007263	nitric oxide mediated signal transduction	5/551	0.00128398	0.030846279	51655/2983/3747/2977/23576
GO:0007416	synapse assembly	13/551	0.001360697	0.032330019	139065/114798/56127/57717/347731/26045/84623/9379/1007/85358/27445/23768/9378
GO:0099509	regulation of presynaptic cytosolic calcium ion concentration	4/551	0.001361858	0.032330019	1268/10590/783/776
GO:0009187	cyclic nucleotide metabolic process	6/551	0.001434646	0.033531041	108/5141/2983/4882/111/2977
GO:0060996	dendritic spine development	9/551	0.001437522	0.033531041	6792/815/25791/22849/8536/85358/9148/22891/816
GO:1901379	regulation of potassium ion transmembrane transport	9/551	0.001437522	0.033531041	7881/3746/482/88/776/3747/23630/389816/388591
GO:0006929	substrate-dependent cell migration	5/551	0.001513924	0.034769598	8828/752/57698/10371/6591
GO:0021884	forebrain neuron development	5/551	0.001513924	0.034769598	8828/6656/10371/26468/27164
GO:0051402	neuron apoptotic process	16/551	0.00151662	0.034769598	1745/2561/8013/5582/2558/2562/3670/4929/116986/6620/2898/6812/666/55532/2309/54463
GO:0030516	regulation of axon extension	9/551	0.001554146	0.035427465	57715/57611/6792/80031/57698/7143/9037/91584/10371
GO:0006941	striated muscle contraction	13/551	0.00160109	0.036291368	6546/2257/7423/51761/783/6262/3760/776/10324/3762/4625/23630/388591
GO:1903305	regulation of regulated secretory pathway	12/551	0.001610561	0.036300955	5582/1268/9162/815/6857/783/5864/6812/776/6855/6861/6853
GO:0043931	ossification involved in bone maturation	4/551	0.001710056	0.038115244	64131/650/359845/3479
GO:0097107	postsynaptic density assembly	4/551	0.001710056	0.038115244	26045/9379/85358/9378
GO:0051656	establishment of organelle localization	27/551	0.001752677	0.038849403	8013/5871/5582/5293/1780/1268/3834/4641/9162/815/6857/783/55638/5864/6809/6812/1001/776/27445/6855/6861/7039/6616/79998/148281/415117/6853
GO:0099565	chemical synaptic transmission, postsynaptic	10/551	0.001773062	0.039085293	2891/2562/9162/2743/2898/85358/56853/9378/5530/642938
GO:0050807	regulation of synapse organization	15/551	0.001952906	0.042814532	139065/8828/2119/114798/729956/6792/25791/347731/26045/8536/85358/9148/23768/9378/816
GO:0003407	neural retina development	7/551	0.001975622	0.043077151	6496/1745/51761/1746/7070/6604/84440
GO:0098915	membrane repolarization during ventricular cardiac muscle cell action potential	4/551	0.002115522	0.045564234	3760/3762/23630/388591
GO:1902170	cellular response to reactive nitrogen species	4/551	0.002115522	0.045564234	2308/2983/3747/4217
GO:0060048	cardiac muscle contraction	11/551	0.002123757	0.045564234	6546/2257/7423/783/6262/3760/776/3762/4625/23630/388591
GO:0010959	regulation of metal ion transport	22/551	0.002136796	0.045600141	6546/7881/2257/815/57731/5581/3746/783/7070/482/154215/88/6262/776/3747/55532/23630/389816/816/1272/388591/57338
GO:0031333	negative regulation of protein complex assembly	11/551	0.002253204	0.047643927	27124/6653/57731/29767/6812/29103/6710/7111/24139/7067/51339
GO:0032414	positive regulation of ion transmembrane transporter activity	9/551	0.002256318	0.047643927	3746/783/482/88/85358/6262/3747/389816/388591
GO:0032228	regulation of synaptic transmission, GABAergic	5/551	0.002385542	0.049188449	1268/5581/6812/6529/23349
GO:0032594	protein transport within lipid bilayer	5/551	0.002385542	0.049188449	59283/23180/57554/6809/6616
GO:0048841	regulation of axon extension involved in axon guidance	5/551	0.002385542	0.049188449	57715/80031/9037/91584/10371
GO:0097106	postsynaptic density organization	5/551	0.002385542	0.049188449	26045/1740/9379/85358/9378
GO:0048857	neural nucleus development	7/551	0.002390765	0.049188449	57447/2254/3746/84623/9118/9143/3747
GO:1901879	regulation of protein depolymerization	8/551	0.002407926	0.049288764	57731/29767/9037/25876/88/6710/89795/7111
GO:0097060	synaptic membrane	62/585	3.06E-25	1.47E-22	2891/9890/59283/6546/2561/3765/53616/2572/401190/5582/8828/1268/2558/114798/2554/2562/729956/80758/9162/2915/339453/815/6857/57554/2743/84966/347731/26045/7732/3746/22849/1740/2567/1008/6809/2898/6812/3738/1007/88/55450/85358/2899/3760/776/3747/6855/9148/6529/79870/6616/9378/148281/415117/1272/23345/642938/729993/3756/58512/2556/1133
GO:0045211	postsynaptic membrane	47/585	1.30E-19	3.12E-17	2891/9890/59283/6546/2561/53616/401190/8828/2558/114798/2554/2562/729956/80758/9162/2915/815/57554/2743/84966/347731/26045/7732/3746/22849/1740/2567/1008/2898/3738/1007/88/55450/85358/2899/3747/9148/6529/79870/1272/23345/642938/729993/3756/58512/2556/1133
GO:0099240	intrinsic component of synaptic membrane	28/585	8.87E-14	1.42E-11	9890/6546/2561/3765/53616/401190/8828/1268/2558/114798/2554/729956/347731/26045/3746/2567/1008/3738/1007/3760/776/6529/9378/148281/1272/729993/3756/2556
GO:0099572	postsynaptic specialization	39/585	5.57E-13	6.47E-11	9890/59283/2561/9201/53616/401190/5582/2558/114798/2554/729956/80758/6792/9162/2915/815/57554/347731/26045/5662/7732/22849/26115/1740/8536/1008/2898/88/55450/85358/27445/9148/79870/642938/729993/3756/6853/58512/2556
GO:0099699	integral component of synaptic membrane	26/585	6.73E-13	6.47E-11	9890/6546/2561/3765/53616/8828/1268/2558/114798/2554/729956/347731/26045/3746/2567/1008/3738/1007/3760/776/6529/9378/148281/729993/3756/2556
GO:0042734	presynaptic membrane	26/585	1.95E-12	1.56E-10	3765/2572/401190/1268/2558/9162/6857/84966/3746/1008/6809/2898/6812/3738/1007/2899/3760/776/3747/6855/6529/6616/9378/415117/1272/3756
GO:0034702	ion channel complex	35/585	6.01E-12	4.13E-10	2891/59283/2561/7881/2558/2554/2562/729956/57554/2743/23415/3746/783/1740/2567/9312/3739/2898/3738/3754/85358/6262/2899/3760/776/3747/3762/266675/6616/23630/389816/729993/3756/773/2556
GO:1902495	transmembrane transporter complex	36/585	1.24E-11	6.17E-10	2891/59283/2561/7881/2558/2554/2562/729956/57554/2743/23415/3746/783/1740/2567/482/9312/3739/2898/3738/3754/85358/6262/2899/3760/776/3747/3762/266675/6616/23630/389816/729993/3756/773/2556
GO:0098793	presynapse	45/585	1.25E-11	6.17E-10	3765/2572/29993/401190/5582/5414/1268/2558/9162/2983/6857/84966/132204/140679/7732/10590/3746/6620/5864/1008/9379/6809/2898/9143/6812/3738/1007/2899/3760/776/3747/27445/6855/6861/6529/5533/30061/6616/9378/388662/148281/415117/1272/3756/6853
GO:0098984	neuron to neuron synapse	37/585	1.28E-11	6.17E-10	9890/59283/9201/53616/401190/5582/114798/9465/729956/80758/6792/9162/2915/815/6857/57554/347731/26045/5662/7732/22849/26115/1740/8536/5575/2898/88/55450/85358/27445/9148/79870/642938/729993/3756/6853/58512
GO:1990351	transporter complex	36/585	2.50E-11	1.09E-09	2891/59283/2561/7881/2558/2554/2562/729956/57554/2743/23415/3746/783/1740/2567/482/9312/3739/2898/3738/3754/85358/6262/2899/3760/776/3747/3762/266675/6616/23630/389816/729993/3756/773/2556
GO:0014069	postsynaptic density	34/585	1.03E-10	4.12E-09	9890/59283/9201/53616/401190/5582/114798/729956/80758/6792/9162/2915/815/57554/347731/26045/5662/7732/22849/26115/1740/8536/2898/88/55450/85358/27445/9148/79870/642938/729993/3756/6853/58512
GO:0032279	asymmetric synapse	34/585	1.44E-10	5.34E-09	9890/59283/9201/53616/401190/5582/114798/729956/80758/6792/9162/2915/815/57554/347731/26045/5662/7732/22849/26115/1740/8536/2898/88/55450/85358/27445/9148/79870/642938/729993/3756/6853/58512

GO:0098978	glutamatergic synapse	35/585	4.06E-10	1.40E-08	139065/9890/53616/401190/8828/1268/114798/10082/729956/80758/6792/9162/6857/347731/26045/26115/1740/8536/5575/1008/6809/2898/7143/6812/3738/88/2899/5533/6616/9378/388662/148281/5530/729993/58512
GO:0098982	GABA-ergic synapse	16/585	5.19E-10	1.61E-08	2561/1268/2558/114798/2554/2562/2743/26045/140679/2567/1012/1008/6529/9378/388662/2556
GO:0098936	intrinsic component of postsynaptic membrane	20/585	5.37E-10	1.61E-08	9890/6546/2561/53616/401190/8828/2558/114798/2554/729956/347731/26045/3746/1008/3738/1007/6529/1272/729993/2556
GO:0099634	postsynaptic specialization membrane	18/585	1.15E-09	3.24E-08	9890/59283/2561/53616/401190/2558/114798/2554/729956/9162/2915/347731/26045/1740/1008/88/729993/2556
GO:0034705	potassium channel complex	17/585	5.13E-09	1.37E-07	7881/23415/3746/1740/9312/3739/2898/3738/3754/2899/3760/3747/3762/6616/23630/389816/3756
GO:0034703	cation channel complex	25/585	9.54E-09	2.42E-07	2891/59283/7881/729956/23415/3746/783/1740/9312/3739/2898/3738/3754/6262/2899/3760/776/3747/3762/6616/23630/389816/729993/3756/773
GO:0099055	integral component of postsynaptic membrane	18/585	1.11E-08	2.67E-07	9890/6546/2561/53616/8828/2558/114798/2554/729956/347731/26045/3746/1008/3738/1007/6529/729993/2556
GO:0008076	voltage-gated potassium channel complex	15/585	6.20E-08	1.42E-06	7881/23415/3746/1740/9312/3739/3738/3754/3760/3747/3762/6616/23630/389816/3756
GO:0032589	neuron projection membrane	12/585	8.97E-08	1.89E-06	2558/2554/729956/4641/3746/7070/2567/9750/3747/729993/3756/2556
GO:0033267	axon part	32/585	9.04E-08	1.89E-06	7881/29993/5582/1268/23046/114787/57447/27124/57616/5923/6792/9162/57554/57731/25791/140679/3746/81544/29767/7070/1740/57698/55638/5864/6809/2898/3738/2899/3747/6855/6616/3756
GO:0098889	intrinsic component of presynaptic membrane	14/585	1.71E-07	3.42E-06	3765/401190/1268/2558/3746/1008/3738/1007/3760/776/6529/9378/1272/3756
GO:0150034	distal axon	26/585	3.22E-07	6.20E-06	29993/5582/1268/23046/114787/57447/27124/57616/5923/6792/9162/25791/140679/3746/81544/29767/7070/57698/5864/6809/2898/3738/2899/3747/6855/6616
GO:0098948	intrinsic component of postsynaptic specialization membrane	13/585	4.03E-07	7.45E-06	9890/2561/53616/401190/2558/114798/2554/729956/347731/26045/1008/729993/2556
GO:0048786	presynaptic active zone	12/585	8.08E-07	1.44E-05	9162/2983/140679/1008/6809/6812/776/27445/6855/9378/415117/6853
GO:0098685	Schaffer collateral - CA1 synapse	13/585	8.54E-07	1.47E-05	9162/2915/815/26045/9118/5575/6809/7143/23096/6855/9378/5530/6853
GO:0099060	integral component of postsynaptic specialization membrane	12/585	1.76E-06	2.91E-05	9890/2561/53616/2558/114798/2554/729956/347731/26045/1008/729993/2556
GO:0098839	postsynaptic density membrane	12/585	2.04E-06	3.10E-05	9890/59283/53616/401190/114798/9162/2915/347731/26045/1740/88/729993
GO:0099056	integral component of presynaptic membrane	12/585	2.04E-06	3.10E-05	3765/1268/2558/3746/1008/3738/1007/3760/776/6529/9378/3756
GO:0032590	dendrite membrane	9/585	2.06E-06	3.10E-05	2558/2554/729956/3746/7070/2567/3747/729993/2556
GO:0031256	leading edge membrane	18/585	2.60E-06	3.79E-05	29993/2558/5141/2554/729956/6792/4641/5662/3746/7070/2567/9750/10235/3747/729993/3756/54212/2556
GO:0070382	exocytic vesicle	19/585	7.16E-06	0.000101305	2572/5414/9162/6857/132204/140679/7732/5864/6809/9143/6855/3479/6861/30061/6616/388662/148281/415117/6853
GO:0008021	synaptic vesicle	18/585	7.52E-06	0.000103319	2572/5414/9162/6857/132204/140679/7732/5864/6809/9143/6855/6861/30061/6616/388662/148281/415117/6853
GO:1902711	GABA-A receptor complex	6/585	1.32E-05	0.000176235	2561/2558/2554/2562/2567/2556
GO:1902710	GABA receptor complex	6/585	1.84E-05	0.000238816	2561/2558/2554/2562/2567/2556
GO:0008328	ionotropic glutamate receptor complex	9/585	2.02E-05	0.000255682	2891/59283/729956/57554/1740/2898/85358/2899/729993
GO:0098878	neurotransmitter receptor complex	9/585	2.77E-05	0.000338397	2891/59283/729956/57554/1740/2898/85358/2899/729993
GO:0048787	presynaptic active zone membrane	7/585	2.81E-05	0.000338397	9162/1008/6809/6812/776/9378/415117
GO:0060076	excitatory synapse	8/585	7.74E-05	0.000907792	2891/3765/729956/9162/6857/26045/3760/6855
GO:0034707	chloride channel complex	8/585	0.000104494	0.001179534	2561/2558/2554/2562/2743/2567/266675/2556
GO:0099092	postsynaptic density, intracellular component	5/585	0.000105447	0.001179534	80758/6792/5662/26115/88
GO:0030426	growth cone	15/585	0.000126754	0.001385655	1268/23046/114787/57447/27124/57616/5923/6792/25791/81544/29767/7070/57698/6809/6616
GO:0030427	site of polarized growth	15/585	0.000144995	0.001549834	1268/23046/114787/57447/27124/57616/5923/6792/25791/81544/29767/7070/57698/6809/6616
GO:0030672	synaptic vesicle membrane	11/585	0.00022698	0.002322921	2572/6857/132204/140679/5864/9143/6855/6861/388662/148281/6853
GO:0099501	exocytic vesicle membrane	11/585	0.00022698	0.002322921	2572/6857/132204/140679/5864/9143/6855/6861/388662/148281/6853
GO:0099091	postsynaptic specialization, intracellular component	5/585	0.000245349	0.002458606	80758/6792/5662/26115/88
GO:0031252	cell leading edge	25/585	0.000290468	0.002848821	89846/29993/2558/5141/2554/27124/729956/6792/4641/5662/3746/10411/7070/2567/57698/9750/6809/10235/10324/9079/3747/729993/3756/54212/2556
GO:0043025	neuronal cell body	29/585	0.000296135	0.002848821	57795/5126/7881/460/2558/9162/57731/3746/57699/81544/7070/5137/57698/9312/2898/680/55450/2899/8507/3747/1620/6861/9148/9481/9378/26507/6543/3756/773
GO:0060198	clathrin-sculpted vesicle	4/585	0.000317535	0.002994793	2572/6857/140679/5864
GO:0043204	perikaryon	12/585	0.000357047	0.003302684	5126/7881/460/57699/57698/9312/2898/2899/3747/9148/6543/3756
GO:0098563	intrinsic component of synaptic vesicle membrane	7/585	0.000389342	0.003533465	6857/132204/140679/5864/6855/388662/6853
GO:0030673	axolemma	4/585	0.000815492	0.007263918	3746/7070/3747/3756
GO:0044306	neuron projection terminus	12/585	0.000871359	0.007464837	29993/5582/9162/6857/140679/3746/5864/2898/3738/2899/3747/6855
GO:0032809	neuronal cell body membrane	5/585	0.00089819	0.007464837	2558/3746/7070/9312/3747
GO:0044298	cell body membrane	5/585	0.00089819	0.007464837	2558/3746/7070/9312/3747
GO:0044304	main axon	8/585	0.000901006	0.007464837	7881/57554/57731/3746/7070/1740/3747/3756
GO:0043679	axon terminus	11/585	0.000915645	0.007464837	29993/5582/9162/140679/3746/5864/2898/3738/2899/3747/6855
GO:0099146	intrinsic component of postsynaptic density membrane	7/585	0.001049652	0.008414706	9890/53616/401190/114798/347731/26045/729993
GO:0031253	cell projection membrane	21/585	0.00110369	0.008702868	29993/2558/5141/2554/729956/6792/4641/5662/3746/54825/7070/2567/9750/10235/85358/3747/729993/5158/3756/54212/2556
GO:0030133	transport vesicle	23/585	0.001129167	0.008760148	5126/2572/5414/9162/6857/132204/140679/7732/10590/5864/6809/9143/6855/3479/6861/8906/7039/30061/6616/388662/148281/415117/6853
GO:0043197	dendritic spine	13/585	0.001277505	0.009753651	2891/6546/729956/9162/815/5662/3739/88/85358/9148/6543/5530/729993
GO:0044309	neuron spine	13/585	0.001426035	0.010717541	2891/6546/729956/9162/815/5662/3739/88/85358/9148/6543/5530/729993
GO:0016342	catenin complex	5/585	0.001506619	0.011148979	1012/1008/1007/1016/1001
GO:0043235	receptor complex	23/585	0.001865427	0.013595002	2891/59283/2561/8828/2558/2554/2562/729956/53353/10154/57554/51559/255743/1740/2567/650/2898/85358/2899/91584/7189/729993/2556
GO:0098686	hippocampal mossy fiber to CA3 synapse	5/585	0.002374172	0.017044429	9465/6857/26045/5575/2898
GO:0030285	integral component of synaptic vesicle membrane	5/585	0.002730671	0.019315485	6857/132204/140679/6855/388662
GO:0032839	dendrite cytoplasm	5/585	0.003124355	0.021779921	7881/6792/57576/2898/2899
GO:0030658	transport vesicle membrane	14/585	0.003270573	0.022473509	2572/6857/132204/140679/10590/5864/9143/6855/6861/8906/7039/388662/148281/6853
GO:0002116	semaphorin receptor complex	3/585	0.003618179	0.024511889	8828/10154/91584
GO:0030315	T-tubule	6/585	0.003911518	0.025773151	59283/6546/783/3760/776/3762
GO:0099061	integral component of postsynaptic density membrane	6/585	0.003911518	0.025773151	9890/53616/114798/347731/26045/729993
GO:1990454	L-type voltage-gated calcium channel complex	3/585	0.004718394	0.030549094	59283/783/776
GO:0031225	anchored component of membrane	12/585	0.004763372	0.030549094	2572/4978/401190/10082/84966/503542/7070/1012/5864/257194/1272/6853
GO:0033017	sarcoplasmic reticulum membrane	5/585	0.005724467	0.036229851	6262/10324/816/489/57338
GO:0032281	AMPA glutamate receptor complex	4/585	0.007905841	0.04938584	2891/59283/729956/729993
GO:0046873	metal ion transmembrane transporter activity	43/544	3.24E-11	2.33E-08	59283/6546/3765/7881/1268/81894/27133/23415/3746/84561/783/482/57582/9312/8671/3739/2898/3738/3754/6262/2899/3760/6505/776/3747/55532/3762/6529/30061/6616/23630/389816/388662/489/6543/84102/3756/773/6553/56660/57338/6534/57185
GO:0022843	voltage-gated cation channel activity	23/544	8.02E-11	2.88E-08	59283/3765/7881/1268/27133/23415/3746/783/57582/9312/3739/84329/3738/3754/3760/776/3747/3762/6616/23630/389816/3756/773
GO:0022839	ion gated channel activity	36/544	1.37E-10	3.29E-08	2891/59283/2561/3765/7881/1268/2558/2554/2562/2743/27133/23415/3746/783/2567/57582/9312/3739/84329/2898/3738/3754/6262/2899/3760/776/3747/3762/6616/23630/389816/3756/773/56660/57338/2556
GO:0022836	gated channel activity	36/544	2.84E-10	5.10E-08	2891/59283/2561/3765/7881/1268/2558/2554/2562/2743/27133/23415/3746/783/2567/57582/9312/3739/84329/2898/3738/3754/6262/2899/3760/776/3747/3762/6616/23630/389816/3756/773/56660/57338/2556
GO:0015079	potassium ion transmembrane transporter activity	23/544	8.03E-10	1.16E-07	3765/7881/27133/23415/3746/84561/482/57582/9312/3739/2898/3738/3754/2899/3760/3747/3762/6616/23630/389816/3756/6553/56660
GO:0005249	voltage-gated potassium channel activity	17/544	1.31E-09	1.35E-07	3765/7881/27133/23415/3746/57582/9312/3739/3738/3754/3760/3747/3762/6616/23630/389816/3756
GO:0005267	potassium channel activity	20/544	1.32E-09	1.35E-07	3765/7881/27133/23415/3746/57582/9312/3739/2898/3738/3754/2899/3760/3747/3762/6616/23630/389816/3756/56660
GO:0005216	ion channel activity	38/544	5.07E-09	4.56E-07	2891/59283/2561/3765/7881/1268/2558/2554/2562/2743/27133/23415/3746/783/2567/57582/9312/3739/84329/2898/3738/3754/6262/2899/3760/776/3747/3762/266675/30061/6616/23630/389816/3756/773/56660/57338/2556
GO:0005244	voltage-gated ion channel activity	24/544	1.24E-08	8.48E-07	59283/3765/7881/1268/27133/23415/3746/783/57582/9312/3739/84329/3738/3754/3760/776/3747/3762/6616/23630/389816/3756/773/56660
GO:0022832	voltage-gated channel activity	24/544	1.24E-08	8.48E-07	59283/3765/7881/1268/27133/23415/3746/783/57582/9312/3739/84329/3738/3754/3760/776/3747/3762/6616/23630/389816/3756/773/56660
GO:0022838	substrate-specific channel activity	38/544	1.30E-08	8.48E-07	2891/59283/2561/3765/7881/1268/2558/2554/2562/2743/27133/23415/3746/783/2567/57582/9312/3739/84329/2898/3738/3754/6262/2899/3760/776/3747/3762/266675/30061/6616/23630/389816/3756/773/56660/57338/2556

GO:0015267	channel activity	39/544	1.84E-08	1.08E-06	2891/59283/2561/3765/7881/1268/2558/2554/2562/2743/27133/23415/3746/783/2567/57582/9312/3739/84329/2898/3738/3754/6262/2899/57369/3760/776/3747/3762/266675/30061/6616/23630/389816/3756/773/56660/57338/2556
GO:0022803	passive transmembrane transporter activity	39/544	1.96E-08	1.08E-06	2891/59283/2561/3765/7881/1268/2558/2554/2562/2743/27133/23415/3746/783/2567/57582/9312/3739/84329/2898/3738/3754/6262/2899/57369/3760/776/3747/3762/266675/30061/6616/23630/389816/3756/773/56660/57338/2556
GO:0005261	cation channel activity	30/544	6.88E-08	3.53E-06	2891/59283/3765/7881/1268/27133/23415/3746/783/57582/9312/3739/84329/2898/3738/3754/6262/2899/3760/776/3747/3762/30061/6616/23630/389816/3756/773/56660/57338
GO:0015077	monovalent inorganic cation transmembrane transporter activity	33/544	1.45E-07	6.96E-06	6546/3765/7881/140679/27133/23415/3746/84561/482/57582/9312/8671/3739/84329/2898/3738/3754/2899/3760/6505/3747/3762/6529/6616/23630/389816/388662/489/6543/3756/6553/56660/6534
GO:0030594	neurotransmitter receptor activity	15/544	3.14E-06	0.000141027	2891/2561/2558/2554/2562/3274/2915/2743/2567/3362/2898/2899/3354/2556/1133
GO:0005237	inhibitory extracellular ligand-gated ion channel activity	6/544	5.16E-06	0.000218312	2561/2558/2554/2743/2567/2556
GO:0098960	postsynaptic neurotransmitter receptor activity	9/544	1.14E-05	0.000453378	2561/2558/2554/2562/2915/2743/2567/2898/2556
GO:0022824	transmitter-gated ion channel activity	10/544	1.58E-05	0.000528109	2891/2561/2558/2554/2562/2743/2567/2898/2899/2556
GO:0022835	transmitter-gated channel activity	10/544	1.58E-05	0.000528109	2891/2561/2558/2554/2562/2743/2567/2898/2899/2556
GO:0004890	GABA-A receptor activity	6/544	1.62E-05	0.000528109	2561/2558/2554/2562/2567/2556
GO:0099095	ligand-gated anion channel activity	6/544	1.62E-05	0.000528109	2558/2554/2562/2743/2567/2556
GO:0022851	GABA-gated chloride ion channel activity	5/544	1.82E-05	0.000567743	2558/2554/2562/2567/2556
GO:0016775	phosphotransferase activity, nitrogenous group as acceptor	5/544	2.88E-05	0.000861724	27133/23415/548596/1159/3756
GO:1904315	transmitter-gated ion channel activity involved in regulation of postsynaptic membrane potential	8/544	3.65E-05	0.001049167	2561/2558/2554/2562/2743/2567/2898/2556
GO:0015276	ligand-gated ion channel activity	15/544	3.95E-05	0.001052599	2891/2561/3765/2558/2554/2562/2743/2567/2898/6262/2899/3760/3762/57338/2556
GO:0022834	ligand-gated channel activity	15/544	3.95E-05	0.001052599	2891/2561/3765/2558/2554/2562/2743/2567/2898/6262/2899/3760/3762/57338/2556
GO:0016917	GABA receptor activity	6/544	4.11E-05	0.001054483	2561/2558/2554/2562/2567/2556
GO:0005251	delayed rectifier potassium channel activity	7/544	5.44E-05	0.001349497	3746/9312/3739/3738/3747/23630/3756
GO:0099529	neurotransmitter receptor activity involved in regulation of postsynaptic membrane potential	8/544	6.14E-05	0.001472077	2561/2558/2554/2562/2743/2567/2898/2556
GO:0005230	extracellular ligand-gated ion channel activity	10/544	9.84E-05	0.002283192	2891/2561/2558/2554/2562/2743/2567/2898/2899/2556
GO:0008503	benzodiazepine receptor activity	4/544	0.000248801	0.005590256	2558/2554/2567/2556
GO:0015108	chloride transmembrane transporter activity	11/544	0.000258284	0.005627451	2561/2558/2554/2562/2743/84561/2567/10861/6505/266675/2556
GO:0005516	calmodulin binding	16/544	0.000312139	0.006600831	6546/4641/815/6857/27133/5137/8536/6262/5533/4625/5260/816/6543/5530/3756/10777
GO:0015103	inorganic anion transmembrane transporter activity	14/544	0.000368256	0.007565023	2561/2558/2554/2562/2743/84561/2567/8671/10861/54020/6505/266675/6508/2556
GO:0009975	cyclase activity	5/544	0.000467503	0.009035669	108/2983/4882/111/2977
GO:0016849	phosphorus-oxygen lyase activity	5/544	0.000467503	0.009035669	108/2983/4882/111/2977
GO:0008017	microtubule binding	18/544	0.000488957	0.009035669	84557/1780/23046/3834/57576/341019/283726/10841/90990/55638/25876/26153/89795/10634/24139/85462/643246/152789
GO:0044325	ion channel binding	12/544	0.000490113	0.009035669	6546/7881/2257/27133/3746/88/6262/3747/23630/389816/3756/388591
GO:0005544	calcium-dependent phospholipid binding	7/544	0.001153485	0.020733899	6857/57699/131034/27445/6861/148281/311
GO:0045499	chemorepellent activity	5/544	0.001261965	0.022130555	57715/80031/9037/10371/23768
GO:0008509	anion transmembrane transporter activity	21/544	0.0014659	0.025094813	2561/8604/2558/2554/2562/9194/2743/140679/84561/2567/53919/8671/10861/23428/54020/6505/6529/266675/6508/2556/6534
GO:0015291	secondary active transmembrane transporter activity	16/544	0.00156805	0.026219262	6546/9194/140679/84561/53919/8671/50651/10861/54020/6505/6529/388662/6543/6508/6553/6534
GO:0005254	chloride channel activity	8/544	0.00214401	0.035035065	2561/2558/2554/2562/2743/2567/266675/2556
GO:0030506	ankyrin binding	4/544	0.002929312	0.045595526	6546/57731/776/6710
GO:0008331	high voltage-gated calcium channel activity	3/544	0.002980514	0.045595526	783/776/773
GO:0015467	G-protein activated inward rectifier potassium channel activity	3/544	0.002980514	0.045595526	3765/3760/3762

Go_Down_Day60

ID	Description	GeneRatio	pvalue	p.adjust	geneID
GO:0048880	sensory system development	33/401	2.45E-12	8.18E-09	7020/7477/5457/4081/64093/7021/7431/10763/5176/793/7068/54549/58158/658/4760/8929/23554/2195/4124/1969/5458/182/64220/341640/2820/2201/4088/7474/113246/5764/10265/7048/55636
GO:0072001	renal system development	29/401	5.68E-12	8.18E-09	56603/7020/7477/7021/5176/793/51301/26508/10736/8837/4038/59/7161/1592/760/4091/182/64220/79633/5314/4088/7474/4868/55366/4643/285/4854/79191/4851
GO:0007389	pattern specification process	36/401	5.76E-12	8.18E-09	56603/7477/653/23242/2637/4488/4010/340260/4487/54626/8481/4281/7545/441478/1959/10736/390992/4038/3955/658/4760/5081/7479/4091/286204/4036/50805/4088/7474/6423/7048/5992/27023/23493/79191/4851
GO:0001822	kidney development	28/401	8.47E-12	9.02E-09	56603/7020/7477/7021/5176/793/51301/26508/10736/8837/4038/59/7161/1592/760/4091/182/64220/79633/5314/4088/4868/55366/4643/285/4854/79191/4851
GO:0001503	ossification	33/401	1.34E-11	1.14E-08	7020/7477/653/64093/4488/2662/196527/5251/1959/10736/8519/4038/2274/8817/658/4880/3636/3397/1969/4091/182/2261/5167/79633/2201/340419/4088/7474/55366/5745/5764/6423/4851
GO:0001655	urogenital system development	30/401	2.07E-11	1.25E-08	56603/7020/7477/7021/5176/793/51301/26508/10736/8837/4038/59/7161/1592/760/4091/182/64220/79633/5314/4088/7474/4868/55366/4643/285/4854/27023/79191/4851
GO:0043010	camera-type eye development	29/401	2.17E-11	1.25E-08	7020/7477/4081/7021/7431/10763/5176/793/7068/54549/58158/658/4760/23554/2195/4124/1969/5458/182/64220/2820/2201/4088/7474/113246/5764/10265/7048/55636
GO:0002009	morphogenesis of an epithelium	37/401	2.34E-11	1.25E-08	7020/7477/653/23242/2637/57167/4488/4919/7424/6788/7052/441478/389/10736/84133/4478/1969/2239/760/182/8914/341640/79633/407006/4036/340419/780/4088/7474/55366/11331/6423/151449/7048/3678/79191/4851
GO:0001654	eye development	31/401	3.74E-11	1.71E-08	7020/7477/4081/64093/7021/7431/10763/5176/793/7068/54549/58158/658/4760/23554/2195/4124/1969/5458/182/64220/341640/2820/2201/4088/7474/113246/5764/10265/7048/55636
GO:0150063	visual system development	31/401	4.02E-11	1.71E-08	7020/7477/4081/64093/7021/7431/10763/5176/793/7068/54549/58158/658/4760/23554/2195/4124/1969/5458/182/64220/341640/2820/2201/4088/7474/113246/5764/10265/7048/55636
GO:0071772	response to BMP	21/401	7.47E-11	2.65E-08	653/7021/4488/4487/26508/4038/658/8929/64102/3397/348093/4091/8239/286204/4036/7474/390598/6423/285704/151449/4851
GO:0071773	cellular response to BMP stimulus	21/401	7.47E-11	2.65E-08	653/7021/4488/4487/26508/4038/658/8929/64102/3397/348093/4091/8239/286204/4036/7474/390598/6423/285704/151449/4851
GO:0003002	regionalization	29/401	4.00E-10	1.31E-07	56603/7477/23242/2637/4488/4010/4487/54626/441478/1959/10736/390992/4038/3955/658/4760/5081/7479/4091/286204/4036/4088/7474/6423/5992/27023/23493/79191/4851
GO:0061448	connective tissue development	25/401	6.72E-10	2.04E-07	7477/653/64641/4488/340260/4487/10736/8837/59/8817/658/4880/3696/64102/5081/5458/2261/340419/4088/7474/5745/6423/151449/7048/4851
GO:0030278	regulation of ossification	21/401	1.41E-09	3.99E-07	7020/7477/64093/4488/2662/196527/1959/10736/8519/4038/658/4880/3397/4091/182/5167/2201/4088/7474/6423/4851
GO:0030509	BMP signaling pathway	18/401	5.55E-09	1.48E-06	653/7021/4488/4487/4038/658/3397/348093/4091/8239/286204/4036/7474/390598/6423/285704/151449/4851
GO:0007178	transmembrane receptor protein serine/threonine kinase signaling pathway	27/401	7.20E-09	1.80E-06	653/7021/4488/7431/2662/4487/4038/658/3654/2353/3397/348093/4091/8239/7057/286204/4036/2201/4088/7474/857/390598/6423/285704/151449/7048/4851
GO:0021953	central nervous system neuron differentiation	19/401	8.94E-09	2.11E-06	5457/4009/2637/6900/869/167826/340260/3251/266727/8929/219409/5081/6095/7474/401/113246/6423/151449/1630
GO:0048593	camera-type eye morphogenesis	15/401	1.19E-08	2.66E-06	7020/7021/793/7068/54549/23554/2195/4124/1969/182/64220/7474/113246/5764/10265
GO:0007517	muscle organ development	29/401	1.80E-08	3.84E-06	56603/5457/5077/4487/84466/7004/1959/26508/11149/6938/8837/7161/2353/8929/5081/64220/3344/4036/4088/7474/857/4868/64208/7048/55636/8531/6445/23493/4851
GO:0048565	digestive tract development	16/401	2.53E-08	5.13E-06	23242/10736/100/57103/348093/64220/79633/5919/1373/4088/7474/55366/6423/7048/3815/4851
GO:0090596	sensory organ morphogenesis	22/401	3.18E-08	6.16E-06	56603/7020/2637/7021/793/4487/7068/54549/7545/10736/23554/2195/4124/1969/182/64220/2201/7474/113246/5764/10265/55636
GO:1902041	regulation of extrinsic apoptotic signaling pathway via death domain receptors	11/401	3.96E-08	7.34E-06	653/3958/6788/8837/658/7057/407006/5366/4118/6423/3162
GO:0072006	nephron development	16/401	4.33E-08	7.67E-06	7477/7021/793/26508/10736/8837/59/182/79633/4868/55366/4643/285/4854/79191/4851
GO:0045165	cell fate commitment	22/401	4.86E-08	8.28E-06	56603/7477/5457/64641/30012/167826/5454/7005/10736/58158/4760/219409/5081/7479/182/6095/7474/4854/6423/151449/23493/4851
GO:0021510	spinal cord development	14/401	5.48E-08	8.94E-06	167826/340260/7545/26227/266727/219409/5081/401/5764/151449/5992/27023/1630/4851
GO:0003407	neural retina development	11/401	5.74E-08	8.94E-06	7020/7021/793/7068/54549/58158/4760/23554/5458/5764/10265
GO:0090287	regulation of cellular response to growth factor stimulus	23/401	5.88E-08	8.94E-06	7021/4488/143282/8862/8573/4487/7424/8837/8817/348093/4091/7057/286204/4036/2201/4088/7474/857/390598/6423/7048/3678/4851
GO:0055123	digestive system development	16/401	8.81E-08	1.28E-05	23242/10736/100/57103/348093/64220/79633/5919/1373/4088/7474/55366/6423/7048/3815/4851
GO:0048732	gland development	29/401	9.01E-08	1.28E-05	7477/23242/4488/5176/8862/4487/9076/7068/5454/7052/100/8837/4478/219409/4124/1969/64220/1373/780/4088/7474/857/11331/5764/151449/7048/27023/3162/4851
GO:0001666	response to hypoxia	24/401	1.03E-07	1.42E-05	79625/10631/7852/5024/7424/3708/100/8837/3654/57103/4880/28984/7057/6095/4088/5366/857/285/81575/5764/7200/7048/3162/4851
GO:0048592	eye morphogenesis	16/401	1.07E-07	1.43E-05	7020/7021/793/7068/54549/23554/2195/4124/1969/182/64220/2201/7474/113246/5764/10265
GO:0035107	appendage morphogenesis	16/401	1.18E-07	1.48E-05	56603/7020/57167/7021/4488/4487/4038/658/341640/2201/340419/7474/344191/6423/55636/4851
GO:0035108	limb morphogenesis	16/401	1.18E-07	1.48E-05	56603/7020/57167/7021/4488/4487/4038/658/341640/2201/340419/7474/344191/6423/55636/4851
GO:0043583	ear development	19/401	1.70E-07	2.01E-05	7020/653/2103/2637/4919/4487/7545/10736/4760/8929/182/64220/341640/79633/780/7474/55636/23493/4851
GO:0048754	branching morphogenesis of an epithelial tube	16/401	1.72E-07	2.01E-05	2637/4488/441478/10736/1969/8914/79633/340419/780/7474/55366/11331/6423/151449/7048/4851
GO:0048568	embryonic organ development	28/401	1.75E-07	2.01E-05	7020/7477/653/23242/2637/10763/4487/7545/6788/2020/10736/100/4760/348093/1969/64220/286204/5919/2201/4088/7474/10265/3856/7048/3815/55636/23493/4851
GO:0070482	response to oxygen levels	25/401	1.94E-07	2.13E-05	79625/10631/7852/5024/7424/3708/100/8837/3654/57103/4880/28984/5458/7057/6095/4088/5366/857/285/81575/5764/7200/7048/3162/4851
GO:0036293	response to decreased oxygen levels	24/401	1.95E-07	2.13E-05	79625/10631/7852/5024/7424/3708/100/8837/3654/57103/4880/28984/7057/6095/4088/5366/857/285/81575/5764/7200/7048/3162/4851
GO:0001649	osteoblast differentiation	19/401	2.28E-07	2.43E-05	7477/64093/4488/2662/8519/2274/658/4880/3397/1969/4091/182/2201/340419/4088/55366/5745/6423/4851
GO:0060541	respiratory system development	18/401	2.47E-07	2.56E-05	7477/7068/5251/100/3988/8817/8929/3397/4124/64220/8914/340419/7474/9509/5764/7048/55636/4851
GO:0045778	positive regulation of ossification	12/401	2.54E-07	2.57E-05	7020/7477/4488/196527/8519/658/4880/182/2201/4088/7474/6423
GO:0048736	appendage development	17/401	2.77E-07	2.68E-05	56603/7020/57167/64093/7021/4488/4487/4038/658/341640/2201/340419/7474/344191/6423/55636/4851
GO:0060173	limb development	17/401	2.77E-07	2.68E-05	56603/7020/57167/64093/7021/4488/4487/4038/658/341640/2201/340419/7474/344191/6423/55636/4851
GO:0035282	segmentation	13/401	3.15E-07	2.98E-05	23242/54626/441478/1959/390992/3955/286204/4088/7474/6423/27023/79191/4851
GO:0045667	regulation of osteoblast differentiation	14/401	3.74E-07	3.46E-05	7477/64093/4488/2662/8519/658/4880/3397/4091/182/2201/4088/6423/4851

GO:0060562	epithelial tube morphogenesis	23/401	3.84E-07	3.48E-05	653/23242/2637/57167/4488/6788/441478/10736/1969/8914/79633/4036/340419/780/4088/7474/55366/11331/6423/151449/7048/79191/4851
GO:0001756	somitogenesis	11/401	4.01E-07	3.56E-05	23242/54626/441478/390992/3955/286204/4088/7474/6423/27023/4851
GO:0060041	retina development in camera-type eye	15/401	4.33E-07	3.76E-05	7020/7021/5176/793/7068/54549/58158/658/4760/23554/4124/5458/5764/10265/55636
GO:0061138	morphogenesis of a branching epithelium	17/401	4.84E-07	4.12E-05	2637/4488/7052/441478/10736/1969/8914/79633/340419/780/7474/55366/11331/6423/151449/7048/4851
GO:0060537	muscle tissue development	26/401	5.65E-07	4.72E-05	56603/5457/653/84466/1959/26508/11149/8837/7161/2274/2353/8929/5081/64220/3344/4036/4088/7474/857/4868/64208/7048/55636/8531/23493/4851
GO:0032835	glomerulus development	10/401	5.89E-07	4.82E-05	26508/8837/59/182/4868/55366/4643/285/4854/4851
GO:0051216	cartilage development	18/401	6.02E-07	4.83E-05	7477/653/4488/340260/4487/10736/8817/658/4880/3696/5081/2261/340419/4088/7474/5745/6423/7048
GO:0060042	retina morphogenesis in camera-type eye	9/401	6.37E-07	5.02E-05	7020/7021/793/7068/54549/23554/4124/5764/10265
GO:0003184	pulmonary valve morphogenesis	6/401	6.67E-07	5.07E-05	26508/4091/182/64220/23493/4851
GO:0003279	cardiac septum development	13/401	6.86E-07	5.07E-05	653/57167/4488/26508/4091/182/64220/4036/7474/7048/55636/23493/4851
GO:0060348	bone development	18/401	6.94E-07	5.07E-05	56603/7020/4488/4487/196527/5251/8817/658/4880/3636/80263/5358/2261/79633/158326/6423/7048/3815
GO:2000027	regulation of animal organ morphogenesis	18/401	6.94E-07	5.07E-05	7020/4919/4487/7068/2787/10736/8837/84133/2239/182/340419/7474/55366/11331/5764/6423/7048/4851
GO:0060411	cardiac septum morphogenesis	11/401	7.03E-07	5.07E-05	653/4488/26508/4091/182/4036/7474/7048/55636/23493/4851
GO:0003205	cardiac chamber development	16/401	7.48E-07	5.31E-05	5457/653/57167/4488/26508/2274/4091/182/64220/4036/7474/6423/7048/55636/23493/4851
GO:0048762	mesenchymal cell differentiation	18/401	7.98E-07	5.57E-05	653/2637/4488/4487/223117/26508/10736/8929/28984/182/286204/407006/4088/7474/6423/7048/23493/4851
GO:0003206	cardiac chamber morphogenesis	14/401	8.23E-07	5.65E-05	5457/653/4488/26508/2274/4091/182/4036/7474/6423/7048/55636/23493/4851
GO:0030324	lung development	16/401	8.78E-07	5.93E-05	7477/7068/5251/100/3988/8817/3397/4124/64220/8914/340419/7474/9509/5764/7048/4851
GO:0014706	striated muscle tissue development	25/401	9.00E-07	5.99E-05	56603/5457/653/84466/1959/26508/11149/8837/7161/2274/2353/8929/5081/3344/4036/4088/7474/857/4868/64208/7048/55636/8531/23493/4851
GO:0072132	mesenchyme morphogenesis	9/401	9.24E-07	6.05E-05	653/4488/4487/26508/59/4088/7474/7048/4851
GO:0060538	skeletal muscle organ development	16/401	9.50E-07	6.12E-05	56603/84466/1959/26508/11149/8837/2353/8929/5081/64220/3344/857/4868/64208/8531/4851
GO:0090092	regulation of transmembrane receptor protein serine/threonine kinase signaling pathway	19/401	9.62E-07	6.12E-05	653/7021/4488/2662/4487/348093/4091/7057/286204/4036/2201/4088/7474/857/390598/6423/151449/7048/4851
GO:0072089	stem cell proliferation	13/401	1.15E-06	7.22E-05	7477/54894/10763/7424/7005/10736/84133/58158/2120/407006/7474/6423/4851
GO:0030323	respiratory tube development	16/401	1.20E-06	7.41E-05	7477/7068/5251/100/3988/8817/3397/4124/64220/8914/340419/7474/9509/5764/7048/4851
GO:0061564	axon development	29/401	1.27E-06	7.70E-05	64221/5457/23242/8633/4009/2637/7852/6900/223117/5454/1959/3996/4038/658/91653/8929/5458/2335/8239/407006/780/2044/7474/1945/113246/151449/27023/1630/4851
GO:0045665	negative regulation of neuron differentiation	18/401	1.29E-06	7.71E-05	4009/6900/7431/30012/223117/7068/9745/4038/7161/8929/3397/5458/182/7474/4854/79191/1630/4851
GO:0001763	morphogenesis of a branching structure	17/401	1.37E-06	8.10E-05	2637/4488/7052/441478/10736/1969/8914/79633/340419/780/7474/55366/11331/6423/151449/7048/4851
GO:0001837	epithelial to mesenchymal transition	14/401	1.43E-06	8.34E-05	653/4488/4487/26508/28984/182/286204/407006/4088/7474/6423/7048/23493/4851
GO:0008625	extrinsic apoptotic signaling pathway via death domain receptors	11/401	1.95E-06	0.000111194	653/3958/6788/8837/658/7057/407006/5366/4118/6423/3162
GO:0007409	axonogenesis	27/401	1.96E-06	0.000111194	64221/5457/23242/8633/4009/2637/7852/6900/223117/5454/1959/3996/4038/658/91653/8929/5458/2335/8239/2044/7474/1945/113246/151449/27023/1630/4851
GO:0022612	gland morphogenesis	13/401	2.07E-06	0.000116189	4488/7052/8837/4478/1969/780/7474/857/11331/5764/151449/7048/4851
GO:0003177	pulmonary valve development	6/401	2.14E-06	0.000118281	26508/4091/182/64220/23493/4851
GO:0090288	negative regulation of cellular response to growth factor stimulus	15/401	2.18E-06	0.000119027	8862/8573/8837/348093/4091/7057/4036/2201/4088/7474/857/390598/6423/7048/4851
GO:0072073	kidney epithelium development	14/401	2.21E-06	0.000119286	7477/7021/793/26508/10736/4091/182/79633/4088/4868/55366/4643/79191/4851
GO:0007519	skeletal muscle tissue development	15/401	2.36E-06	0.000123934	56603/84466/1959/26508/11149/8837/2353/8929/5081/3344/857/4868/64208/8531/4851
GO:0021915	neural tube development	15/401	2.36E-06	0.000123934	653/23242/2637/57167/6788/390992/26227/5081/1969/4036/7474/6423/151449/27023/4851
GO:0030900	forebrain development	24/401	2.41E-06	0.000125315	7477/5457/4009/2637/6900/340260/4487/5454/3251/4760/5453/219409/79633/4036/2044/7474/4854/113246/5764/151449/5992/55636/27023/4851
GO:0002011	morphogenesis of an epithelial sheet	9/401	2.55E-06	0.000130817	653/4488/389/182/407006/780/7474/3678/4851
GO:0030282	bone mineralization	12/401	2.63E-06	0.000133198	7020/196527/5251/658/2261/5167/2201/340419/4088/55366/5745/5764
GO:0030510	regulation of BMP signaling pathway	11/401	3.11E-06	0.000155957	7021/4488/4487/348093/4091/286204/4036/7474/390598/6423/4851
GO:0030326	embryonic limb morphogenesis	13/401	3.29E-06	0.000160924	56603/7020/57167/4488/4487/4038/341640/2201/340419/7474/6423/55636/4851
GO:0035113	embryonic appendage morphogenesis	13/401	3.29E-06	0.000160924	56603/7020/57167/4488/4487/4038/341640/2201/340419/7474/6423/55636/4851
GO:0003231	cardiac ventricle development	13/401	3.60E-06	0.000173965	5457/57167/26508/4091/182/64220/4036/7474/6423/7048/55636/23493/4851
GO:0009952	anterior/posterior pattern specification	17/401	3.76E-06	0.00017999	23242/2637/4488/4487/54626/441478/10736/390992/3955/4760/286204/4088/7474/6423/27023/23493/4851
GO:0007219	Notch signaling pathway	16/401	3.84E-06	0.000181571	10631/54626/23286/441478/8788/26508/390992/3955/58158/182/79633/4854/7048/3815/23493/4851
GO:0030902	hindbrain development	14/401	3.95E-06	0.000184751	5457/653/4009/2637/869/57626/2020/1959/390992/4760/8929/6095/401/5764
GO:0072009	nephron epithelium development	12/401	4.32E-06	0.000198867	7477/7021/793/26508/10736/182/79633/4868/55366/4643/79191/4851
GO:0061053	somite development	11/401	4.34E-06	0.000198867	23242/54626/441478/390992/3955/286204/4088/7474/6423/27023/4851
GO:0060317	cardiac epithelial to mesenchymal transition	7/401	4.51E-06	0.00020418	4488/4487/26508/182/7048/23493/4851
GO:0031214	biomineral tissue development	14/401	5.01E-06	0.000224356	7020/4488/196527/5251/658/2261/5167/2201/340419/4088/55366/5745/5764/4851
GO:0060349	bone morphogenesis	12/401	5.23E-06	0.000232031	56603/7020/4488/4487/8817/658/4880/3636/2261/158326/6423/7048
GO:0042063	gliogenesis	19/401	6.00E-06	0.000263421	7852/928/147495/6900/7431/4919/5454/1959/26227/7161/58158/5453/8929/85414/407006/4036/2934/5764/4851
GO:0003151	outflow tract morphogenesis	10/401	6.95E-06	0.000302011	4488/26508/4091/182/4036/7474/6423/7048/23493/4851
GO:0060485	mesenchyme development	19/401	7.05E-06	0.000303273	653/2637/4488/4487/223117/26508/10736/59/8929/28984/182/286204/407006/4088/7474/6423/7048/23493/4851
GO:0003272	endocardial cushion formation	6/401	7.39E-06	0.000314827	653/4488/4487/26508/7048/4851
GO:0071542	dopaminergic neuron differentiation	7/401	8.51E-06	0.000358947	4009/4010/8929/340419/7474/401/6423
GO:0051961	negative regulation of nervous system development	20/401	8.69E-06	0.000362764	4009/6900/7431/30012/223117/7068/9745/4038/7161/8929/3397/5458/182/7474/4854/5764/5992/79191/1630/4851
GO:0002064	epithelial cell development	16/401	8.99E-06	0.000371798	7477/653/7431/9076/59/4478/57103/64102/2195/3397/1969/182/7474/4868/4643/4851
GO:0010721	negative regulation of cell development	21/401	9.74E-06	0.000398612	4009/10631/6900/7431/30012/223117/7068/9745/4038/7161/8929/4880/3397/5458/182/7474/4854/5764/79191/1630/4851
GO:0003007	heart morphogenesis	18/401	1.11E-05	0.000450763	5457/653/4488/4487/26508/2787/2274/4091/182/79633/4036/4088/7474/6423/7048/55636/23493/4851
GO:0050768	negative regulation of neurogenesis	19/401	1.19E-05	0.000476287	4009/6900/7431/30012/223117/7068/9745/4038/7161/8929/3397/5458/182/7474/4854/5764/79191/1630/4851
GO:0007631	feeding behavior	11/401	1.20E-05	0.000478341	5457/127343/8862/79924/10874/4986/2353/1602/64220/9607/7200
GO:0050890	cognition	19/401	1.60E-05	0.00063051	4009/6900/5176/793/54207/25978/1959/4986/2353/64220/4908/10882/57477/113246/5764/3815/3678/55636/27023
GO:0001701	in utero embryonic development	20/401	1.62E-05	0.000633974	7477/653/57167/4010/4487/4952/6788/100/390992/4124/5919/5932/4088/4643/3856/7048/55636/8531/23493/4851
GO:0048705	skeletal system morphogenesis	17/401	1.66E-05	0.000641394	56603/7020/4488/340260/4487/10736/8817/658/4880/3636/2261/158326/2201/4088/10265/6423/7048

GO:0003198	epithelial to mesenchymal transition involved in endocardial cushion formation	5/401	1.68E-05	0.000643252	4488/4487/26508/7048/4851
GO:1901342	regulation of vasculature development	24/401	1.77E-05	0.000673996	5176/79924/7424/8837/8817/23554/3696/28984/64102/3397/1969/7057/407006/7474/285/5764/6423/7048/3815/3678/5168/3162/23493/4851
GO:0061005	cell differentiation involved in kidney development	8/401	1.98E-05	0.000746809	10736/59/182/79633/4868/55366/4643/4851
GO:0035914	skeletal muscle cell differentiation	9/401	2.20E-05	0.000821818	56603/84466/1959/26508/2353/8929/5081/3344/4851
GO:0010001	glial cell differentiation	15/401	2.27E-05	0.000836472	7852/928/6900/7431/4919/5454/1959/26227/7161/58158/5453/8929/85414/2934/4851
GO:1904019	epithelial cell apoptotic process	11/401	2.28E-05	0.000836472	79625/6788/196527/8837/4760/28984/7057/2934/3856/7048/3162
GO:1902043	positive regulation of extrinsic apoptotic signaling pathway via death domain receptors	5/401	2.33E-05	0.000849192	6788/658/7057/5366/4118
GO:0003208	cardiac ventricle morphogenesis	9/401	2.47E-05	0.000882763	5457/26508/182/4036/6423/7048/55636/23493/4851
GO:0003281	ventricular septum development	9/401	2.47E-05	0.000882763	57167/26508/4091/64220/4036/7474/7048/23493/4851
GO:0030901	midbrain development	10/401	3.04E-05	0.001079864	4009/4487/2020/390992/445582/7474/401/6423/151449/27023
GO:0061311	cell surface receptor signaling pathway involved in heart development	6/401	3.12E-05	0.001096672	4488/4487/182/7474/23493/4851
GO:0097191	extrinsic apoptotic signaling pathway	16/401	3.24E-05	0.00113217	653/3958/6788/8837/658/2261/7057/407006/4088/5366/857/4118/6423/3856/3162/1630
GO:0072080	nephron tubule development	10/401	3.35E-05	0.001158007	7477/7021/793/26508/10736/182/79633/55366/79191/4851
GO:0003197	endocardial cushion development	7/401	3.51E-05	0.001206719	653/4488/4487/26508/182/7048/4851
GO:0061326	renal tubule development	10/401	4.03E-05	0.001371468	7477/7021/793/26508/10736/182/79633/55366/79191/4851
GO:0035850	epithelial cell differentiation involved in kidney development	7/401	4.10E-05	0.00138616	10736/59/182/79633/4868/4643/4851
GO:0048486	parasympathetic nervous system development	5/401	4.23E-05	0.001418386	7020/1959/390992/8929/401
GO:0048545	response to steroid hormone	22/401	4.63E-05	0.001541683	7477/2103/5176/9076/7068/26508/8837/81285/2274/2353/5458/760/7057/6095/1373/857/11331/5764/7200/7048/5931/4851
GO:0048483	autonomic nervous system development	7/401	4.77E-05	0.001561766	7020/2637/7021/1959/390992/8929/401
GO:0048839	inner ear development	14/401	4.77E-05	0.001561766	7020/2103/2637/4919/7545/4760/8929/182/341640/79633/7474/55636/23493/4851
GO:0045669	positive regulation of osteoblast differentiation	8/401	4.91E-05	0.001597558	7477/4488/8519/658/4880/182/2201/6423
GO:0060443	mammary gland morphogenesis	7/401	5.52E-05	0.001773851	4488/1969/780/7474/857/11331/7048
GO:0032495	response to muramyl dipeptide	5/401	5.54E-05	0.001773851	7431/64170/55765/182/4851
GO:1905314	semi-lunar valve development	6/401	5.63E-05	0.001788102	26508/4091/182/64220/23493/4851
GO:0050920	regulation of chemotaxis	15/401	5.70E-05	0.001791196	7852/7114/7424/223117/196527/8817/5458/7057/158747/5919/4908/4088/7474/285/4851
GO:0050769	positive regulation of neurogenesis	24/401	5.72E-05	0.001791196	653/23242/79625/7852/5176/7424/26508/6938/8837/127833/7161/4760/8929/4124/5458/2335/407006/4036/7474/5764/151449/3815/79191/4851
GO:2000241	regulation of reproductive process	12/401	6.32E-05	0.001964737	4488/5176/4487/10184/6788/100/3955/4880/7474/55726/10549/4851
GO:0048608	reproductive structure development	23/401	6.58E-05	0.002029449	7477/653/5176/6788/100/3955/658/3696/64220/4036/7474/55366/10265/6423/3856/151449/3815/55636/8531/10549/6194/23493/4851
GO:0003203	endocardial cushion morphogenesis	6/401	6.75E-05	0.002068621	653/4488/4487/26508/7048/4851
GO:0061458	reproductive system development	23/401	7.31E-05	0.002193607	7477/653/5176/6788/100/3955/658/3696/64220/4036/7474/55366/10265/6423/3856/151449/3815/55636/8531/10549/6194/23493/4851
GO:0003179	heart valve morphogenesis	7/401	7.32E-05	0.002193607	26508/4091/182/64220/7048/23493/4851
GO:0061383	trabecula morphogenesis	7/401	7.32E-05	0.002193607	653/4488/2274/2201/55636/23493/4851
GO:0090101	negative regulation of transmembrane receptor protein serine/threonine kinase signaling pathway	11/401	7.62E-05	0.002269004	348093/4091/4036/2201/4088/7474/857/390598/6423/7048/4851
GO:0016055	Wnt signaling pathway	24/401	8.03E-05	0.002375287	7477/54894/147495/4919/6788/441478/4343/4038/84133/129293/284654/23554/7479/2239/83439/388630/340419/4088/7474/27303/857/55366/6423/4851
GO:0071560	cellular response to transforming growth factor beta stimulus	16/401	8.18E-05	0.002394006	10631/2662/9076/8837/658/2353/3397/4091/8239/7057/2201/4088/7474/857/390598/7048
GO:0045444	fat cell differentiation	15/401	8.26E-05	0.002394006	64641/7021/4488/2662/6788/1959/4091/182/5167/252995/6095/5919/4088/7474/6423
GO:0010718	positive regulation of epithelial to mesenchymal transition	7/401	8.38E-05	0.002394006	28984/182/286204/407006/4088/7048/4851
GO:0021545	cranial nerve development	7/401	8.38E-05	0.002394006	7020/5457/1959/390992/8929/401/55636
GO:0048546	digestive tract morphogenesis	7/401	8.38E-05	0.002394006	10736/348093/64220/4088/7474/6423/4851
GO:0198738	cell-cell signaling by wnt	24/401	8.58E-05	0.002436954	7477/54894/147495/4919/6788/441478/4343/4038/84133/129293/284654/23554/7479/2239/83439/388630/340419/4088/7474/27303/857/55366/6423/4851
GO:0003181	atrioventricular valve morphogenesis	5/401	9.08E-05	0.00251071	26508/4091/7048/23493/4851
GO:0010842	retina layer formation	5/401	9.08E-05	0.00251071	7020/7021/793/54549/23554
GO:0021516	dorsal spinal cord development	5/401	9.08E-05	0.00251071	340260/266727/219409/5081/151449
GO:0072012	glomerulus vasculature development	5/401	9.08E-05	0.00251071	8837/59/285/4854/4851
GO:0014831	gastro-intestinal system smooth muscle contraction	4/401	9.39E-05	0.002547001	10874/5733/80763/3815
GO:0072017	distal tubule development	4/401	9.39E-05	0.002547001	7021/793/182/4851
GO:0097284	hepatocyte apoptotic process	4/401	9.39E-05	0.002547001	6788/8837/2934/3856
GO:0045765	regulation of angiogenesis	21/401	9.54E-05	0.002570871	5176/79924/7424/8817/23554/3696/28984/64102/3397/1969/7057/407006/7474/285/5764/6423/7048/3678/5168/3162/4851
GO:0048663	neuron fate commitment	8/401	9.72E-05	0.002603515	5457/30012/167826/5454/219409/5081/4854/4851
GO:0016331	morphogenesis of embryonic epithelium	12/401	0.000100623	0.002677823	7020/7477/653/23242/57167/7424/6788/4036/7474/6423/151449/79191
GO:0051090	regulation of DNA-binding transcription factor activity	22/401	0.000103611	0.002740209	5457/4488/7114/4919/6788/26508/7546/8837/3654/4760/2353/28984/3397/5458/4796/5314/4088/2044/7474/11331/3815/3162
GO:0030514	negative regulation of BMP signaling pathway	7/401	0.000108785	0.002841748	348093/4091/4036/7474/390598/6423/4851
GO:2000677	regulation of transcription regulatory region DNA binding	7/401	0.000108785	0.002841748	5457/4488/7114/4487/4760/5458/23493
GO:0048640	negative regulation of developmental growth	10/401	0.000111224	0.002887764	80014/223117/23286/6788/7161/2261/7474/6423/7048/1630
GO:0035137	hindlimb morphogenesis	6/401	0.000112415	0.002900999	7021/4488/4487/340419/55636/4851
GO:0071559	response to transforming growth factor beta	16/401	0.000114172	0.002928579	10631/2662/9076/8837/658/2353/3397/4091/8239/7057/2201/4088/7474/857/390598/7048
GO:0010717	regulation of epithelial to mesenchymal transition	9/401	0.000122126	0.003113852	653/28984/182/286204/407006/4088/6423/7048/4851
GO:0021515	cell differentiation in spinal cord	7/401	0.000123391	0.003127373	167826/266727/219409/5081/401/151449/4851
GO:0072044	collecting duct development	4/401	0.000133334	0.003359387	7477/7021/793/4851
GO:0048638	regulation of developmental growth	19/401	0.00013577	0.003399434	80014/223117/23286/5454/6788/127833/7161/4880/5458/2335/2261/7474/6423/7048/55636/8531/23493/1630/4851
GO:0050673	epithelial cell proliferation	22/401	0.00013652	0.003399434	653/5176/8862/8573/7424/441478/8837/28984/64102/3397/1969/7057/4088/7474/857/55366/11331/5764/6423/3815/3162/4851
GO:0003171	atrioventricular valve development	5/401	0.000141422	0.003460768	26508/4091/7048/23493/4851
GO:0061437	renal system vasculature development	5/401	0.000141422	0.003460768	8837/59/285/4854/4851
GO:0061440	kidney vasculature development	5/401	0.000141422	0.003460768	8837/59/285/4854/4851
GO:0061180	mammary gland epithelium development	8/401	0.000147079	0.003578632	7477/4488/4487/1969/780/7474/11331/27023
GO:0045446	endothelial cell differentiation	10/401	0.000151119	0.003656058	7477/9076/4478/64102/3397/182/81575/5764/23493/4851
GO:0035329	hippo signaling	6/401	0.000153612	0.003695359	80014/23286/7005/6788/7004/79633
GO:2001236	regulation of extrinsic apoptotic signaling pathway	12/401	0.000155663	0.00372368	653/3958/6788/8837/658/7057/407006/5366/857/4118/6423/3162
GO:0003170	heart valve development	7/401	0.000157382	0.003743755	26508/4091/182/64220/7048/23493/4851
GO:0048844	artery morphogenesis	8/401	0.00016238	0.003841179	7021/182/64220/4036/4854/55636/23493/4851
GO:0030111	regulation of Wnt signaling pathway	18/401	0.000163701	0.003851033	54894/147495/6788/441478/4038/84133/129293/284654/83439/388630/340419/4088/7474/27303/857/55366/6423/4851

GO:0048562	embryonic organ morphogenesis	17/401	0.000166063	0.003878295	7020/2637/4487/7545/10736/4760/348093/1969/64220/286204/2201/4088/7474/10265/7048/55636/4851
GO:0007369	gastrulation	13/401	0.000166681	0.003878295	8862/10736/7479/1969/2335/286204/407006/4088/7474/6423/7048/3678/6194
GO:0060444	branching involved in mammary gland duct morphogenesis	5/401	0.000173651	0.004018516	4488/1969/780/7474/11331
GO:0007411	axon guidance	16/401	0.000179651	0.004134876	64221/8633/4009/2637/7852/6900/223117/1959/658/91653/5458/2044/7474/1945/151449/1630
GO:0003214	cardiac left ventricle morphogenesis	4/401	0.000183481	0.004177877	6423/7048/23493/4851
GO:0021527	spinal cord association neuron differentiation	4/401	0.000183481	0.004177877	266727/219409/5081/151449
GO:0045926	negative regulation of growth	16/401	0.000187715	0.004229052	23677/80014/4487/223117/23286/6788/7161/2261/5167/4088/7474/6423/7048/5931/1630/4851
GO:0097485	neuron projection guidance	16/401	0.000187715	0.004229052	64221/8633/4009/2637/7852/6900/223117/1959/658/91653/5458/2044/7474/1945/151449/1630
GO:0035904	aorta development	7/401	0.00019858	0.004450287	7021/4091/182/4036/55636/23493/4851
GO:0008015	blood circulation	24/401	0.000201491	0.00449187	10631/143282/8862/79924/7424/7068/3708/10874/11149/100/59/81285/80763/4880/9607/1373/4088/857/5443/10265/55636/3162/6445/23493
GO:0060840	artery development	9/401	0.000202872	0.004499102	7021/4091/182/64220/4036/4854/55636/23493/4851
GO:0033002	muscle cell proliferation	15/401	0.00020895	0.004609894	8862/84466/7052/8837/7161/3654/4880/348093/7057/407006/4854/7048/3162/23493/4851
GO:0030198	extracellular matrix organization	19/401	0.000229644	0.005040336	79625/10631/8837/255631/3696/28984/2335/7057/2201/780/4088/9509/4868/4643/9507/6423/3678/10549/4851
GO:0002062	chondrocyte differentiation	10/401	0.000233165	0.005091359	4488/10736/8817/658/4880/2261/4088/5745/6423/7048
GO:0021536	diencephalon development	8/401	0.000237278	0.00515475	5457/2637/4487/5454/219409/7474/5764/27023
GO:0030178	negative regulation of Wnt signaling pathway	12/401	0.00024833	0.005367452	54894/147495/6788/4038/84133/129293/388630/7474/27303/857/6423/4851
GO:0003013	circulatory system process	24/401	0.000255604	0.005496772	10631/143282/8862/79924/7424/7068/3708/10874/11149/100/59/81285/80763/4880/9607/1373/4088/857/5443/10265/55636/3162/6445/23493
GO:0030879	mammary gland development	11/401	0.000257071	0.005500542	7477/4488/8862/4487/1969/780/7474/857/11331/7048/27023
GO:0001570	vasculogenesis	8/401	0.00025986	0.005504895	7477/3696/1969/857/4643/7048/23493/4851
GO:0021675	nerve development	8/401	0.00025986	0.005504895	7020/5457/1959/390992/8929/4908/401/55636
GO:0071675	regulation of mononuclear cell migration	6/401	0.000271335	0.005719527	653/3958/196527/7057/158747/5919
GO:0061351	neural precursor cell proliferation	11/401	0.000290488	0.006093091	2637/10763/7424/5454/7005/2787/58158/6095/4036/7474/4851
GO:0019318	hexose metabolic process	15/401	0.000299171	0.006236928	2103/7021/5239/57103/85414/3636/4124/5167/2820/6095/5366/2597/5443/51363/8277
GO:0008593	regulation of Notch signaling pathway	9/401	0.000300275	0.006236928	10631/441478/8788/3955/182/4854/3815/23493/4851
GO:0035116	embryonic hindlimb morphogenesis	5/401	0.000304497	0.00629392	4488/4487/340419/55636/4851
GO:0035148	tube formation	11/401	0.000327494	0.006731845	653/23242/57167/6788/7052/4036/7474/6423/151449/79191/4851
GO:0007626	locomotory behavior	13/401	0.000328845	0.006731845	4009/83715/6900/127343/57626/793/7545/3251/4986/1136/10882/7200/55636
GO:0042733	embryonic digit morphogenesis	7/401	0.000340465	0.006909204	4488/4487/4038/341640/7474/6423/4851
GO:0007611	learning or memory	15/401	0.000340755	0.006909204	4009/6900/5176/793/54207/1959/4986/2353/64220/4908/10882/5764/3815/3678/27023
GO:0007628	adult walking behavior	5/401	0.000361441	0.007293917	6900/57626/7545/7200/55636
GO:0051048	negative regulation of secretion	14/401	0.000365056	0.007332124	7114/10874/100/5733/4986/80763/28984/2335/9607/55366/10385/7200/3162/4851
GO:0003158	endothelium development	10/401	0.000397929	0.007936057	7477/9076/4478/64102/3397/182/81575/5764/23493/4851
GO:0010463	mesenchymal cell proliferation	6/401	0.000398877	0.007936057	4487/10736/79633/7474/5764/7048
GO:0009798	axis specification	8/401	0.000400717	0.007936057	23242/441478/10736/4091/50805/7474/23493/4851
GO:0021783	preganglionic parasympathetic fiber development	4/401	0.000414318	0.008167442	7020/1959/390992/401
GO:0090659	walking behavior	5/401	0.00042608	0.00836059	6900/57626/7545/7200/55636
GO:0048806	genitalia development	6/401	0.000450428	0.008797819	653/64220/4036/7474/55366/55636
GO:0060560	developmental growth involved in morphogenesis	14/401	0.000456672	0.008879043	7477/23242/10631/223117/127833/5458/2335/8239/780/7474/6423/7048/1630/4851
GO:0050678	regulation of epithelial cell proliferation	19/401	0.000460366	0.008910172	653/5176/8862/8573/7424/441478/8837/28984/64102/3397/7057/4088/7474/857/11331/5764/6423/3162/4851
GO:0006939	smooth muscle contraction	9/401	0.000465251	0.008963981	5024/10874/100/5733/59/80763/1136/857/3815
GO:1904018	positive regulation of vasculature development	14/401	0.000477173	0.009152268	79924/7424/8837/8817/3696/7057/7474/285/6423/7048/3815/3678/3162/4851
GO:0045598	regulation of fat cell differentiation	10/401	0.000480613	0.009176912	4488/6788/182/5167/252995/6095/5919/4088/7474/6423
GO:0008217	regulation of blood pressure	12/401	0.000497702	0.009402705	10631/8862/79924/7424/10874/59/81285/80763/9607/4088/5443/3162
GO:0044319	wound healing, spreading of cells	5/401	0.000499063	0.009402705	4488/389/407006/780/3678
GO:0090505	epiboly involved in wound healing	5/401	0.000499063	0.009402705	4488/389/407006/780/3678
GO:0060070	canonical Wnt signaling pathway	16/401	0.000501783	0.009412297	7477/6788/441478/4038/84133/284654/7479/83439/340419/4088/7474/27303/857/55366/6423/4851
GO:1904888	cranial skeletal system development	7/401	0.000504502	0.009421792	7020/4488/10736/158326/4088/10265/7048
GO:1904035	regulation of epithelial cell apoptotic process	8/401	0.000511461	0.009510054	79625/196527/8837/4760/28984/7057/2934/3162
GO:0007221	positive regulation of transcription of Notch receptor target	4/401	0.000523628	0.00965198	23286/26508/4854/4851
GO:0030540	female genitalia development	4/401	0.000523628	0.00965198	64220/4036/7474/55636
GO:0006006	glucose metabolic process	13/401	0.000531476	0.009754419	2103/7021/5239/57103/85414/3636/5167/2820/6095/5366/2597/5443/8277
GO:0050921	positive regulation of chemotaxis	10/401	0.000543287	0.009928395	7114/7424/196527/8817/7057/158747/5919/4908/4088/7474
GO:0010812	negative regulation of cell-substrate adhesion	7/401	0.000554008	0.010038145	10631/8573/182/7057/285/395/4851
GO:0050918	positive chemotaxis	7/401	0.000554008	0.010038145	64221/7424/3958/4908/4088/7474/285
GO:0051271	negative regulation of cellular component movement	19/401	0.000560557	0.010113777	653/928/5176/223117/100/8519/28984/182/1602/7057/407006/5314/7474/285/5764/51765/6423/3162/4851
GO:0090504	epiboly	5/401	0.000581062	0.010439496	4488/389/407006/780/3678
GO:0045599	negative regulation of fat cell differentiation	6/401	0.000636718	0.011391371	4488/182/5167/6095/4088/7474
GO:0010810	regulation of cell-substrate adhesion	13/401	0.000667214	0.011887017	79625/10631/8573/2335/182/7057/780/4088/285/395/5764/3678/4851
GO:0060603	mammary gland duct morphogenesis	5/401	0.000672764	0.011935954	4488/1969/780/7474/11331
GO:2001238	positive regulation of extrinsic apoptotic signaling pathway	6/401	0.000710505	0.012553238	6788/658/7057/5366/857/4118
GO:0032147	activation of protein kinase activity	17/401	0.000731202	0.012865527	7477/7852/6788/7867/7161/1647/1136/3654/55765/28984/7057/9607/4908/7474/51765/7048/3815
GO:0048566	embryonic digestive tract development	5/401	0.000774876	0.013522226	10736/100/348093/64220/5919
GO:0061384	heart trabecula morphogenesis	5/401	0.000774876	0.013522226	653/2274/55636/23493/4851
GO:0007548	sex differentiation	15/401	0.000796352	0.013590383	653/10184/3955/658/1602/64220/4036/7474/55366/10265/6423/3815/55636/8531/10549
GO:0003215	cardiac right ventricle morphogenesis	4/401	0.000801124	0.013590383	182/55636/23493/4851
GO:0023019	signal transduction involved in regulation of gene expression	4/401	0.000801124	0.013590383	4488/4487/4760/4088
GO:0030903	notochord development	4/401	0.000801124	0.013590383	23242/1969/286204/7474
GO:0060039	pericardium development	4/401	0.000801124	0.013590383	653/4088/7474/4851
GO:0072234	metanephric nephron tubule development	4/401	0.000801124	0.013590383	7477/793/79633/55366
GO:2000678	negative regulation of transcription regulatory region DNA binding	4/401	0.000801124	0.013590383	4488/7114/4487/23493
GO:0071453	cellular response to oxygen levels	12/401	0.000810357	0.013692456	79625/8837/3654/57103/28984/5458/6095/5366/857/5764/3162/4851
GO:0001889	liver development	10/401	0.000818311	0.013772203	23242/9076/100/8837/4124/1373/4088/5764/3162/4851
GO:0030501	positive regulation of bone mineralization	5/401	0.000888121	0.014829878	7020/196527/658/2201/4088
GO:0035909	aorta morphogenesis	5/401	0.000888121	0.014829878	7021/182/55636/23493/4851
GO:0061008	hepaticobiliary system development	10/401	0.000966909	0.016082421	23242/9076/100/8837/4124/1373/4088/5764/3162/4851
GO:0043392	negative regulation of DNA binding	6/401	0.000972021	0.016104539	4488/7114/4487/3397/3162/23493
GO:0001101	response to acid chemical	17/401	0.000989722	0.016334245	56603/7477/23677/928/6472/5176/9076/81285/1592/7479/1571/1373/2934/7474/5764/3856/54997
GO:0007179	transforming growth factor beta receptor signaling pathway	12/401	0.001066683	0.017419919	2662/658/2353/3397/4091/8239/7057/2201/4088/857/390598/7048

GO:0048511	rhythmic process	15/401	0.001071869	0.017419919	5176/10874/1959/100/4986/658/50865/3397/10887/8914/6095/9607/10495/55366/5764
GO:0044344	cellular response to fibroblast growth factor stimulus	10/401	0.001077743	0.017419919	10631/143282/8862/8817/2261/7057/79633/1373/7474/9965
GO:0046660	female sex differentiation	9/401	0.001082571	0.017419919	10184/3955/658/1602/64220/4036/7474/3815/55636
GO:0048570	notochord morphogenesis	3/401	0.001084142	0.017419919	1969/286204/7474
GO:0099566	regulation of postsynaptic cytosolic calcium ion concentration	3/401	0.001084142	0.017419919	793/3708/7474
GO:1903909	regulation of receptor clustering	3/401	0.001084142	0.017419919	4038/2934/5764
GO:0021954	central nervous system neuron development	7/401	0.001104209	0.017675651	2637/6900/3251/8929/7474/113246/1630
GO:0046621	negative regulation of organ growth	5/401	0.001150968	0.018286646	80014/23286/6788/7161/7048
GO:1902042	negative regulation of extrinsic apoptotic signaling pathway via death domain receptors	5/401	0.001150968	0.018286646	653/8837/407006/6423/3162
GO:0035265	organ growth	12/401	0.001165808	0.018432699	80014/4488/23286/6788/7161/4880/2261/340419/7048/8531/23493/4851
GO:0071425	hematopoietic stem cell proliferation	4/401	0.001168818	0.018432699	2120/407006/7474/6423
GO:0031589	cell-substrate adhesion	17/401	0.001202281	0.018890451	79625/10631/8573/11149/3397/4091/2335/182/7057/158326/780/4088/285/395/5764/3678/4851
GO:0021782	glial cell development	8/401	0.001226965	0.019207414	928/6900/7431/4919/5454/26227/5453/2934
GO:0051098	regulation of binding	18/401	0.001232244	0.019214739	5457/64641/4488/10763/7114/4487/3958/6788/3955/4760/5081/3397/5458/4088/7474/857/3162/23493
GO:0048771	tissue remodeling	11/401	0.001236458	0.019214739	7052/3988/1969/760/182/9607/857/55366/1945/5745/55636
GO:0048661	positive regulation of smooth muscle cell proliferation	8/401	0.001311299	0.020303681	7052/3654/348093/7057/407006/4854/7048/3162
GO:0043062	extracellular structure organization	19/401	0.001316901	0.020316539	79625/10631/8837/255631/3696/28984/2335/7057/2201/780/4088/9509/4868/4643/9507/6423/3678/10549/4851
GO:0051101	regulation of DNA binding	9/401	0.001369411	0.021001462	5457/4488/7114/4487/4760/3397/5458/3162/23493
GO:0007163	establishment or maintenance of cell polarity	12/401	0.001387057	0.021001462	56603/7477/23286/58480/389/3996/4478/56647/2195/286204/2934/7474
GO:0043576	regulation of respiratory gaseous exchange	4/401	0.001390891	0.021001462	30012/8862/8929/401
GO:0051446	positive regulation of meiotic cell cycle	4/401	0.001390891	0.021001462	4488/4487/3955/7474
GO:0072170	metanephric tubule development	4/401	0.001390891	0.021001462	7477/793/79633/55366
GO:0072243	metanephric nephron epithelium development	4/401	0.001390891	0.021001462	7477/793/79633/55366
GO:0031652	positive regulation of heat generation	3/401	0.001466809	0.021619312	8862/10874/5733
GO:0060439	trachea morphogenesis	3/401	0.001466809	0.021619312	7477/340419/7048
GO:0072015	glomerular visceral epithelial cell development	3/401	0.001466809	0.021619312	182/4868/4643
GO:0072070	loop of Henle development	3/401	0.001466809	0.021619312	7477/182/79191
GO:1901201	regulation of extracellular matrix assembly	3/401	0.001466809	0.021619312	28984/4088/4851
GO:0045746	negative regulation of Notch signaling pathway	5/401	0.001467351	0.021619312	441478/8788/3955/4854/23493
GO:1903053	regulation of extracellular matrix organization	5/401	0.001467351	0.021619312	8837/28984/780/4088/4851
GO:0071774	response to fibroblast growth factor	10/401	0.001474097	0.021643818	10631/143282/8862/8817/2261/7057/79633/1373/7474/9965
GO:0001838	embryonic epithelial tube formation	9/401	0.001534185	0.022448659	653/23242/57167/6788/4036/7474/6423/151449/79191
GO:0048871	multicellular organismal homeostasis	21/401	0.001546259	0.022547841	56603/2103/64641/7852/7021/8862/9076/10874/5733/445582/3988/4760/8929/55765/3397/760/9607/857/55366/5745/4851
GO:0005996	monosaccharide metabolic process	15/401	0.001578721	0.022942635	2103/7021/5239/57103/85414/3636/4124/5167/2820/6095/5366/2597/5443/51363/8277
GO:0000187	activation of MAPK activity	10/401	0.001629877	0.023602906	7477/7852/7867/7161/3654/55765/7057/4908/7474/3815
GO:0045672	positive regulation of osteoclast differentiation	4/401	0.001640784	0.023602906	5457/2353/5458/760
GO:2001026	regulation of endothelial cell chemotaxis	4/401	0.001640784	0.023602906	7114/8817/7057/4851
GO:0090100	positive regulation of transmembrane receptor protein serine/threonine kinase signaling pathway	8/401	0.001804669	0.025872991	653/4488/2662/4487/7057/286204/151449/4851
GO:0017145	stem cell division	5/401	0.001843487	0.026252729	2103/7005/6423/3815/4851
GO:0035136	forelimb morphogenesis	5/401	0.001843487	0.026252729	7020/7021/4488/4487/340419
GO:1903845	negative regulation of cellular response to transforming growth factor beta stimulus	7/401	0.001875286	0.026518683	8837/4091/2201/4088/857/390598/7048
GO:0035270	endocrine system development	9/401	0.001911448	0.026518683	653/4487/7068/5454/4760/219409/64220/4088/7474
GO:0001841	neural tube formation	8/401	0.001918932	0.026518683	653/23242/57167/6788/4036/7474/6423/151449
GO:0006721	terpenoid metabolic process	8/401	0.001918932	0.026518683	56603/1381/1592/2239/1571/64220/5919/4036
GO:0032461	positive regulation of protein oligomerization	4/401	0.001920238	0.026518683	389/129293/388630/5366
GO:0060343	trabecula formation	4/401	0.001920238	0.026518683	4488/2274/2201/23493
GO:0006563	L-serine metabolic process	3/401	0.001924442	0.026518683	29968/6472/26227
GO:0060546	negative regulation of necroptotic process	3/401	0.001924442	0.026518683	8837/857/8531
GO:0060900	embryonic camera-type eye formation	3/401	0.001924442	0.026518683	7020/64220/7474
GO:0072310	glomerular epithelial cell development	3/401	0.001924442	0.026518683	182/4868/4643
GO:0002685	regulation of leukocyte migration	11/401	0.001951225	0.026801022	653/7424/3958/196527/100/4478/7057/158747/5919/7474/3162
GO:0060828	regulation of canonical Wnt signaling pathway	13/401	0.002008095	0.027296119	6788/441478/4038/84133/284654/340419/4088/7474/27303/857/55366/6423/4851
GO:0014031	mesenchymal cell development	7/401	0.002012912	0.027296119	2637/223117/26508/8929/182/23493/4851
GO:0060415	muscle tissue morphogenesis	7/401	0.002012912	0.027296119	5457/5081/4036/7474/55636/23493/4851
GO:0110110	positive regulation of animal organ morphogenesis	7/401	0.002012912	0.027296119	7020/8837/182/55366/5764/7048/4851
GO:0090130	tissue migration	17/401	0.002025615	0.027381171	5176/7114/7424/59/8817/28984/3397/1969/7057/407006/7474/285/7048/3815/5168/3162/4851
GO:0022037	metencephalon development	8/401	0.002038823	0.027438508	4009/2637/869/57626/4760/6095/401/5764
GO:1903706	regulation of hemopoiesis	19/401	0.002053374	0.027438508	56603/5457/3958/441478/100/6938/4343/7161/2353/5458/760/182/7057/9607/407006/10385/7048/54997/4851
GO:0001953	negative regulation of cell-matrix adhesion	5/401	0.00205594	0.027438508	10631/8573/182/7057/395
GO:0099054	presynapse assembly	5/401	0.00205594	0.027438508	869/4038/266727/2239/7474
GO:0060326	cell chemotaxis	15/401	0.002062077	0.027438508	7852/10451/7114/7424/3958/23765/196527/8817/1969/7057/158747/5919/7474/3815/4851
GO:0045666	positive regulation of neuron differentiation	17/401	0.002086028	0.027670735	653/23242/79625/5176/26508/6938/8837/127833/4760/8929/5458/2335/407006/7474/5764/151449/79191
GO:0043534	blood vessel endothelial cell migration	11/401	0.002128223	0.028142779	7114/7424/8817/28984/3397/1969/7057/407006/285/3162/4851
GO:0032103	positive regulation of response to external stimulus	15/401	0.002200476	0.028968983	7114/7424/23765/7052/196527/5733/55601/8817/7057/158747/5919/407006/4908/4088/7474
GO:0006940	regulation of smooth muscle contraction	6/401	0.002211113	0.028968983	10874/100/80763/1136/857/3815
GO:0051148	negative regulation of muscle cell differentiation	6/401	0.002211113	0.028968983	4487/8837/348093/407006/23493/4851
GO:0045992	negative regulation of embryonic development	4/401	0.002230973	0.029050409	441478/3955/7474/6423
GO:0050926	regulation of positive chemotaxis	4/401	0.002230973	0.029050409	7424/4908/4088/285
GO:0072175	epithelial tube formation	9/401	0.002240314	0.029083101	653/23242/57167/6788/4036/7474/6423/151449/79191
GO:0001708	cell fate specification	7/401	0.002311511	0.029916155	5457/30012/167826/5454/10736/6423/4851
GO:2001233	regulation of apoptotic signaling pathway	18/401	0.002326151	0.030014394	653/4487/3958/6788/8837/7161/658/7057/407006/2934/4088/5366/857/4118/83596/6423/8531/3162
GO:0043535	regulation of blood vessel endothelial cell migration	10/401	0.002391876	0.030717959	7114/7424/8817/28984/1969/7057/407006/285/3162/4851
GO:0001667	ameboidal-type cell migration	20/401	0.002397861	0.030717959	2637/5176/7114/7424/223117/8817/8929/28984/3397/1969/7057/286204/407006/7474/285/7048/3815/5168/3162/4851

GO:0098742	cell-cell adhesion via plasma-membrane adhesion molecules	14/401	0.00240232	0.030717959	64221/6900/869/9076/54549/266727/2195/2239/79633/407006/26167/54510/7048/3678
GO:0046620	regulation of organ growth	8/401	0.002434183	0.030921045	80014/23286/6788/7161/7048/8531/23493/4851
GO:0009111	vitamin catabolic process	3/401	0.002461774	0.030921045	56603/1381/1592
GO:0031650	regulation of heat generation	3/401	0.002461774	0.030921045	8862/10874/5733
GO:0070307	lens fiber cell development	3/401	0.002461774	0.030921045	7477/7431/1969
GO:0072109	glomerular mesangium development	3/401	0.002461774	0.030921045	8837/59/4851
GO:0097152	mesenchymal cell apoptotic process	3/401	0.002461774	0.030921045	4488/4487/2120
GO:0070169	positive regulation of biomineral tissue development	5/401	0.002533622	0.031636842	7020/196527/658/2201/4088
GO:0099172	presynapse organization	5/401	0.002533622	0.031636842	869/4038/266727/2239/7474
GO:0003148	outflow tract septum morphogenesis	4/401	0.002574687	0.031869238	4488/4091/4036/7048
GO:0072207	metanephric epithelium development	4/401	0.002574687	0.031869238	7477/793/79633/55366
GO:1902895	positive regulation of pri-miRNA transcription by RNA polymerase II	4/401	0.002574687	0.031869238	8862/2353/4091/4088
GO:1905330	regulation of morphogenesis of an epithelium	9/401	0.002612698	0.032164948	4919/10736/84133/2239/407006/7474/55366/11331/6423
GO:0043433	negative regulation of DNA-binding transcription factor activity	10/401	0.002621239	0.032164948	4488/7114/26508/3654/3397/5458/4796/5314/11331/3162
GO:0071456	cellular response to hypoxia	10/401	0.002621239	0.032164948	79625/8837/3654/57103/28984/6095/5366/5764/3162/4851
GO:0001523	retinoid metabolic process	7/401	0.002643146	0.032247896	56603/1381/1592/2239/64220/5919/4036
GO:0070167	regulation of biomineral tissue development	7/401	0.002643146	0.032247896	7020/196527/658/5167/2201/4088/4851
GO:0050954	sensory perception of mechanical stimulus	10/401	0.002742347	0.033352737	7020/5077/83715/4919/7068/5458/63895/4036/3815/55636
GO:0007584	response to nutrient	12/401	0.002749368	0.033352737	56603/10631/5024/5251/100/7222/1592/2934/5764/6423/7048/3162
GO:0002063	chondrocyte development	5/401	0.002800465	0.033564284	4488/8817/658/6423/7048
GO:0048701	embryonic cranial skeleton morphogenesis	5/401	0.002800465	0.033564284	7020/10736/4088/10265/7048
GO:0060412	ventricular septum morphogenesis	5/401	0.002800465	0.033564284	26508/7474/7048/23493/4851
GO:0007585	respiratory gaseous exchange	6/401	0.002814103	0.033564284	30012/8862/8929/4124/1602/401
GO:0033627	cell adhesion mediated by integrin	6/401	0.002814103	0.033564284	100/3696/1969/6423/3678/54997
GO:0042246	tissue regeneration	6/401	0.002814103	0.033564284	10631/8837/5081/2934/7048/4851
GO:0048709	oligodendrocyte differentiation	7/401	0.002822081	0.03356542	7852/928/6900/7161/85414/2934/4851
GO:0050680	negative regulation of epithelial cell proliferation	10/401	0.00286789	0.034015254	5176/8573/28984/64102/7057/7474/857/11331/5764/6423
GO:0002065	columnar/cuboidal epithelial cell differentiation	8/401	0.002887218	0.034149375	653/4760/56647/57103/182/7474/23493/4851
GO:0007162	negative regulation of cell adhesion	14/401	0.00293173	0.034579799	10631/928/8573/3902/3958/441478/28984/182/7057/407006/285/395/10385/4851
GO:0002791	regulation of peptide secretion	20/401	0.002995096	0.035229613	10631/7021/8862/7114/7424/23765/3708/10874/81285/4760/28984/2335/9607/2044/7474/5366/2597/10385/7200/55636
GO:0048644	muscle organ morphogenesis	7/401	0.003010156	0.035309214	5457/5081/4036/7474/55636/23493/4851
GO:0014013	regulation of gliogenesis	8/401	0.003051992	0.035622442	7852/6900/7161/85414/407006/4036/5764/4851
GO:0021794	thalamus development	3/401	0.003083127	0.035622442	2637/5764/27023
GO:0045836	positive regulation of meiotic nuclear division	3/401	0.003083127	0.035622442	4488/4487/7474
GO:1901213	regulation of transcription from RNA polymerase II promoter involved in heart development	3/401	0.003083127	0.035622442	4488/4487/4851
GO:0021537	telencephalon development	13/401	0.003086759	0.035622442	4009/6900/340260/5454/3251/4760/79633/2044/7474/113246/5992/55636/27023
GO:0030857	negative regulation of epithelial cell differentiation	5/401	0.003087055	0.035622442	4488/3397/182/857/4851
GO:0044262	cellular carbohydrate metabolic process	14/401	0.003128143	0.035999013	2103/4952/5239/9348/57103/85414/5836/5167/2820/6095/5366/5745/5443/55586
GO:0001656	metanephros development	7/401	0.003207672	0.036617341	7477/7021/793/10736/79633/55366/79191
GO:0009953	dorsal/ventral pattern formation	7/401	0.003207672	0.036617341	4010/4038/658/5081/7479/4091/5992
GO:0033273	response to vitamin	7/401	0.003207672	0.036617341	56603/10631/5251/100/1592/2934/5764
GO:0008543	fibroblast growth factor receptor signaling pathway	8/401	0.003223994	0.036705257	143282/8862/8817/2261/7057/79633/7474/9965
GO:0007416	synapse assembly	10/401	0.003272281	0.037155665	5457/869/54549/7162/4038/266727/57633/2239/26167/7474
GO:0070306	lens fiber cell differentiation	4/401	0.00336771	0.038137524	7477/7431/1969/4088
GO:0055010	ventricular cardiac muscle tissue morphogenesis	5/401	0.003394201	0.038335567	5457/4036/55636/23493/4851
GO:0010594	regulation of endothelial cell migration	12/401	0.003433767	0.038679844	5176/7114/7424/8817/28984/1969/7057/407006/7474/285/3162/4851
GO:0045639	positive regulation of myeloid cell differentiation	7/401	0.003632243	0.040807622	5457/2353/5458/760/182/407006/54997
GO:0030534	adult behavior	9/401	0.003673122	0.041158294	6900/127343/57626/7545/4986/1136/9607/7200/55636
GO:0022604	regulation of cell morphogenesis	20/401	0.003712361	0.041273575	10631/6900/223117/58480/5454/389/55843/11149/127833/4038/1136/4478/3397/5458/2335/7474/5764/3815/5168/1630
GO:0048660	regulation of smooth muscle cell proliferation	10/401	0.003720797	0.041273575	8862/7052/3654/4880/348093/7057/407006/4854/7048/3162
GO:0051445	regulation of meiotic cell cycle	5/401	0.003722712	0.041273575	4488/4487/3955/4880/7474
GO:0010632	regulation of epithelial cell migration	14/401	0.003783022	0.041273575	5176/7114/7424/8817/28984/1969/7057/407006/7474/285/7048/5168/3162/4851
GO:0007635	chemosensory behavior	3/401	0.003792425	0.041273575	4009/5024/55636
GO:0043101	purine-containing compound salvage	3/401	0.003792425	0.041273575	56952/3251/100
GO:0002088	lens development in camera-type eye	6/401	0.003799728	0.041273575	7477/7431/1969/4088/7474/7048
GO:0030500	regulation of bone mineralization	6/401	0.003799728	0.041273575	7020/196527/658/5167/2201/4088
GO:0055008	cardiac muscle tissue morphogenesis	6/401	0.003799728	0.041273575	5457/4036/7474/55636/23493/4851
GO:0060395	SMAD protein signal transduction	6/401	0.003799728	0.041273575	653/7431/2662/2353/4088/151449
GO:0071300	cellular response to retinoic acid	6/401	0.003799728	0.041273575	56603/7477/5176/7479/7474/54997
GO:1903524	positive regulation of blood circulation	6/401	0.003799728	0.041273575	8862/10874/100/81285/857/23493
GO:0010631	epithelial cell migration	16/401	0.003813367	0.041286049	5176/7114/7424/8817/28984/3397/1969/7057/407006/7474/285/7048/3815/5168/3162/4851
GO:0001569	branching involved in blood vessel morphogenesis	4/401	0.003820268	0.041286049	2637/441478/6423/7048
GO:0007605	sensory perception of sound	9/401	0.003848749	0.041488537	7020/5077/83715/4919/7068/5458/4036/3815/55636
GO:0016101	diterpenoid metabolic process	7/401	0.003859911	0.041503791	56603/1381/1592/2239/64220/5919/4036
GO:0036294	cellular response to decreased oxygen levels	10/401	0.004045934	0.043154887	79625/8837/3654/57103/28984/6095/5366/5764/3162/4851
GO:0048659	smooth muscle cell proliferation	10/401	0.004045934	0.043154887	8862/7052/3654/4880/348093/7057/407006/4854/7048/3162
GO:2001235	positive regulation of apoptotic signaling pathway	10/401	0.004045934	0.043154887	4487/6788/7161/658/7057/2934/4088/5366/857/4118
GO:0060976	coronary vasculature development	5/401	0.004073393	0.043154887	8862/4091/4036/23493/4851
GO:0010975	regulation of neuron projection development	20/401	0.004074275	0.043154887	653/23242/79625/6900/7431/5176/223117/5454/8837/127833/4038/1136/3397/5458/2335/407006/7474/5764/6423/1630
GO:0050900	leukocyte migration	20/401	0.004074275	0.043154887	653/7852/10451/2995/7424/3958/23765/196527/100/4478/2335/7057/158747/5919/7474/857/285/3815/3678/3162
GO:0090132	epithelium migration	16/401	0.004142791	0.043771721	5176/7114/7424/8817/28984/3397/1969/7057/407006/7474/285/7048/3815/5168/3162/4851
GO:0001704	formation of primary germ layer	8/401	0.004199862	0.044264878	10736/1969/2335/286204/4088/7474/6423/3678
GO:0001659	temperature homeostasis	10/401	0.004216712	0.044271859	64641/7852/8862/10874/5733/8929/3397/857/55366/4851
GO:0048863	stem cell differentiation	11/401	0.004221319	0.044271859	2103/2637/4488/4487/223117/6938/7161/8929/182/3815/4851
GO:0030522	intracellular receptor signaling pathway	14/401	0.00427866	0.044762986	56603/2103/7068/9745/26508/55601/2274/3654/1592/55765/5458/6095/11331/7105
GO:0043406	positive regulation of MAP kinase activity	13/401	0.004310748	0.044988145	7477/7852/7867/7161/1647/8817/3654/55765/7057/9607/4908/7474/3815
GO:0060993	kidney morphogenesis	7/401	0.004347568	0.045261474	7477/793/51301/10736/79633/55366/79191
GO:0031667	response to nutrient levels	20/401	0.00436456	0.045295602	56603/10631/5024/5251/100/81788/7222/80763/1592/440738/9607/1373/2934/5366/5443/5764/51765/6423/7048/3162
GO:0060350	endochondral bone morphogenesis	6/401	0.004378864	0.045295602	8817/658/4880/3636/2261/7048
GO:0045766	positive regulation of angiogenesis	11/401	0.004382759	0.045295602	79924/7424/8817/3696/7057/7474/285/6423/7048/3678/3162

GO:1903844	regulation of cellular response to transforming growth factor beta stimulus	8/401	0.004419726	0.045567055	8837/4091/7057/2201/4088/857/390598/7048
GO:0045668	negative regulation of osteoblast differentiation	5/401	0.004447047	0.045737986	2662/3397/4091/4088/4851
GO:0050808	synapse organization	17/401	0.004502371	0.046195414	7477/5457/4009/22866/6900/869/54549/7162/25978/4038/266727/57633/2239/26167/7474/10882/5764
GO:0003222	ventricular trabecula myocardium morphogenesis	3/401	0.004593218	0.046677618	55636/23493/4851
GO:0035988	chondrocyte proliferation	3/401	0.004593218	0.046677618	10736/658/2261
GO:0048385	regulation of retinoic acid receptor signaling pathway	3/401	0.004593218	0.046677618	56603/9745/1592
GO:0048521	negative regulation of behavior	3/401	0.004593218	0.046677618	10874/100/7200
GO:0030316	osteoclast differentiation	7/401	0.004608183	0.046718194	5457/2353/1969/5458/760/9607/1945
GO:0001707	mesoderm formation	6/401	0.004691679	0.047451713	10736/1969/286204/4088/7474/6423
GO:0040013	negative regulation of locomotion	17/401	0.004739002	0.047816751	653/5176/223117/100/8519/28984/182/1602/7057/407006/7474/285/5764/51765/6423/3162/4851
GO:0030513	positive regulation of BMP signaling pathway	4/401	0.004845333	0.048204268	4488/4487/286204/4851
GO:0035587	purinergic receptor signaling pathway	4/401	0.004845333	0.048204268	6900/5024/196527/100
GO:0035767	endothelial cell chemotaxis	4/401	0.004845333	0.048204268	7114/8817/7057/4851
GO:0045987	positive regulation of smooth muscle contraction	4/401	0.004845333	0.048204268	10874/100/80763/3815
GO:0048713	regulation of oligodendrocyte differentiation	4/401	0.004845333	0.048204268	7852/7161/85414/4851
GO:0061036	positive regulation of cartilage development	4/401	0.004845333	0.048204268	8817/658/4088/7474
GO:0021549	cerebellum development	7/401	0.00488041	0.048440058	4009/2637/869/57626/4760/6095/5764
GO:0030336	negative regulation of cell migration	15/401	0.004954922	0.04895141	653/5176/100/8519/28984/182/1602/7057/407006/285/5764/51765/6423/3162/4851
GO:0070372	regulation of ERK1 and ERK2 cascade	15/401	0.004954922	0.04895141	4919/8837/8817/1969/2335/2261/407006/5314/780/2044/11331/10385/3815/9965/4851
GO:0043627	response to estrogen	6/401	0.005020631	0.049485759	4986/760/4091/857/7048/3162
GO:0031012	extracellular matrix	26/410	1.43E-05	0.006048332	79625/10631/64093/2662/143282/5176/8573/3958/7052/255631/23452/57633/5359/2239/2335/341640/7057/5919/158326/2201/7474/9509/9507/5764/6423/7980
GO:0046658	anchored component of plasma membrane	7/410	0.000196047	0.022539528	6900/266727/594855/2239/5314/762/285704
GO:0009897	external side of plasma membrane	19/410	0.00021032	0.022539528	7852/928/3902/3977/100/619207/2239/3597/7057/4036/10495/5314/2044/762/10385/7048/3815/3678/115650
GO:0062023	collagen-containing extracellular matrix	18/410	0.000229015	0.022539528	10631/64093/2662/5176/8573/3958/7052/255631/23452/2239/2335/341640/7057/158326/2201/9509/5764/6423
GO:0043235	receptor complex	20/410	0.000267056	0.022539528	4919/5024/3977/1136/658/3654/3696/1969/3597/2261/4036/780/4088/2044/4854/5745/7048/3815/3678/4851
GO:0045177	apical part of cell	19/410	0.0003399	0.023906278	928/9076/4478/2195/760/2335/182/286204/79633/4036/5314/4118/5745/762/1184/57477/51765/121441/4851
GO:0000982	transcription factor activity, RNA polymerase II proximal promoter sequence-specific DNA binding	33/393	1.84E-09	6.07E-07	7020/5457/2103/64641/7050/7021/4487/54626/253738/9745/5454/7545/1959/26508/10736/7546/6938/390992/7161/4760/2353/8929/5359/219409/137209/5458/2120/2000/30818/7764/2118/5992/23493
GO:0001228	DNA-binding transcription activator activity, RNA polymerase II-specific	33/393	2.05E-09	6.07E-07	7020/5457/2103/64641/4009/2637/7021/4487/253738/4807/5454/7545/1959/26508/10736/7546/6938/7161/4760/2353/8929/5359/219409/137209/5458/2120/6095/2000/4088/2118/5992/23493/4851
GO:0001077	proximal promoter DNA-binding transcription activator activity, RNA polymerase II-specific	25/393	1.49E-08	2.93E-06	7020/5457/64641/7021/253738/5454/7545/1959/26508/10736/7546/6938/7161/4760/2353/8929/5359/219409/137209/5458/2120/2000/2118/5992/23493
GO:0000983	transcription factor activity, RNA polymerase II core promoter sequence-specific DNA binding	8/393	3.05E-08	4.51E-06	7021/4010/6875/26508/5081/1602/4088/23493
GO:0046332	SMAD binding	10/393	1.11E-05	0.00130947	7050/6938/658/2353/28984/4091/8239/4088/390598/7048
GO:0005109	frizzled binding	7/393	2.15E-05	0.002120686	7477/54894/84133/284654/7479/340419/7474
GO:0048018	receptor ligand activity	25/393	7.61E-05	0.005794044	7477/653/2662/8862/79924/7424/3958/223117/80763/8817/4880/7479/112616/182/252995/9607/4908/123920/7474/5443/5764/7200/6423/151449/9965
GO:0019199	transmembrane receptor protein kinase activity	9/393	7.84E-05	0.005794044	4919/658/1969/2261/780/2044/1945/7048/3815
GO:0070410	co-SMAD binding	4/393	0.000104409	0.006444338	7050/4091/8239/4088
GO:0001227	DNA-binding transcription repressor activity, RNA polymerase II-specific	17/393	0.000109041	0.006444338	7020/7050/84295/4488/127343/4487/54626/9745/26508/390992/5458/2120/1602/30818/7764/8531/23493
GO:0004089	carbonate dehydratase activity	4/393	0.00027303	0.01466914	767/23632/760/762
GO:0003714	transcription corepressor activity	15/393	0.000305273	0.015034717	5457/7050/7021/167826/54626/7068/26508/390992/5458/4796/5932/30818/390598/8531/23493
GO:0004714	transmembrane receptor protein tyrosine kinase activity	7/393	0.000539371	0.024520637	4919/1969/2261/780/2044/1945/3815
GO:0005160	transforming growth factor beta receptor binding	6/393	0.000911033	0.038458588	653/2662/4091/4088/151449/7048
GO:0017147	Wnt-protein binding	5/393	0.001144465	0.045091934	147495/4919/129293/388630/6423

Suppl. Table 2. Summary of the sources and dilutions of primary antibodies

Antibody	Species	Catalog number	Company	Work concentration	Application
SOX2	mouse	MAB2018	R&D	1:1000	neuroepithelium marker
TUJ1	rabbit	PRB-435P	Sigma	1:1000	pan neural marker
ZO-1	rabbit	40-2200	Invitrogen	1:500	one of junction proteins marker
N-Cadherin	sheep	AF6426	R&D	1:500	one of adherens junction proteins marker
β-catnin	goat	CST9582P	Cell Signaling	1:500	one of adherens junction proteins marker
Ki67	rabbit	LV1825852	Millipore	1:1000	proliferative cells marker
PH3	mouse	9706	Cell Signaling Technology	1:1000	cells undergoing mitosis marker
Arl13b	rabbit	75-287	NIH NeuroMab Facility	1:500	cilia marker
PAX6	rabbit	901301	Biolegend	1:1000	cortical progenitor marker
TBR2	sheep	AF6166	R&D	1:500	intermediate progenitor marker
GFP	chicken	ab6556-25	Abcam	1:1000	labeled gene marker
TBR1	rabbit	ab31940	Abcam	1:500	one of cortical projection neurons marker
CTIP2	rat	ab18465	Abcam	1:1000	one of cortical projection neurons marker
SATB2	mouse	SC81376	Santa Cruz	1:800	one of cortical projection neurons marker
NEUN	mouse	NG1898237	Millipore	1:4000	mature neurons marker
DCX	goat	SC8066	Santa Cruz	1:1000	postmitotic migrating neurons marker
vGlut1	mouse	ab180188	Abcam	1:500	glutamatergic synapses marker
Bassoon	mouse	ab82958	Abcam	1:1000	pre-synapse marker

PSD95	rabbit	ab18258	Abcam	1:2000	post-synapse marker
3B5h10	mouse	P1874	Sigma	1:5000	HTT assemblies marker
Clathrin	rabbit	ab21679	Abcam	1:1000	vesicular marker
GM130	rabbit	ab52649	Abcam	1:1000	Golgi marker
ARF1	rabbit	10790-1-AP	Proteintech	1：1000	ADP-ribosylation factor 1 marker
ARF1	mouse	MA5-38445	Invitrogen	1:500	ADP-ribosylation factor 1 marker

Suppl. Table 3. Summary of the sources and dilutions of secondary antibodies

Antibody	Company	Work concentration
Alexa Fluro 488 Donkey anti-mouse IgG	Invitrogen	1： 1000
Alexa Fluro 594 Donkey anti-mouse IgG	Invitrogen	1： 1000
Alexa Fluro 594 Donkey anti-rabbit IgG	Chemicon	1： 1000
Alexa Fluro 488 Donkey anti-rabbit IgG	Invitrogen	1： 1000
Alexa Fluro 594 Donkey anti-goat IgG	Invitrogen	1： 1000
Alexa Fluro 488 Donkey anti-rat IgG	Invitrogen	1： 1000
Alexa Fluro 488 Donkey anti-sheep IgG	Invitrogen	1： 1000
Cy5 AffiniPure Donkey Anti-Goat IgG (H+L)	Jackson	1:500
Cy5 AffiniPure Donkey Anti-mouse IgG (H+L)	Jackson	1:500
Cy5 AffiniPure Donkey Anti-Rabbit IgG (H+L)	Jackson	1:500
DAPI	Sigma	1： 1000
